# Joint bioinformatics analysis of underlying potential functions of hsa-let-7b-5p and core genes in human glioma

**DOI:** 10.1186/s12967-019-1882-7

**Published:** 2019-04-17

**Authors:** Xiaonan Xi, Yahui Chu, Ning Liu, Qianqian Wang, Zheng Yin, Yaxin Lu, Yue Chen

**Affiliations:** 1 College of Pharmacy, Nankai University, Tianjin, 300350 People’s Republic of China; 2 State Key Laboratory of Medicinal Chemical Biology, Nankai University, Tianjin, 300350 People’s Republic of China

**Keywords:** Glioma, MicroRNA, hsa-let-7b-5p, Biomarker, Bioinformatics analysis

## Abstract

**Background:**

Glioma accounts for a large proportion of cancer, and an effective treatment for this disease is still lacking because of the absence of specific driver molecules. Current challenges in the treatment of glioma are the accurate and timely diagnosis of brain glioma and targeted treatment plans. To investigate the diagnostic biomarkers and prospective role of miRNAs in the tumorigenesis and progression of glioma, we analyzed the expression of miRNAs and key genes in glioma based on The Cancer Genome Atlas database.

**Methods:**

Of the 701 cases that were downloaded, five were normal and 696 were glioma. Then, 1626 differentially expressed genes were identified, and 173 aberrantly expressed miRNAs were calculated by edgeR. GO and KEGG pathway enrichment analyses were performed using Cytoscape software. A coexpression network was built by weighted correlation network analysis (WGCNA). A cell scratch test and transwell, cell apoptosis and cell cycle assays were performed to validate the function of hsa-let-7b-5p.

**Results:**

Based on crosstalk genes in the KEGG, PPI network, and WGCNA analyses, PLK1, CCNA2, cyclin B2 (CCNB2), and AURKA were screened as candidate diagnostic marker genes. The survival analysis revealed that high mRNA expression of PLK1, CCNA2, and AURKA was significantly associated with poor overall survival. Furthermore, hsa-let-7b-5p was identified as a core miRNA in the regulation of candidate genes involved in glioma development. We confirmed that hsa-let-7b-5p could inhibit the migration, invasion, and cell cycle of glioma cells.

**Conclusions:**

This study provides four potential biomarkers for the diagnosis of glioma, offers a potential explanation of its pathogenesis, and proposes hsa-let-7b-5p as a therapeutic target.

**Electronic supplementary material:**

The online version of this article (10.1186/s12967-019-1882-7) contains supplementary material, which is available to authorized users.

## Background

As the most common type of intracranial primary tumor, glioma has a poor prognosis due to limitations to its diagnosis [1]. Currently, glioma therapy is often composed of a combination of surgery, radiotherapy, and chemotherapy [2, 3]. Accurate and timely diagnosis of brain glioma and targeted treatment plans are the current challenges in the treatment of glioma. With the continuous expansion and deepening of clinical research, biological markers and molecular pathology can facilitate the diagnosis and treatment of various diseases [4]. Given the limitations in the diagnosis of glioma, the pathological tumor types, tumor biological behaviors, prognosis, and postoperative clinical treatment of patients with glioma remain unknown. Therefore, the study and discovery of glioma biomarkers can provide important references for the accurate diagnosis and treatment of glioma.

Previous studies on glioma have revealed many molecular markers, such as IDH, 1p19q, MGMT, sarcomarcoma proto oncogene B, telomerase reverse transcriptase tert, and epidermal growth factor receptor [5–7]. Among these molecular markers, IDH1, 1p19q, and MGMT methylation are closely associated with the prognosis and chemotherapy sensitivity of glioma patients [8–11]. However, these markers have limited sensitivity and accuracy [12]. Therefore, specific and novel early diagnostic markers are necessary to understand the pathogenesis of glioma.

At present, the study of gene expression and mutation in glioma has become an essential way to explore the development, progression, and prognosis of glioma. Thus, the identification of potential therapeutic targets for the treatment of glioma is desirable. A large number of abnormally expressed miRNAs can be found in tumor tissues or cells. MiRNAs are involved in the posttranscriptional regulation of genes, thus inhibiting the expression of target genes and indirectly affecting various molecules in the cell signaling pathway, leading to abnormal tumor cell survival pathways and tumor cell death.

High-throughput sequencing and bioinformatics analysis are important tools for cancer research, including early diagnosis of cancer, grading of cancer, and prognosis prediction.

In the present study, a microarray dataset of normal brain and glioma tissues was downloaded from the TCGA database, and differentially expressed genes were analyzed using edgeR [13, 14]. Bioinformatics analysis of DEGs was performed to identify potential genes involved in glioma development. Then, GO analysis, including of the biological processes (BPs), molecular functions (MFs), cellular components (CCs), and KEGG pathways, of the DEGs was performed to reveal the differential functions and pathways of glioma compared with normal tissues [15]. Moreover, we established the PPI network of the DEGs and WGCNA coexpression network and selected hub genes with a high degree of connectivity [16–18]. The results showed that hsa-let-7b-5p could regulate target genes, including cell cycle protein A2 (CCNA2), CCNB2, PLK1, and AURKA, and further affect the progress of glioma. This study may provide novel insights into the molecular and biological processes and targets of glioma.

## Methods

### Reagents

The hsa-let-7b-5p mimic was purchased from Biomics Bitotechnologies Co., Ltd. (Jiangsu, China). Negative control nucleotides were purchased from GenePharm, Inc. (Sunnyvale, CA, USA). The reagents used for cell culture were purchased from Gibco-BRL (Carlsbad, CA, USA).

### TCGA data information

The glioma data set was downloaded from the Broad GDAC Firehouse website (http://gdac.broadinstitute.org/). The database contained 5 normal samples and 696 glioma samples. The edgeR packages of Bioconductor analysis tools were applied to detect the DEGs, using FDR < 0.05 and |logFC| ≥ 2 as cut-off criteria. A total of 1626 DEGs were calculated, including 601 upregulated genes and 1025 downregulated genes.

### GO function and KEGG pathway enrichment analysis of DEGs

Candidate DEG functions were analyzed using the Database for Annotation, Visualization, and Integrated Discovery (DAVID, https://david.ncifcrf.gov/) and the ClueGO plug-in of Cytoscape. KEGG pathway enrichment analysis was carried out and visualized using ClueGO and CluePedia with P < 0.05 as the cut-off criterion.

### Integration of the protein–protein interaction (PPI) network

A DEG-encoded PPI network was developed by using the online database STRING (http://string-db.org). A protein interaction relationship network table was downloaded and visualized using Cytoscape software.

### Module selection from the PPI network

The CentiScape plug-in was used to calculate the node degree. The molecular complex detection (MCODE) plug-in was employed to find clusters in the whole PPI network with a node score cut-off = 0.2, k-core = 2, and maximum depth = 100 as cut-off criteria. Proteins in the central nodes might have important physiological regulatory functions and might be key candidate genes. Subsequently, the genes in the most significant module were extracted and subjected to GO function and KEGG pathway enrichment analysis at a significance of P < 0.05.

### WGCNA

WGCNA was used for the scale-free network topology analysis of microarray expression data of glioma samples. Standard WGCNA parameters were used for analysis, with the exceptions of soft-thresholding power and the deep split. By using WGCNA, a coexpression module of genes related to sample characteristics was quickly extracted from the glioma data for subsequent analysis. In brief, genes with expression correlation were clustered into a module by calculating the expression correlation between genes. To broadly identify the coexpressed genes, all genes were used for analysis. The purpose of the module cluster analysis was to integrate the genes obtained by WGCNA with the key genes previously analyzed to further narrow the scope of key genes. The black cluster module is the most suitable for narrowing the range of key genes. A bridge between sample characteristics and changes in gene expression was constructed. A total of 109 genes in the black model were extracted and further subjected to Venn analysis with crosstalk genes of KEGG pathways and hub genes of PPI. The file content interaction relationships of 109 genes was exported and visualized by Cytoscape.

### miRNA–mRNA regulatory interaction networks

A miRNA–mRNA regulatory network was constructed using CyTargetLinker of Cytoscape. Briefly, a file was created containing the name of the miRNA and the name of the key genes and was imported into CytoScape. Then, an experimentally validated microRNA–target interaction relationship was downloaded from miRTarBase (http://projects.bigcat.unimaas.nl/cytargetlinker/regins/) with 410,602 interactions, 2645 microRNAs, and 14,797 target genes. Then, the downloaded miRNA database was imported into the extend network of CyTargetLinker. According to the constructed miRNA–mRNA network, large amounts of prediction information can be acquired when predicting miRNA target genes. Here, miRNAs that could bind to most target genes were selected as key miRNAs. Other redundant information was further removed to construct the final miRNA–mRNA network.

### Comparison of the expression level and correlation coefficient of candidate genes

The mRNA expression levels of PLK1, CCNA2, CCNB2, and AURKA were analyzed using the GEPIA (http://gepia.cancer-pku.cn/index.html) web server. A boxplot was created to visualize the expression level.

### Expression level of candidate genes in normal brain tissues and glioma specimens

The protein expression levels of PLK1, CCNA2, CCNB2, and AURKA in normal brain tissues and glioma specimens were investigated, and images were obtained from the Human Protein Atlas (http://www.proteinatlas.org) online database.

### Survival analysis of PLK1, CCNA2 and AURKA

The clinical data were downloaded from the TCGA database. Cases were divided into two groups according to gene expression. Survival analyses were performed using GraphPad Prism 6.

### Cell culture and transfection

U118MG cells were cultured in Dulbecco’s Modified Eagle’s Medium supplemented with 10% fetal bovine serum, penicillin (100 IU/mL), and streptomycin (0.1 mg/mL) in a humidified incubator at 37 °C in a 5% CO_2_ atmosphere.

Cells were plated in 6-well plates (3 × 10^5^ cells per plate) overnight prior to transfection. Transfection was performed using Lipofectamine 2000 reagent (Invitrogen, Carlsbad, USA) in accordance with the manufacturer’s protocol.

### RNA isolation and quantitative real-time polymerase chain reaction analysis

At 48 h after transfection with hsa-let-7b-5p mimic or negative control nucleotides, cells were collected and washed with PBS. Then, total RNA was isolated using the RNAprep pure cell/Bacteria kit (Tiangen Biotech Co., Ltd, Beijing, China) in accordance with the manufacturer’s instructions.

For miRNA expression detection, miRNA was reverse transcribed with miScript II RT Kit (Qiagen). The relative expression levels of hsa-let-7b-5p were determined with the miScript SYBR Green PCR Kit (Qiagen) using a QuantStudio TM6 Flex real-time PCR system (Life Technologies), and hsa-let-7b-5p (MS00003122). RNU6 (MS00029204) was used as an internal control miRNA.

For mRNA expression detection, the GoScript™ Reverse Transcription System (Promega Corporation, Madison, USA) was used to reverse transcribe RNA templates, and the relative expression levels of mRNAs were determined with GoTaq qPCR Master mix (Promega Corporation) using a QuantStudio TM6 Flex real-time PCR system. GAPDH was used as an endogenous control for mRNA expression. The primer sequences used in the current study are shown in Additional file 1: Table S1.

A comparative threshold cycle method (2^−ΔΔCT^) was used to calculate the relative expression of miRNAs and mRNAs.

### Wound healing assay

Wound healing (scratch) assays were performed to detect cell migration. U118MG cells were seeded in 6-well plates and incubated to generate confluent cultures. At 24 h after transfection with hsa-let-7b-5p mimic or negative control nucleotides, scratches were made in the cell monolayer using a 200-μL sterile pipette tip. Then, the cells were washed with PBS buffer. Images of cell migration at the edge of the scratch were obtained at 0, 24, and 48 h.

### Cell invasion assay

The invasion assay was carried out with a transwell chamber inserted with a PET filter membrane (8 μM pores, Corning, America) in 24-well plates (Corning, America). The top side of the filter was coated with Matrigel. At 24 h after transfection with hsa-let-7b-5p mimic or negative control nucleotides, the cells were collected and resuspended in serum-free medium (1 × 10^5^/mL). Then, 200 μL was added to the top well. The lower chamber was filled with 600 μL of medium containing 10% fetal bovine serum. After incubation at 37 °C for 24 h, the cells that crossed to the underside of the PET filter membrane were fixed with cold methanol for 30 min, stained with 0.1% crystal violet, and then counted under a microscope.

### Flow cytometry analysis

Flow cytometry analysis was performed using a BD LSRFortessa flow cytometer (BD Biosciences, San Jose, CA, USA) to determine the distribution of cells in the cell cycle and the proportion of apoptotic cells at 48 h after transfection with hsa-let-7b-5p mimic or negative control nucleotides. In brief, cells were collected and then fixed in 70% ethanol at 4 °C for 2 h. Then, the cells were treated with a 500 μL PI/RNase a working dyeing solution for 1 h at room temperature. Detailed information can be found in the cell cycle detection kit protocol (KeyGEN biotech, Nanjing, China). For examination of apoptosis, cells were stained using an Annexin V-FITC/PI apoptosis detection kit in accordance with the manufacturer’s protocol. All samples were analyzed with the FACScalibur flow cytometer, and data were analyzed with Flowjo 7.6.1 software.

### Statistical analysis

Statistical analyses were performed using GraphPad Prism 6. Statistically significant differences were calculated using the Student’s t-test, Pearson’s correlation, and Kaplan–Meier analysis, as appropriate. Statistical significance was considered at P < 0.05. For all data, *P < 0.05, **P < 0.01, ***P < 0.001.

## Results

### Identification of DEGs based on TCGA data in glioma

**Fig. 1:**
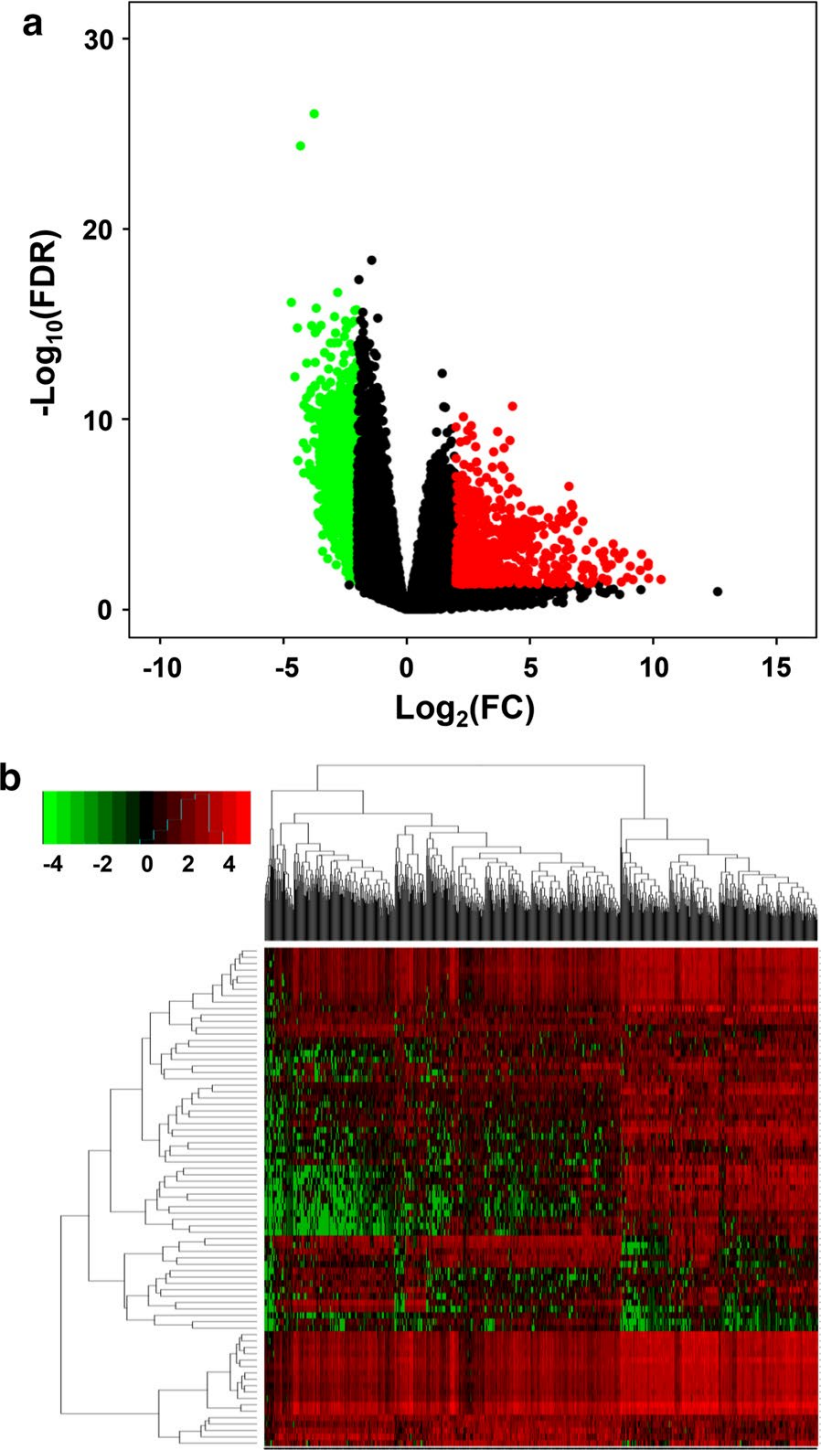
**a** Volcano plot of the genes between normal brain and glioma samples. Red dots indicate upregulated genes, and green dots indicate downregulated genes. Black dots show the genes with expression of |log_2_FC| < 2. The Y axis represents an FDR, and the X axis represents the value of log_2_FC. **b** Hierarchical clustering based on the expression profiles of significantly differentially expressed genes (DEGs)

A total of 17,746 genes from 5 normal samples and 696 glioma samples were obtained. By using edgeR, the expression level of each gene was log_2_ transformed. Following the calculation criteria, 1626 DEGs (601 upregulated and 1025 downregulated) were identified (Additional file 1: Table S2). As shown in the volcano plot, red dots indicate upregulated genes, and green dots indicate downregulated genes. Black dots show genes with expression of |log_2_FC| < 2 (Fig. 1a). We performed hierarchical cluster analysis to obtain an overview of the expression profile for the DEGs in the normal and glioma cases (Fig. 1b).

### GO function and KEGG pathway enrichment analysis and crosstalk gene screening

**Fig. 2:**
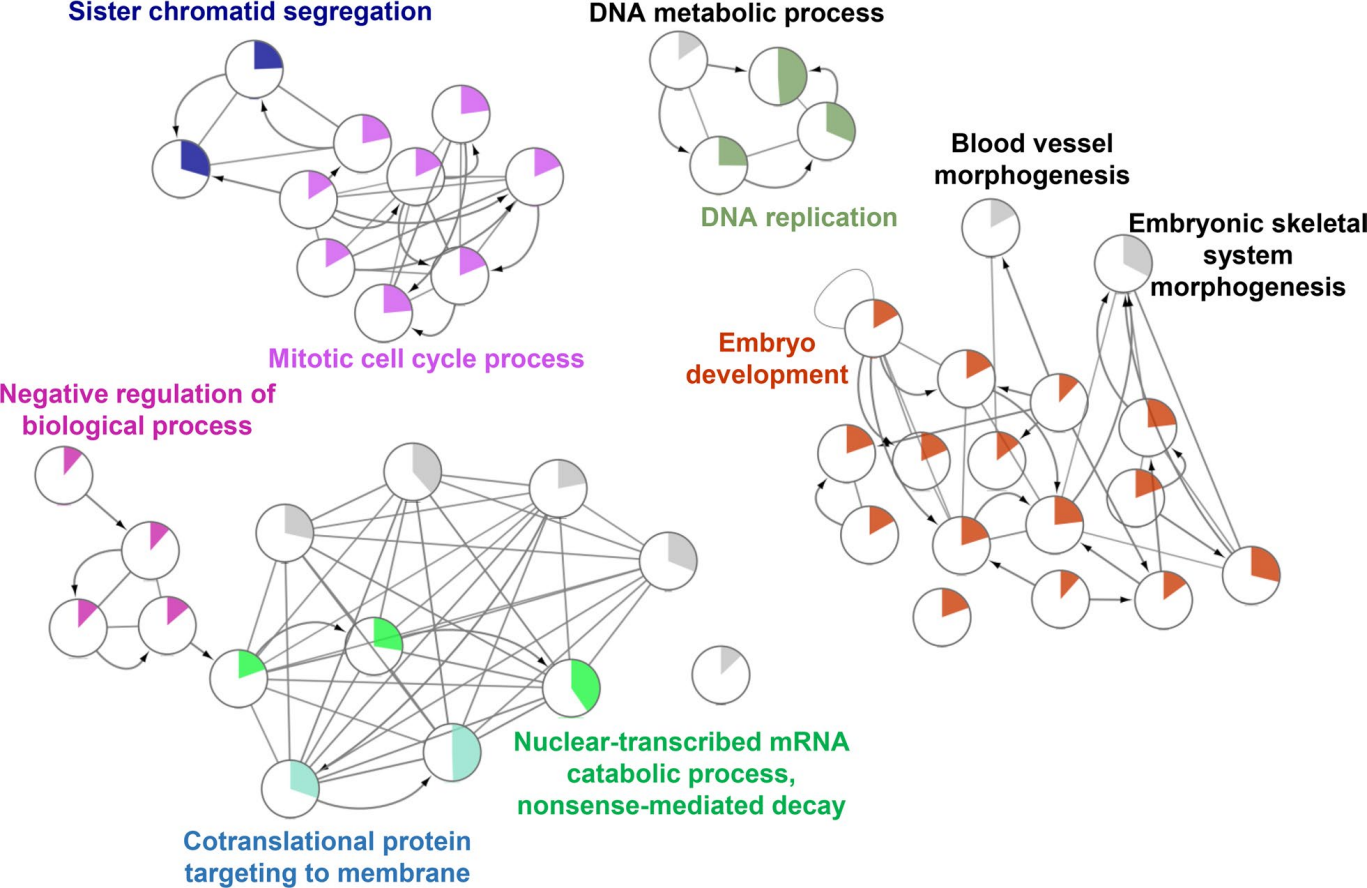
GO functional enrichment analysis of upregulated DEGs

For a more in-depth understanding of the selected DEGs, GO functional enrichment analysis was applied using DAVID [19, 20], and KEGG pathway enrichment analysis was performed using ClueGo of Cytoscape [21, 22]. The upregulated DEGs were particularly enriched in cell division, mitotic nuclear division, sister chromatid cohesion, and chromosome segregation (Fig. 2). Moreover, the downregulated genes were mainly involved in chemical synaptic transmission, neurotransmitter secretion, the G-protein coupled receptor signaling pathway, coupling to cyclic nucleotide secondary messenger, and the regulation of ion transmembrane transport. The top five GO pathways for the upregulated and downregulated DEGs are shown in Additional file 1: Table S3.

**Fig. 3:**
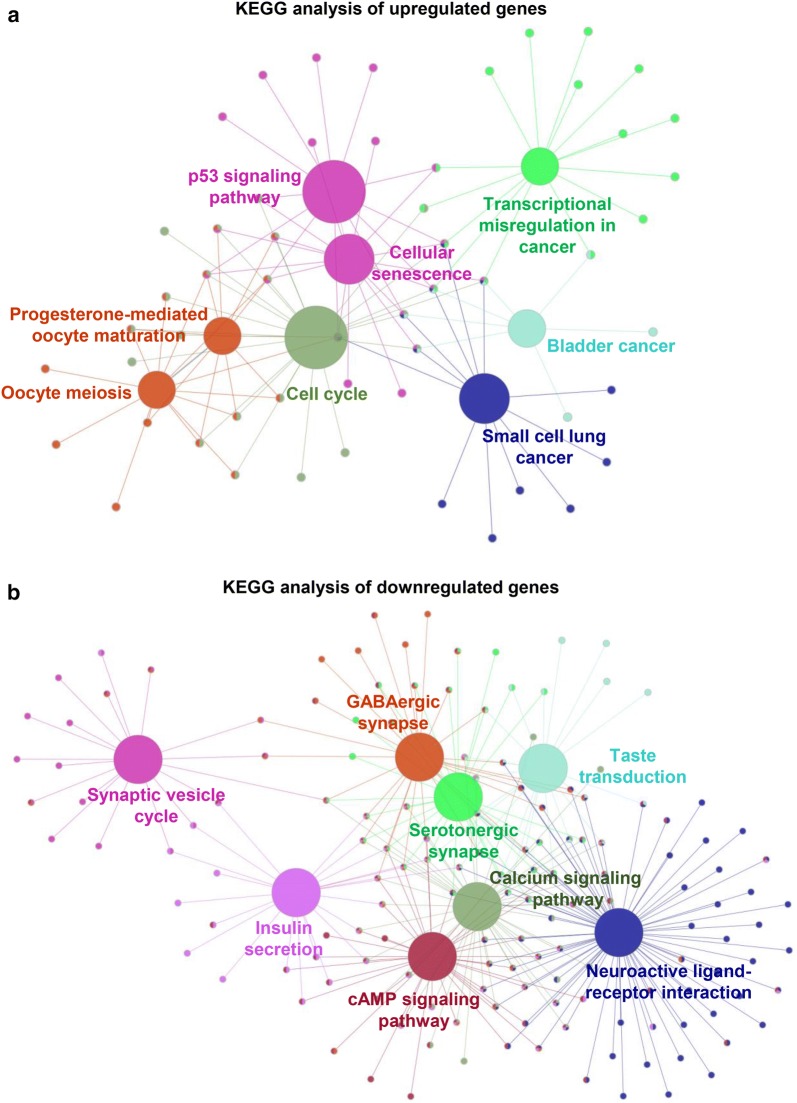
**a** KEGG pathway enrichment analysis of upregulated genes. **b** KEGG pathway enrichment analysis of downregulated genes

Based on the KEGG pathway enrichment analysis, the upregulated DEGs were enriched in the p53 signaling pathway, cellular senescence, transcriptional misregulation in cancer, progesterone-mediated oocyte maturation, and the cell cycle (Fig. 3a). The downregulated DEGs were associated with the synaptic vesicle cycle, GABAergic synapse, calcium signaling pathway, cAMP signaling pathway, and neuroactive ligand–receptor interactions (Fig. 3b). The top KEGG pathways for the upregulated and downregulated DEGs are shown in Additional file 1: Table S4. Furthermore, the relationship between genes and pathways could be clearly observed in pathways, and all the crosstalk genes are shown in Additional file 1: Table S5. Crosstalk genes are associated with more than two pathways and serve as a bridge. Thus, they warrant further study.

### Key candidate genes and pathway identification with DEG PPI and modular analyses

PPI relationships of the 1626 DEGs were established using STRING. A combined score of > 0.4 was selected in the PPI networks. Finally, 10,806 PPI relationships and 1310 nodes were obtained. The degree of connectivity of the DEGs was calculated using the CentiScape 2.2 plug-in module of Cytoscape. DEGs with degree of connectivity > 100 were selected as the hub genes, which possibly play an important role in glioma progression and can be used as diagnostic markers. The hub genes, of which there were > 100 in the PPI network according to their degrees, are shown in Additional file 1: Table S6.

**Fig. 4:**
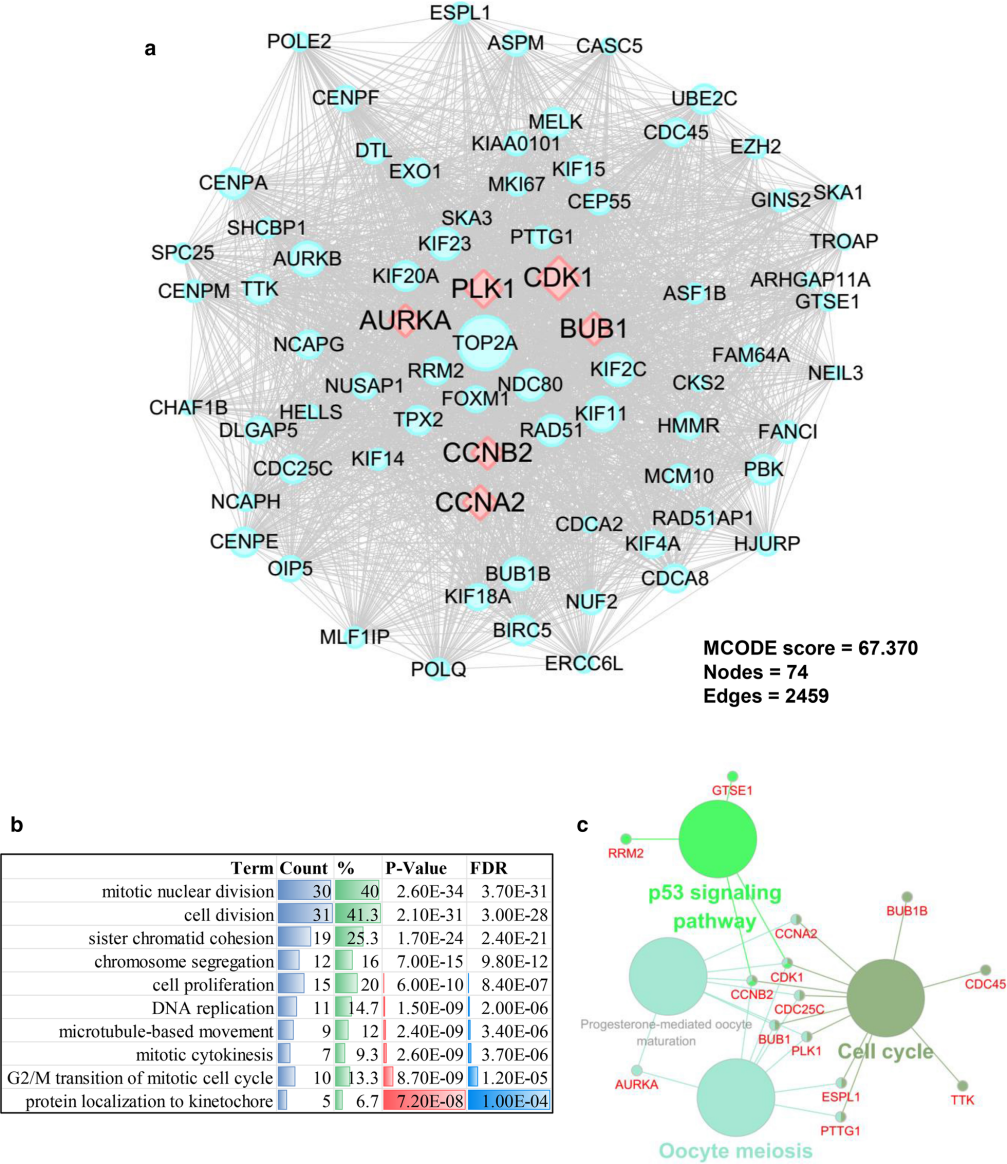
Protein–protein interaction network of the most significant module. **a** Most significant module and hub genes of the PPI network. **b** Enriched biological processes in the most significant DEG module. **c** Enriched pathways of the most significant module

The most significant submodules of the DEGs were selected using the MCODE plug-in module to understand further the biological significance of the PPI network. As shown in Fig. 4a, the most significant module (MCODE score = 67.370) was constructed with 74 nodes and 2459 edges (Fig. 4a). Further GO pathway enrichment analysis of the biological processes showed that the genes in the most significant module were mainly associated with mitotic nuclear division, cell division, sister chromatid cohesion, chromosome segregation, cell proliferation, and DNA replication (Fig. 4b). Cell component analysis indicated that the genes were strongly enriched in the nucleoplasm, chromosome, centromeric region, condensed chromosome kinetochore, spindle, and kinetochore. Molecular function analysis showed that the genes were mainly involved in protein binding, ATP binding, microtubule binding, microtubule motor activity, and chromatin binding. KEGG analysis showed that the genes were mainly enriched in the p53 signaling pathway, cell cycle, oocyte meiosis, and progesterone-mediated oocyte maturation (Fig. 4c).

### Gene–network modules identified by WGCNAs

**Fig. 5:**
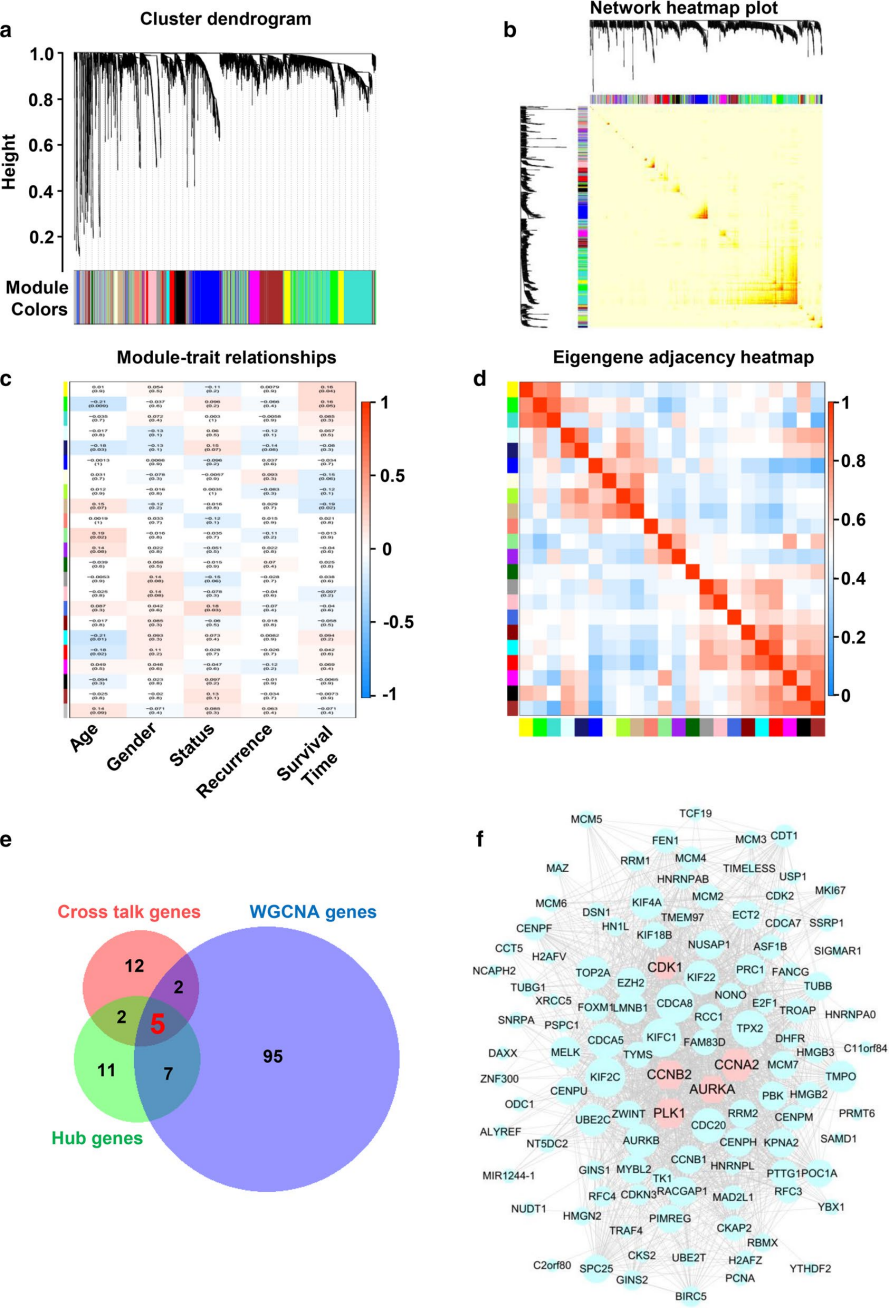
**a** WGCNA dendrogram indicating the expression of different gene modules in all glioma samples. **b** Network heatmap plot to visualize the genetic correlation within the modules. **c** Module-sample feature correlation analysis. Sample features include age, gender, status, recurrence, and survival time. **d** Correlation analysis between 23 coexpression modules. **e** Venn analysis of the crosstalk genes of pathways and hub genes in the PPI network and coexpression genes in WGCNA. Five candidate genes were calculated, including CDK1, PLK1, CCNA2, CCNB2, and AURKA. **f** Coexpression network of 105 WGCNA genes. Five candidate genes are displayed in pink

Gene coexpression networks were collected by obtaining pathological information and relative gene expression. The experimental results demonstrated that all genes were clustered into 34 modules (Fig. 5a, b). Different gene coexpression modules and the patient’s age, sex, survival status, recurrence, and survival time were further selected to draw a clustering heat map (Fig. 5c). To further narrowing the range of key genes, the black module genes were extracted and performed Venn analysis to select hub key genes in glioma. Finally, the genes CDK1, PLK1, CCNA2, CCNB2, and AURKA were selected. Hub gene network analysis of the black module was performed, and those five candidate genes are presented in Fig. 5f (pink nodes). Then, the key controlling (candidate) genes in the modular network were identified.

### hsa-let-7b-5p regulated the biomolecular network

**Fig. 6:**
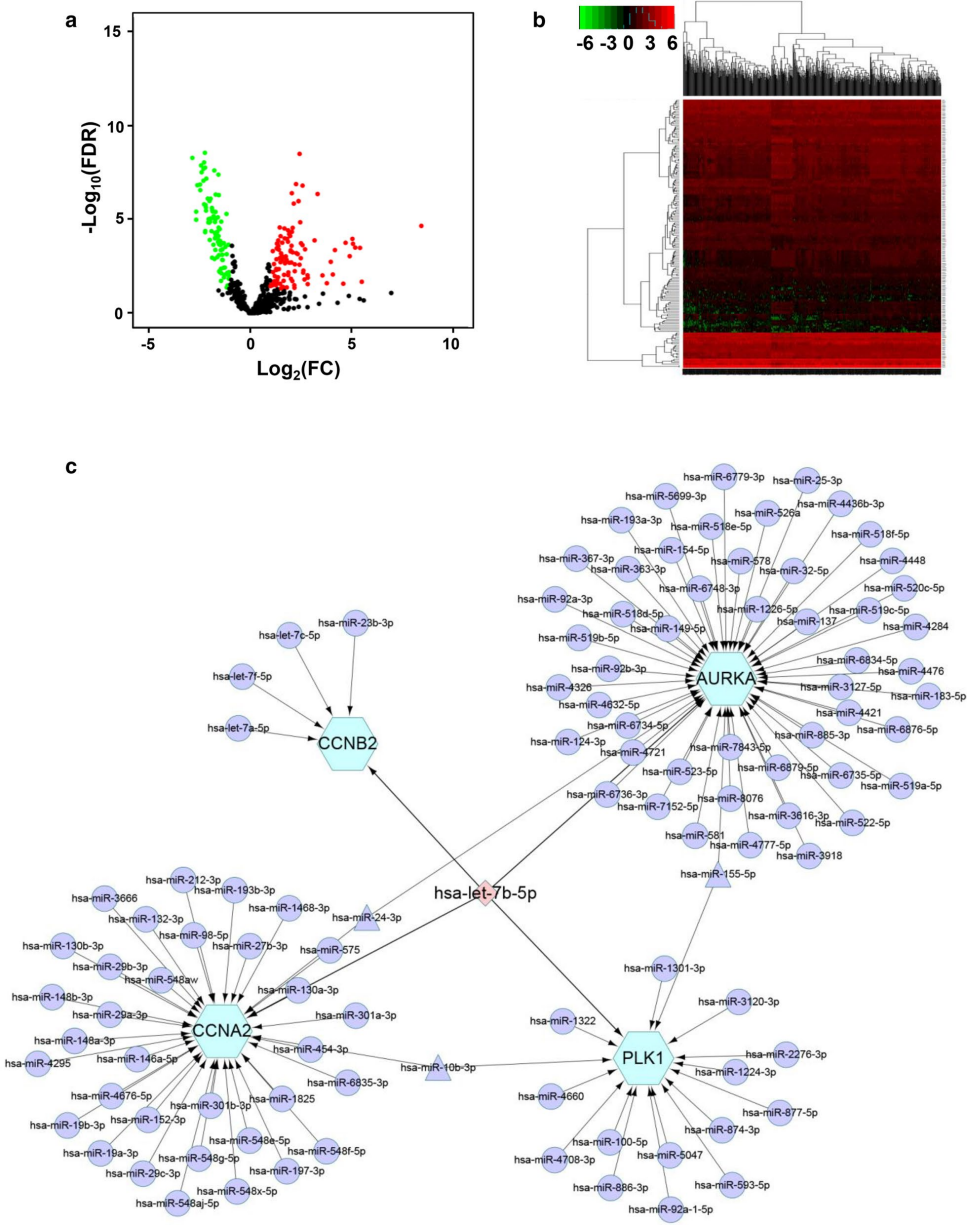
**a** Volcano plot of the miRNAs between normal brain and glioma samples. Red dots indicate upregulated genes, and green dots indicate downregulated genes. Black dots show the genes with expression of |log_2_FC| < 1. The Y axis represents an FDR, and the X axis represents the value of log_2_FC. **b** Hierarchical clustering based on the expression profiles of significantly differentially expressed miRNAs. **c** miRNA–mRNA regulatory interaction networks. The results show that hsa-let-7b-5p targets PLK1, CCNA2, CCNB2, and AURKA directly

Target microRNAs were searched using Cytoscape software to analyze the interplay between CDK1, PLK1, CCNA2, CCNB2, and AURKA and microRNAs. miRNA expression data were downloaded from the TCGA database. The expression level of each miRNA was calculated and log2 transformed using DESeq R. A total of 173 aberrantly expressed miRNAs (84 upregulated and 89 downregulated) in glioma tissues compared with normal brain tissues were achieved (Additional file 1: Table S7). In the volcano plot, red dots indicate upregulated genes, and green dots indicate downregulated genes. Black dots show genes with an expression of |log_2_FC| < 1 (Fig. 6a). Hierarchical cluster analysis was further performed to obtain an overview of the expression profile for differentially expressed miRNAs in normal and glioma cases (Fig. 6b). Using the CyTargetLinker plug-in of Cytoscape, we constructed the miRNA–mRNA expression regulatory network. The miRNA data were downloaded from miRTarBase (http://mirtarbase.mbc.nctu.edu.tw/), a database that curates experimentally validated microRNA–target interactions. The mirtarbase-hsa-6.1 database was selected, including 2645 microRNAs, 14,797 target genes, and 410,602 interactions. The final regulatory relationship is shown in Fig. 6c, and the results showed that hsa-let-7b-5p could modulate the expression of CCNB2, CCNA2, PLK1, and AURKA simultaneously (Fig. 6c).

### Validation of the DEGs in the human atlas dataset

**Fig. 7:**
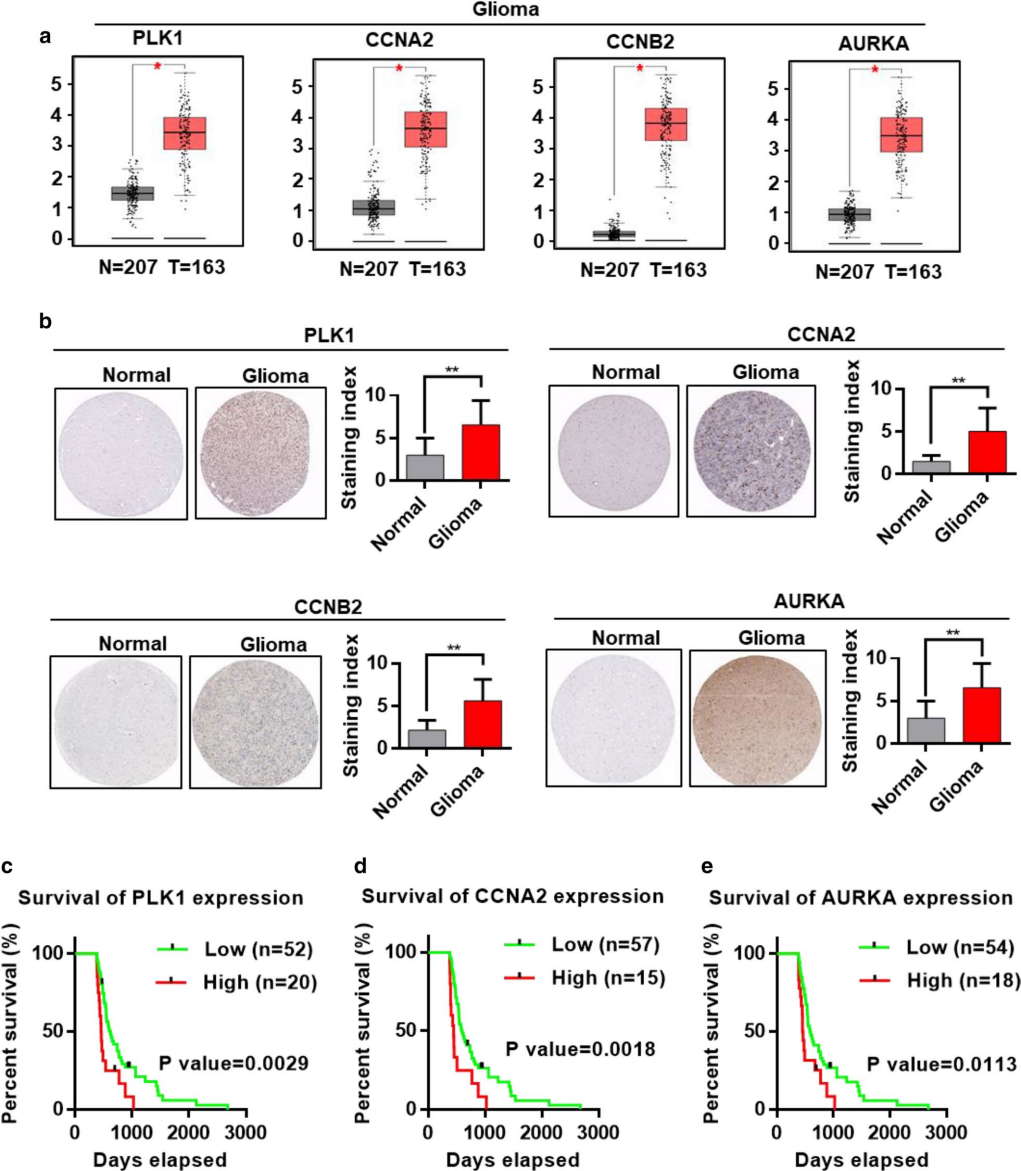
**a** Expression levels of PLK1, CCNA2, CCNB2, and AURKA in normal and glioma samples. **b** PLK1, CCNA2, CCNB2, and AURKA expression levels and staining index in normal brain tissues and glioma cancer specimens. Images were obtained from the Human Protein Atlas (http://www.proteinatlas.org) online database. **c** Survival curve between groups with low and high PLK1 expression. The red line represents cases with high PLK1 expression, and the green line represents cases with low PLK1 expression. **d** Survival curve between groups with low and high CCNA2 expression. The red line represents cases with high CCNA2 expression, and the green line represents cases with low CCNA2 expression. **e** Survival curve between groups with low and high AURKA expression groups. The red line represents cases with high AURKA expression, and the green line represents cases with low AURKA expression. The X axis indicates overall survival time (days), and the Y axis indicates the present survival (%). The clinical data were downloaded from TCGA

The mRNA expression levels of PLK1, CCNA2, CCNB2, and AURKA in normal brain tissues and glioma cancer specimens were investigated by GEPIA (Fig. 7a). Immunohistochemical and staining index analyses showed that the protein expression levels of PLK1, CCNA2, CCNB2, and AURKA were higher in glioma cancer tissues than in nontumor tissues. Images were obtained from the Human Protein Atlas (http://www.proteinatlas.org) online database (Fig. 7b). Interestingly, glioma cases with high PLK1 expression demonstrated a poorer survival compared with those with low PLK1 expression (P = 0.0029, Fig. 7c). Similarly, high expression of CCNA2 and AURKA was associated with a worse overall survival (OS) of glioma patients (Fig. 7d, e). The expression level of CCNB2 was not associated with the OS of patients with glioma.

### hsa-let-7b-5p negatively regulated PLK1, CCNA2, CCNB2, and AURKA; inhibited the migration and invasion of U118MG cells; and induced apoptosis and altered cell cycle dynamics of U118MG cells

**Fig. 8:**
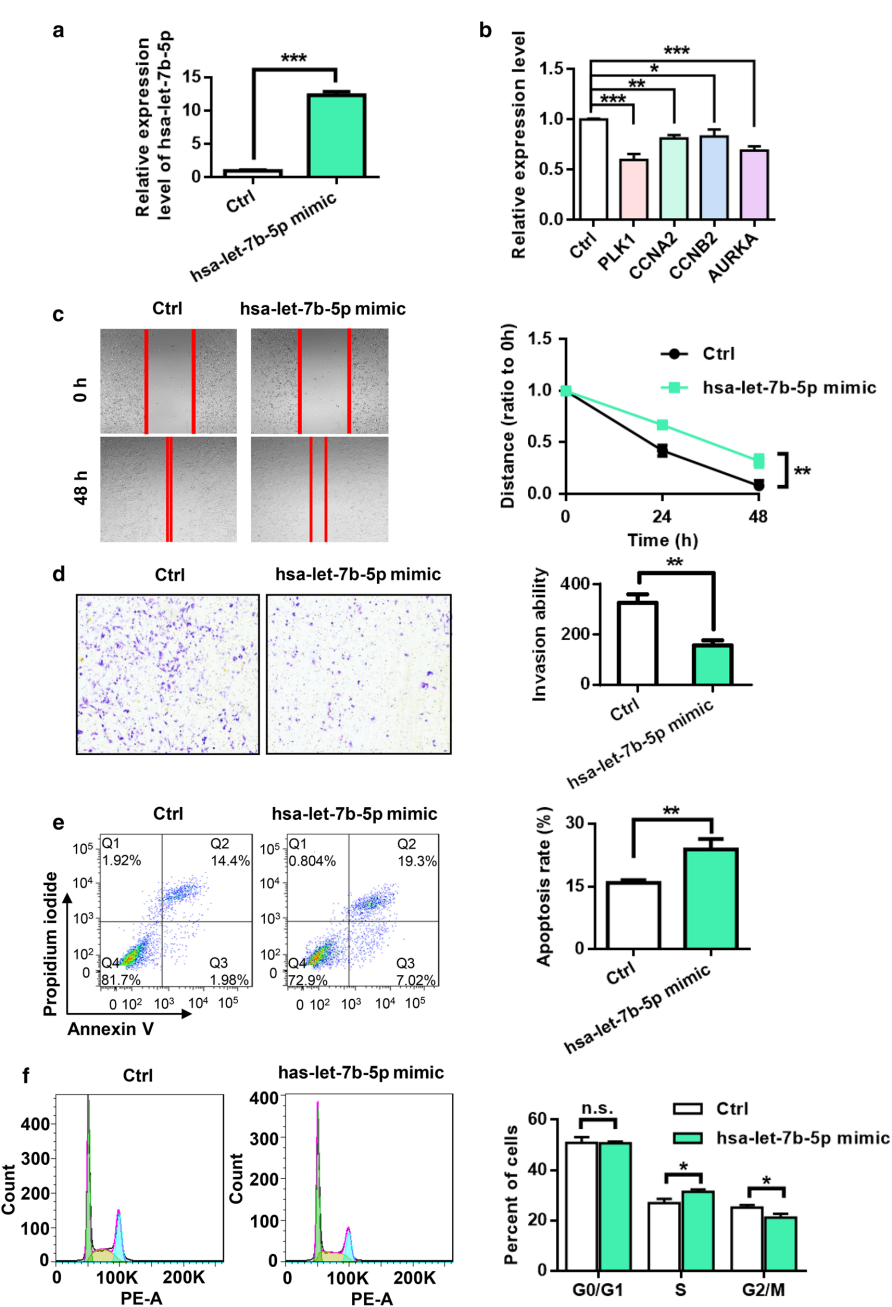
PLK1, CCNA2, CCNB2 and AURKA were targets of hsa-let-7b-5p and hsa-let-7b-5p could inhibit migration and invasion, induce apoptosis and alter the cell cycle dynamics of U118MG cells. **a** Expression of hsa-let-7b-5p was measured using real-time PCR in U118MG cells after transfection for 48 h. **b** Relative expression level of PLK1, CCNA2, CCNB2, and AURKA after transfection of hsa-let-7b-5p in U118MG cells. **c** hsa-let-7b-5p treatment could significantly inhibit the migration of U118MG cells. Scale bars, 100 μm. **d** hsa-let-7b-5p treatment could significantly inhibit the invasion of U118MG cells. Scale bars, 50 μm. **e** hsa-let-7b-5p could significantly induce the apoptosis of U118MG cells. **f** hsa-let-7b-5p could effectively inhibit the cycle progression of U118MG cells

We transfected U118MG cells with negative control nucleotides or hsa-let-7b-5p mimic to study the role of hsa-let-7b-5p in regulating PLK1, CCNA2, CCNB2, and AURKA. Forty-eight hours after transfection with control nucleotides or mimic, the expression levels of hsa-let-b-5p, PLK1, CCNA2, CCNB2, and AURKA were determined by qRT-PCR analysis. The level of hsa-let-7b-5p increased in the cells transfected with hsa-let-7b-5p mimic (12.30 ± 0.55) compared with that in the negative control group (0.98 ± 0.13) (*P* < 0.001) (Fig. 8a). The mRNA expression levels of PLK1, CCNA2, CCNB2, and AURKA were significantly lower in the cells transfected with hsa-let-7b-5p mimic (0.60 ± 0.06, 0.81 ± 0.03, 0.83 ± 0.07, and 0.69 ± 0.04, respectively) than in the negative control group (1.00 ± 0.01) (*P *< 0.001, *P *< 0.01, *P *< 0.05, and *P *< 0.001, respectively). These results indicated that hsa-let-7b-5p negatively regulated the expression of PLK1, CCNA2, CCNB2, and AURKA (Fig. 8b).

To assess the effect of hsa-let-7b-5p on the migration of U118MG cells in vitro, we transfected the U118MG cells with negative control nucleotides or hsa-let-7b-5p mimic. After 0, 24, and 48 h, images were obtained of cell migration at the edge of the scratch (Fig. 8c). After 24 and 48 h, the wound gap was markedly wider in the hsa-let-7b-5p mimic group than in the negative control group. This result showed that hsa-let-7b-5p inhibited the motility of the U118MG cells in vitro. Then, we investigated the effect of hsa-let-7b-5p on the invasion of U118MG cells by using Matrigel-coated transwell chambers. The number of cells that crossed to the Matrigel-coated filter membrane was significantly reduced in the hsa-let-7b-5p mimic group compared to that in the negative control group after 24 h (Fig. 8d).

U118MG cells were transfected with negative control nucleotides or hsa-let-7b-5p mimic to investigate the role of hsa-let-7b-5p in apoptosis by flow cytometry analysis with an Annexin V-FITC/PI apoptosis detection kit. At 48 h posttransfection, cells were collected and analyzed in accordance with the manufacturer’s protocol. As shown in Fig. 8e, the total apoptosis rate of the U118MG cells transfected with hsa-let-7b-5p mimic (23.83% ± 2.51%) was significantly higher than that of the cells transfected with negative control nucleotides (15.84% ± 0.67%) (*P *< 0.05). The result indicated that overexpression of hsa-let-7b-5p increased the apoptosis of U118MG cells. Then, we determined the effect of hsa-let-7b-5p overexpression on the U118MG cell cycle. Compared with the control group, overexpression of hsa-let-7b-5p increased the proportion of cells in S phase and decreased the proportion of cells in G2 phase (Fig. 8f). The results showed that hsa-let-7b-5p could alter cell cycle dynamics in vitro.

## Discussion

Glioma is the most common primary intracranial tumor [23–25]. Radical surgery combined with radiotherapy and chemotherapy is the basic means to treat glioma, but the treatment effect is not optimistic due to its easy recurrence and high mortality [25]. With continuous research and improved understanding of glioma in recent years, various treatment methods, such as gene therapy, immunotherapy, and molecular targeted therapy, have been proposed [26–30]. However, a method to cure glioma is currently lacking. Early diagnosis of glioma and timely surgical treatment or intervention with medicine is an important means to prolong the life of patients with this disease [31, 32].

In this study, expression profile chip data of 696 glioma samples and five normal samples were downloaded from the TCGA database. A total of 1626 DEGs were calculated. GO function and KEGG pathway analyses were performed to acquire an in-depth understanding of these DEGs. The GO analysis showed that the upregulated DEGs were mainly involved in cell division, mitotic nuclear division, sister chromatid cohesion, embryonic skeletal system morphogenesis, anterior/posterior pattern specification, chromosome segregation, and cell proliferation. The downregulated genes were involved in chemical synaptic transmission, neurotransmitter secretion, G-protein coupled receptor signaling pathway, coupling to cyclic nucleotide second messenger, regulation of ion transmembrane transport, potassium ion transport, and calcium ion transmembrane transport. Furthermore, the KEGG pathways of the upregulated genes were included in the cell cycle, transcriptional misregulation in cancer, bladder cancer, small cell lung cancer, the p53 signaling pathway, and cellular senescence. Moreover, the downregulated DEGs were enriched in the calcium signaling pathway, serotonergic synapse, taste transduction, neuroactive ligand–receptor interaction, synaptic vesicle cycle, and retrograde endocannabinoid signaling.

Analysis of the most significant module of the PPI network showed that glioma was associated with the biological processes of mitotic nuclear division, cell division, sister chromatid cohesion, chromosome segregation, cell proliferation, DNA replication, and microtubule-based movement. KEGG pathway enrichment analysis for the most significant module was mainly involved in the cell cycle, oocyte meiosis, progesterone-mediated oocyte maturation, and the p53 signaling pathway.

Studies have shown that molecular target pathways, such as the growth factor pathway, Ras pathway, PI3K pathway, p53 pathway, and tumor metastasis invasion pathway, have great potential for the development of meaningful treatment strategies for glioma [33, 34]. The p53 pathway is one of the important pathways for the molecular pathogenesis of glioma. P53 can be divided into wild-type p53 and mutant p53. Wild-type p53 is an anti-oncogene that rapidly induces apoptosis in damaged cells, prevents cells with potentially cancerous potential, and exerts anticancer effects. Once wild-type p53 is mutated, it no longer has a tumor suppressing effect but can accelerate the proliferation and growth of cancer cells. When wild-type p53 is transformed to mutant p53, the mutant p53 acts as a proto-oncogene, which is present for a long time in tumor cells and eventually leads to tumorigenesis. The KEGG pathway analysis enriches the p53 pathway, which may be due to increased p53 mutations in gliomas (Additional file 1: Figures S1, S2).

Among these DEGs, five candidate genes with a high degree of connectivity and coexpression and crosstalk in pathways were selected, including CDK1, CCNA2, CCNB2, PLK1, and AURKA. miRNAs can change the expression of target proteins. MiRNAs have important clinical significance in the diagnosis of glioma, evaluation of the efficacy of chemotherapy drugs, anti-angiogenesis, treatment, and prognosis judgment. In the present study, the expression levels of CCAN2, CCNB2, PLK1, and AURKA in glioma were upregulated, and hsa-let-7b-5p binding sites were found in four gene sequences. Let-7b is a cancer suppressor gene that can inhibit the occurrence of cancer [35, 36].

As the most significant candidate gene, polo-like kinase 1 (PLK1), is a highly conserved serine/threonine protein kinase. PLK1 is highly expressed in most malignant tumor cells and is closely related to the occurrence and development of tumors [37–40]. PLK1 is mainly expressed in the late G2 phase and M phase of cell mitosis and plays a crucial role in mitosis, such as mitotic initiation, centrosome maturation, spindle assembly, chromosome segregation, and cytokinesis. Clinical data show that inhibition of PLK1 can prevent the proliferation and promote the apoptosis of tumor cells. Therefore, PLK1 is an effective target for the treatment of gliomas. Cell cycle protein A2 (CCNA2) is encoded by the CCNA2 gene in humans. CCNA2 is expressed in cell division and interacts with CDK kinase to regulate cell cycle progression [41]. The CCNA2–CDK complex may promote tumorigenesis by phosphorylation of cancer proteins or tumor suppressor genes, such as p53. CCNA2 is highly expressed in a variety of tumor types, such as lung cancer, breast cancer, cervical cancer, and liver cancer [42–45]. In normal tissues, CCNA2 is expressed at low levels or is not expressed. Some studies have confirmed that CCNA2 has oncogene activity, and its high expression or overexpression is closely related to the malignant transformation of tumor cells. Therefore, CCNA2 may be a potential diagnostic marker and an important drug target for glioma. CCNB2 plays a role in the cell cycle. CCNB2 is highly expressed in a variety of human tumor tissues and peripheral blood, and it is associated with the clinical stage and metastasis of the tumor [46–48]. CCNB2 overexpression is negatively correlated with the prognosis of breast cancer patients and is an independent prognostic marker. In addition, CCNB2 mRNA is overexpressed in the tumor tissue of patients with lung adenocarcinoma, and its expression is closely related to the overall disease survival rate. High expression of CCNB2 in tumor tissues is closely related to the poor prognosis of gliomas. AURKA, a member of the Aurora kinase family, is an oncogene that plays an important role in cancer stem cell development, epithelial–mesenchymal transition, and distant metastasis [49–51]. AURKA protein kinase plays an important role in the development of malignant tumors. AURKA has been highly expressed in various malignant tumors, such as esophageal cancer, laryngeal cancer, liver cancer, and ovarian cancer [52–54]. AURKA may participate in the formation of tumors through two functions. One function is the suppression of cytokinesis during mitosis, which leads to an unstable tumor genome. The other function is the inhibition of cell cycle checkpoints, helping cells with unstable genomes continue to replicate and to enter mitosis, leading to the malignant proliferation of cells. AURKA protein kinase can also promote the formation of blood vessel mimicry in triple-negative breast cancer stem cells, and inhibiting its expression can weaken the formation of blood vessel mimicry capacity.

In the present study, the RT-PCR assay confirmed that CCNA2, CCNB2, PLK1, and AURKA are targets of hsa-let-7b-5p. Hsa-let-7b-5p could inhibit the migration, invasion, and cell cycle of glioma cells.

## Conclusions

In summary, PLK1, CCNA2, CCNB2, and AURKA were screened as candidate diagnostic marker genes of glioma, and hsa-let-7b-5p could inhibit the invasion and metastasis of glioma cells. The underlying mechanism may be achieved by targeting the downregulation of PLK1, CCNA2, CCNB2, and AURKA protein expression. This discovery is expected to provide new therapeutic targets and biomarkers for the future treatment of gliomas.

## Additional file

**Additional file 1: Figure S1.** P53 mutation in Glioma patients. **Figure S2.** Mutation site information of p53 in Glioma patients. **Table S1.** Primers used for quantitative real-time polymerase chain reaction. **Table S2.** Differentially expressed genes between normal brain and glioma samples. **Table S3.** Gene ontology enrichment analysis of upregulated and downregulated genes. **Table S4.** KEGG pathway enrichment analysis of upregulated and downregulated genes. **Table S5.** Cross-talk genes involved in different pathways. **Table S6.** Hub genes in the PPI network with degree more than 100. **Table S7.** Differentially expressed miRNAs between normal brain and glioma samples.

## Abbreviations

TCGA: The Cancer Genome Atlas
WGCNA: weighted correlation network analysis
DEGs: differentially expressed genes
GO: Gene Ontology
KEGG: Kyoto Encyclopedia of Genes and Genomes
BP: biological process
MF: molecular function
CC: cellular component
PPI: protein–protein interaction

## References

[1] Furnari FB, Fenton T, Bachoo RM, Mukasa A, Stommel JM, Stegh A (2007). Malignant astrocytic glioma: genetics, biology, and paths to treatment. Genes Dev.

[2] Minniti G, De Sanctis V, Muni R, Filippone F, Bozzao A, Valeriani M (2008). Radiotherapy plus concomitant and adjuvant temozolomide for glioblastoma in elderly patients. J Neurooncol.

[3] Stupp R, Mason WP, van den Bent MJ, Weller M, Fisher B, Taphoorn MJ (2005). Radiotherapy plus concomitant and adjuvant temozolomide for glioblastoma. N Engl J Med.

[4] Vecera M, Sana J, Lipina R, Smrcka M, Slaby O (2018). Long non-coding RNAs in gliomas: from molecular pathology to diagnostic biomarkers and therapeutic targets. Int J Mol Sci.

[5] Yan H, Parsons DW, Jin G, McLendon R, Rasheed BA, Yuan W (2009). IDH1 and IDH2 mutations in gliomas. N Engl J Med.

[6] Killela PJ, Pirozzi CJ, Healy P, Reitman ZJ, Lipp E, Rasheed BA (2014). Mutations in IDH1, IDH2, and in the TERT promoter define clinically distinct subgroups of adult malignant gliomas. Oncotarget.

[7] Hegi ME, Diserens AC, Gorlia T, Hamou MF, de Tribolet N, Weller M (2005). MGMT gene silencing and benefit from temozolomide in glioblastoma. N Engl J Med.

[8] Gao YF, Mao XY, Zhu T, Mao CX, Liu ZX, Wang ZB (2016). COL3A1 and SNAP91: novel glioblastoma markers with diagnostic and prognostic value. Oncotarget.

[9] Jung CS, Unterberg AW, Hartmann C (2011). Diagnostic markers for glioblastoma. Histol Histopathol.

[10] Donev K, Scheithauer BW, Rodriguez FJ, Jenkins S (2010). Expression of diagnostic neuronal markers and outcome in glioblastoma. Neuropathol Appl Neurobiol.

[11] Reddy SP, Britto R, Vinnakota K, Aparna H, Sreepathi HK, Thota B (2008). Novel glioblastoma markers with diagnostic and prognostic value identified through transcriptome analysis. Clin Cancer Res.

[12] Dunbar E, Yachnis AT (2010). Glioma diagnosis: immunohistochemistry and beyond. Adv Anat Pathol.

[13] Liu J, Lichtenberg T, Hoadley KA, Poisson LM, Lazar AJ, Cherniack AD (2018). An integrated TCGA pan-cancer clinical data resource to drive high-quality survival outcome analytics. Cell.

[14] Sanchez-Vega F, Mina M, Armenia J, Chatila WK, Luna A, La KC (2018). Oncogenic signaling pathways in The Cancer Genome Atlas. Cell.

[15] Zhang H, Liu T, Zhang Z, Payne SH, Zhang B, McDermott JE (2016). Integrated proteogenomic characterization of human high-grade serous ovarian cancer. Cell.

[16] Lin X, Li J, Zhao Q, Feng JR, Gao Q, Nie JY (2018). WGCNA reveals key roles of IL8 and MMP-9 in progression of involvement area in colon of patients with ulcerative colitis. Curr Med Sci.

[17] Deng J, Kong W, Mou X, Wang S, Zeng W (2018). Identifying novel candidate biomarkers of RCC based on WGCNA analysis. Pers Med.

[18] Langfelder P, Horvath S (2008). WGCNA: an R package for weighted correlation network analysis. BMC Bioinform.

[19] Mele M, Ferreira PG, Reverter F, DeLuca DS, Monlong J, Sammeth M (2015). Human genomics. The human transcriptome across tissues and individuals. Science..

[20] da Huang W, Sherman BT, Lempicki RA (2009). Systematic and integrative analysis of large gene lists using DAVID bioinformatics resources. Nat Protoc.

[21] Reimand J, Isserlin R, Voisin V, Kucera M, Tannus-Lopes C, Rostamianfar A (2019). Pathway enrichment analysis and visualization of omics data using g:Profiler, GSEA, Cytoscape and EnrichmentMap. Nat Protoc.

[22] Bindea G, Galon J, Mlecnik B (2013). CluePedia Cytoscape plugin: pathway insights using integrated experimental and in silico data. Bioinformatics.

[23] Lapointe S, Perry A, Butowski NA (2018). Primary brain tumours in adults. Lancet.

[24] Brandner S, Jaunmuktane Z (2018). Neurological update: gliomas and other primary brain tumours in adults. J Neurol.

[25] McNamara S (2012). Treatment of primary brain tumours in adults. Nurs Stand.

[26] Ryu CH, Park SH, Park SA, Kim SM, Lim JY, Jeong CH (2011). Gene therapy of intracranial glioma using interleukin 12-secreting human umbilical cord blood-derived mesenchymal stem cells. Hum Gene Ther.

[27] Kim SM, Lim JY, Park SI, Jeong CH, Oh JH, Jeong M (2008). Gene therapy using TRAIL-secreting human umbilical cord blood-derived mesenchymal stem cells against intracranial glioma. Cancer Res.

[28] Portnow J, Synold TW, Badie B, Tirughana R, Lacey SF, D’Apuzzo M (2017). Neural stem cell-based anticancer gene therapy: a first-in-human study in recurrent high-grade glioma patients. Clin Cancer Res.

[29] Dimitrov L, Hong CS, Yang C, Zhuang Z, Heiss JD (2015). New developments in the pathogenesis and therapeutic targeting of the IDH1 mutation in glioma. Int J Med Sci.

[30] Wang H, Xu T, Jiang Y, Xu H, Yan Y, Fu D (2015). The challenges and the promise of molecular targeted therapy in malignant gliomas. Neoplasia.

[31] Rossi LN, Pastorino G, Scotti G, Gazocchi M, Maninetti MM, Zanolini C (1994). Early diagnosis of optic glioma in children with neurofibromatosis type 1. Childs Nerv Syst.

[32] Baehring JM, Bi WL, Bannykh S, Piepmeier JM, Fulbright RK (2007). Diffusion MRI in the early diagnosis of malignant glioma. J Neurooncol.

[33] Xu LX, Li ZH, Tao YF, Li RH, Fang F, Zhao H (2014). Histone acetyltransferase inhibitor II induces apoptosis in glioma cell lines via the p53 signaling pathway. J Exp Clin Cancer Res.

[34] Breunig JJ, Levy R, Antonuk CD, Molina J, Dutra-Clarke M, Park H (2016). Ets factors regulate neural stem cell depletion and gliogenesis in Ras pathway glioma. Cell Rep.

[35] Vishnubalaji R, Hamam R, Abdulla MH, Mohammed MA, Kassem M, Al-Obeed O (2015). Genome-wide mRNA and miRNA expression profiling reveal multiple regulatory networks in colorectal cancer. Cell Death Dis.

[36] Xu H, Liu C, Zhang Y, Guo X, Liu Z, Luo Z (2014). Let-7b-5p regulates proliferation and apoptosis in multiple myeloma by targeting IGF1R. Acta Biochim Biophys Sin.

[37] Wu ZY, Wei N (2018). Knockdown of PLK1 inhibits invasion and promotes apoptosis in glioma cells through regulating autophagy. Eur Rev Med Pharmacol Sci.

[38] Cheng MW, Wang BC, Weng ZQ, Zhu XW (2012). Clinicopathological significance of Polo-like kinase 1 (PLK1) expression in human malignant glioma. Acta Histochem.

[39] Dalvi PS, Macheleidt IF, Lim SY, Meemboor S, Muller M, Eischeid-Scholz H (2019). LSD1 inhibition attenuates tumor growth by disrupting PLK1 mitotic pathway. Mol Cancer Res..

[40] Ramani P, Nash R, Sowa-Avugrah E, Rogers C (2015). High levels of polo-like kinase 1 and phosphorylated translationally controlled tumor protein indicate poor prognosis in neuroblastomas. J Neurooncol.

[41] Wen DY, Lin P, Pang YY, Chen G, He Y, Dang YW (2018). Expression of the long intergenic non-protein coding RNA 665 (LINC00665) gene and the cell cycle in hepatocellular carcinoma using The Cancer Genome Atlas, the Gene Expression Omnibus, and Quantitative Real-Time Polymerase Chain Reaction. Med Sci Monit.

[42] Li J, Ying Y, Xie H, Jin K, Yan H, Wang S (2018). Dual regulatory role of CCNA2 in modulating CDK6 and MET-mediated cell-cycle pathway and EMT progression is blocked by miR-381-3p in bladder cancer. FASEB J.

[43] Gao T, Han Y, Yu L, Ao S, Li Z, Ji J (2014). CCNA2 is a prognostic biomarker for ER + breast cancer and tamoxifen resistance. PLoS ONE.

[44] Wu Y, Li H, Wang H, Zhang F, Cao H, Xu S (2019). MSK2 promotes proliferation and tumor formation in squamous cervical cancer via PAX8/RB-E2F1/cyclin A2 axis. J Cell Biochem..

[45] Shekhar R, Priyanka P, Kumar P, Ghosh T, Khan MM, Nagarajan P (2019). The microRNAs miR-449a and miR-424 suppress osteosarcoma by targeting cyclin A2 expression. J Biol Chem..

[46] Shi Q, Wang W, Jia Z, Chen P, Ma K, Zhou C (2016). ISL1, a novel regulator of CCNB1, CCNB2 and c-MYC genes, promotes gastric cancer cell proliferation and tumor growth. Oncotarget.

[47] Yang X, Mo W, Fang Y, Wei G, Wei M, Dang Y (2018). Up-regulation of Polo-like Kinase 1 in nasopharyngeal carcinoma tissues: a comprehensive investigation based on RNA-sequencing, gene chips, and in-house tissue arrays. Am J Transl Res.

[48] Qian X, Song X, He Y, Yang Z, Sun T, Wang J (2015). CCNB2 overexpression is a poor prognostic biomarker in Chinese NSCLC patients. Biomed Pharmacother.

[49] Chou CH, Yang NK, Liu TY, Tai SK, Hsu DS, Chen YW (2013). Chromosome instability modulated by BMI1-AURKA signaling drives progression in head and neck cancer. Cancer Res.

[50] Hou D, Che Z, Chen P, Zhang W, Chu Y, Yang D (2018). Suppression of AURKA alleviates p27 inhibition on Bax cleavage and induces more intensive apoptosis in gastric cancer. Cell Death Dis.

[51] Sehdev V, Katsha A, Arras J, Peng D, Soutto M, Ecsedy J (2014). HDM2 regulation by AURKA promotes cell survival in gastric cancer. Clin Cancer Res.

[52] Puig-Butille JA, Vinyals A, Ferreres JR, Aguilera P, Cabre E, Tell-Marti G (2017). AURKA overexpression is driven by FOXM1 and MAPK/ERK activation in melanoma cells harboring BRAF or NRAS mutations: impact on melanoma prognosis and therapy. J Invest Dermatol.

[53] Chen C, Song G, Xiang J, Zhang H, Zhao S, Zhan Y (2017). AURKA promotes cancer metastasis by regulating epithelial-mesenchymal transition and cancer stem cell properties in hepatocellular carcinoma. Biochem Biophys Res Commun.

[54] Zheng F, Yue C, Li G, He B, Cheng W, Wang X (2016). Nuclear AURKA acquires kinase-independent transactivating function to enhance breast cancer stem cell phenotype. Nat Commun.

