# Supplementary material for: Joint bioinformatics analysis of underlying potential functions of hsa-let-7b-5p and core genes in human glioma
Source: J Transl Med. 2019 Apr 17;17:129. doi: 10.1186/s12967-019-1882-7 (PMC6471881; doi:10.1186/s12967-019-1882-7)
Supplement: Supplementary file 1 — Additional file 1: Figure S1. P53 mutation in Glioma patients. Figure S2. Mutation site information of p53 in Glioma patients. Table S1. Primers used for quantitative real-time polymerase chain reaction. Table S2. Differentially expressed genes between normal brain and glioma samples. Table S3. Gene ontology enrichment analysis of upregulated and downregulated genes. Table S4. KEGG pathway enrichment analysis of upregulated and downregulated genes. Table S5. Cross-talk genes involved in different pathways. Table S6. Hub genes in the PPI network with degree more than 100. Table S7. Differentially expressed miRNAs between normal brain and glioma samples. [file 12967_2019_1882_MOESM1_ESM.docx]

**Additional file**

**Joint bioinformatics analysis of underlying potential functions of hsa-let-7b-5p and core genes in human glioma**

Xiaonan Xi^1,2^, Yahui Chu^1,2^, Ning Liu^2^, Qianqian Wang^2^, Zheng Yin^1^, Yaxin Lu^1,2*^, Yue Chen^1,2*^

^1^ College of Pharmacy, Nankai University, Tianjin, 300350, P. R. China

^2^ State Key Laboratory of Medicinal Chemical Biology, Nankai University, Tianjin, 300350, P. R. China

### Xiaonan Xi: [xi­_xiaonan@foxmail.com](mailto:xi_xiaonan@foxmail.com); Yahui Chu: [18822141796@163.com](mailto:18822141796@163.com); Ning Liu: [liuning@nankai.edu.cn](mailto:liuning@nankai.edu.cn); Qianqian Wang: [lx_wangqianqian@163.com](mailto:lx_wangqianqian@163.com); Zheng Yin: [yinzheng@mail.nankai.edu.cn](mailto:yinzheng@mail.nankai.edu.cn); Yaxin Lu: [yaxinlu@nankai.edu.cn](mailto:yaxinlu@nankai.edu.cn); Yue Chen: yuechen@nankai.edu.cn.

### ^*^Corresponding authors


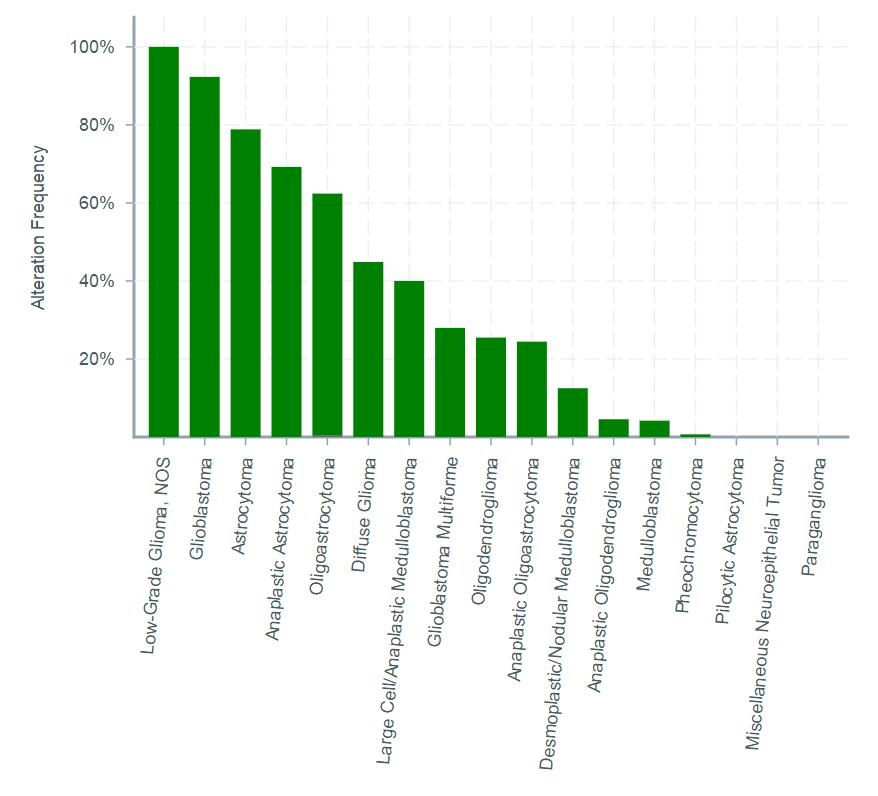


**Figure S1.** P53 mutation in Glioma patients.


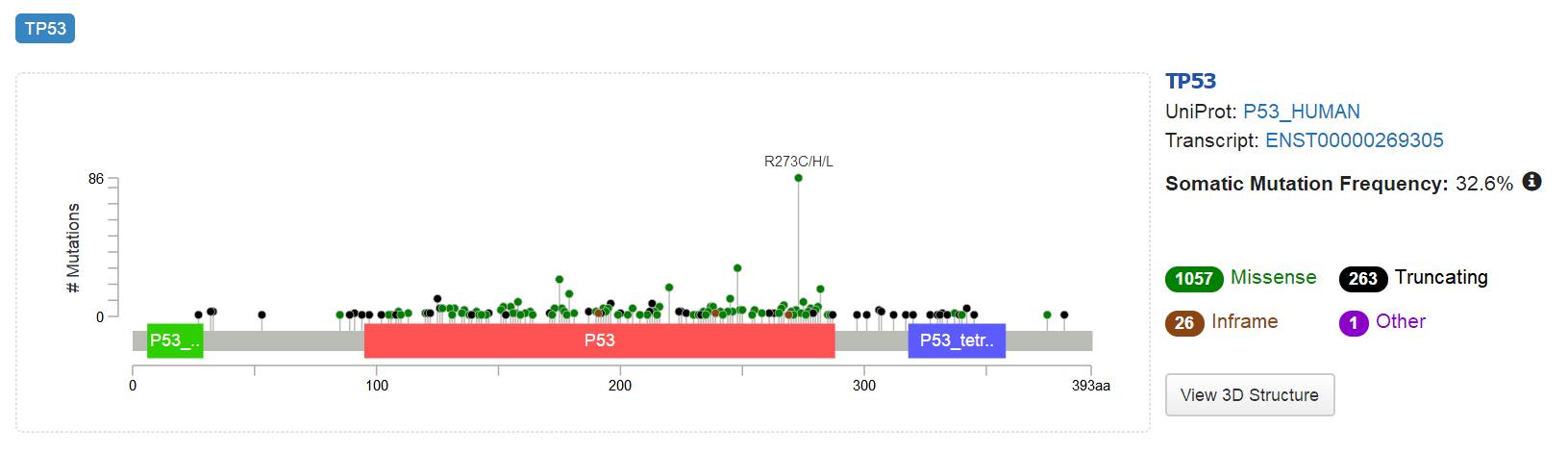


**Figure S2.** Mutation site information of p53 in Glioma patients.

**Table S1.** Primers used for quantitative real-time polymerase chain reaction

| Name | Primer |
| --- | --- |
| PLK1 | (F) 5’-GCACAGCACAGTGTCAATGCCTCCAAG-3’ |
|  | (R) 5’-GCCGTACTTGTCCGAATAGTCC-3’ |
| CCNA2 | (F) 5’-CTCTACACAGTCACGGGACAAAG-3’ |
|  | (R) 5’-CTGTGGTGCTTTGAGGTAGGTC-3’ |
| CCNB2 | (F) 5’-CAACCAGAGCAGCACAAGTAGC-3’ |
|  | (R) 5’-GGAGCCAACTTTTCCATCTGTAC-3’ |
| AURKA | (F) 5’-GCAACCAGTGTACCTCATCCTG-3’ |
|  | (R) 5’-AAGTCTTCCAAAGCCCACTGCC-3’ |
| GAPDH | (F) 5’-GTCTCCTCTGACTTCAACAGCG-3’ |
|  | (R) 5’-ACCACCCTGTTGCTGTAGCCAA-3’ |

**Table S2.** Differentially expressed genes between normal brain and glioma samples.

| Gene Name | logFC | PValue | FDR |
| --- | --- | --- | --- |
| A2BP1 | -3.18364 | 6.63E-08 | 1.52E-06 |
| AACSL | -2.66414 | 1.69E-06 | 2.34E-05 |
| AATK | -2.31215 | 5.12E-08 | 1.24E-06 |
| ABCA1 | 2.238901 | 2.51E-06 | 3.29E-05 |
| ABCA13 | 4.090912 | 0.008794 | 0.02964 |
| ABCA2 | -2.06323 | 5.42E-09 | 1.87E-07 |
| ABCA5 | -2.06051 | 1.91E-05 | 0.000185 |
| ABCB4 | 2.18154 | 0.000345 | 0.002098 |
| ABCC12 | -2.87223 | 0.000106 | 0.000782 |
| ABCG4 | -2.78303 | 9.15E-09 | 2.87E-07 |
| ABCG8 | -2.95556 | 5.4E-06 | 6.32E-05 |
| ABHD4 | 2.006298 | 2.63E-10 | 1.49E-08 |
| ABI3BP | 2.529567 | 0.005974 | 0.021865 |
| ABLIM2 | -2.29688 | 5.44E-09 | 1.87E-07 |
| ABRA | -2.37276 | 0.000261 | 0.001659 |
| ACCN1 | -2.86836 | 6.94E-07 | 0.000011 |
| ACCN4 | 2.7801 | 0.006377 | 0.02303 |
| ACOT7 | -2.07479 | 1.78E-13 | 4.52E-11 |
| ACTA1 | -2.26592 | 3.08E-08 | 8.07E-07 |
| ACVR1C | -2.5334 | 7.67E-07 | 0.000012 |
| ADAD2 | -3.5064 | 1.24E-11 | 1.34E-09 |
| ADAM11 | -2.98937 | 6.09E-11 | 4.43E-09 |
| ADAMDEC1 | 9.528845 | 0.001303 | 0.006321 |
| ADAMTS12 | 4.210698 | 1.52E-05 | 0.000152 |
| ADAMTS15 | 2.883971 | 0.008591 | 0.029116 |
| ADAMTS16 | -2.31567 | 8.22E-05 | 0.000631 |
| ADAMTS5 | 2.308972 | 9.79E-05 | 0.000731 |
| ADAMTS6 | 3.512524 | 2.15E-07 | 4.08E-06 |
| ADAP1 | -2.62323 | 1.94E-11 | 1.9E-09 |
| ADCY1 | -2.43401 | 1.41E-06 | 2.01E-05 |
| ADCYAP1 | -2.69087 | 9.39E-06 | 0.000101 |
| ADRA1B | -3.09082 | 1E-14 | 5.56E-12 |
| ADRA2C | -2.04777 | 4.55E-06 | 5.48E-05 |
| ADRB1 | -2.05977 | 2.12E-07 | 4.04E-06 |
| ADRB3 | -3.86881 | 2.87E-12 | 4.41E-10 |
| AFAP1L1 | 2.159499 | 0.002913 | 0.012225 |
| AGAP2 | -2.26456 | 2.45E-06 | 3.24E-05 |
| AGBL4 | -2.67246 | 5.47E-09 | 1.88E-07 |
| AIFM3 | -2.00591 | 0.001112 | 0.005553 |
| AIM2 | 3.08246 | 0.000233 | 0.001502 |
| AIPL1 | -2.64915 | 2.13E-05 | 0.000203 |
| AK5 | -3.17729 | 1.22E-09 | 5.41E-08 |
| AKAP5 | -3.07204 | 5.51E-14 | 1.81E-11 |
| AKNAD1 | 2.478351 | 0.015603 | 0.046458 |
| ALAS2 | -2.3495 | 0.003626 | 0.014594 |
| ALOX15B | 4.102763 | 0.005371 | 0.020056 |
| ALOXE3 | -2.7341 | 1.59E-08 | 4.56E-07 |
| AMH | 2.78686 | 0.002944 | 0.012317 |
| AMPH | -2.70468 | 7.34E-12 | 9.05E-10 |
| ANG | 2.093002 | 0.012129 | 0.038291 |
| ANGPT2 | 2.708803 | 0.006888 | 0.024493 |
| ANGPTL2 | 2.671039 | 9.63E-05 | 0.000722 |
| ANK3 | -2.38931 | 2.07E-07 | 3.98E-06 |
| ANKFN1 | 3.282653 | 1.79E-06 | 2.46E-05 |
| ANKRD2 | -2.977 | 3.27E-08 | 8.43E-07 |
| ANKRD30B | -3.76024 | 1.62E-09 | 6.74E-08 |
| ANKRD34A | -2.69843 | 5.84E-11 | 4.32E-09 |
| ANKRD34C | -2.82801 | 3.63E-05 | 0.000319 |
| ANKRD43 | -2.92458 | 1.41E-09 | 6.09E-08 |
| ANKRD56 | -3.36706 | 4.08E-08 | 1.01E-06 |
| ANKS1B | -2.11297 | 8.35E-07 | 1.28E-05 |
| ANO3 | -3.13982 | 5.5E-09 | 1.89E-07 |
| ANO4 | -2.34732 | 7.91E-07 | 1.23E-05 |
| ANTXR1 | 2.307247 | 7.68E-11 | 5.4E-09 |
| ANTXR2 | 2.168841 | 0.0002 | 0.001324 |
| ANXA2 | 2.37364 | 0.013586 | 0.04169 |
| ANXA8 | -3.21534 | 1.27E-07 | 2.62E-06 |
| ANXA8L2 | -3.05487 | 3.03E-07 | 5.43E-06 |
| AP1M2 | -2.28013 | 0.000839 | 0.004398 |
| APBA1 | -2.04872 | 1.19E-10 | 7.74E-09 |
| APLP1 | -2.29245 | 5.16E-12 | 6.83E-10 |
| APOBEC3B | 4.501086 | 2.72E-05 | 0.00025 |
| APOBEC3C | 2.40856 | 0.001768 | 0.008134 |
| APOC1 | 2.723072 | 5.15E-05 | 0.000426 |
| APOC1P1 | 3.35794 | 0.005156 | 0.019422 |
| APOC4 | 2.976645 | 0.002374 | 0.010335 |
| APOL4 | 3.285313 | 0.012246 | 0.038565 |
| AQP12B | -2.48926 | 0.000677 | 0.00369 |
| AQP3 | -2.95608 | 1.39E-10 | 8.76E-09 |
| AQP7P1 | 3.741699 | 0.015791 | 0.046853 |
| AQP7P3 | 6.023586 | 0.004737 | 0.0181 |
| AR | 3.144399 | 0.001037 | 0.005248 |
| ARHGAP11A | 2.586663 | 0.000894 | 0.004649 |
| ARHGAP11B | 2.726875 | 0.002228 | 0.009814 |
| ARHGAP42 | 2.084171 | 5.17E-06 | 6.09E-05 |
| ARHGDIG | -2.6906 | 6.6E-08 | 1.52E-06 |
| ARHGEF33 | -2.11995 | 1.06E-11 | 1.19E-09 |
| ARX | -2.29655 | 1.31E-05 | 0.000134 |
| ASB2 | -2.34677 | 1.81E-05 | 0.000177 |
| ASCL1 | 2.992376 | 6.19E-06 | 7.08E-05 |
| ASF1B | 4.830075 | 4.32E-05 | 0.000368 |
| ASIP | 4.144176 | 0.000278 | 0.001753 |
| ASPDH | -2.28698 | 2.65E-06 | 3.44E-05 |
| ASPM | 4.320068 | 0.000751 | 0.004005 |
| ASTL | -2.75648 | 0.000011 | 0.000116 |
| ATL1 | -2.06259 | 7.13E-13 | 1.45E-10 |
| ATOH7 | -2.81928 | 5.53E-11 | 4.21E-09 |
| ATOH8 | 2.867128 | 0.000929 | 0.004794 |
| ATP1A3 | -2.53801 | 1.04E-08 | 3.21E-07 |
| ATP1B1 | -2.37585 | 9.29E-11 | 6.28E-09 |
| ATP1B4 | -2.34374 | 0.001709 | 0.007922 |
| ATP2B1 | -2.03718 | 1.82E-09 | 7.34E-08 |
| ATP2B2 | -2.0906 | 1.31E-06 | 1.89E-05 |
| ATP2B3 | -3.07663 | 2.93E-07 | 5.3E-06 |
| ATP4A | -2.79581 | 0.000466 | 0.002706 |
| ATP5EP2 | 2.336668 | 0.004841 | 0.018411 |
| ATP6V1B1 | 4.130305 | 8.69E-07 | 1.33E-05 |
| ATP8A2 | -3.44397 | 2.49E-09 | 9.51E-08 |
| AURKA | 2.210063 | 0.003823 | 0.01525 |
| AURKB | 6.467966 | 3.03E-05 | 0.000273 |
| B3GNT4 | -3.22108 | 7.7E-11 | 5.4E-09 |
| B4GALNT3 | -2.20938 | 7.77E-06 | 8.59E-05 |
| BARD1 | 2.036358 | 0.001018 | 0.005175 |
| BARHL1 | 7.844773 | 0.001189 | 0.005862 |
| BCAN | 3.180281 | 1.33E-05 | 0.000137 |
| BCHE | 2.514635 | 5.14E-07 | 8.44E-06 |
| BCL11A | -2.8896 | 1.05E-08 | 3.23E-07 |
| BCL2L10 | -2.83513 | 1.47E-08 | 4.26E-07 |
| BCL2L12 | 2.04313 | 0.003417 | 0.013896 |
| BCMO1 | 2.488962 | 0.001412 | 0.006744 |
| BDNF | -3.09708 | 3.1E-09 | 1.15E-07 |
| BEGAIN | -2.60213 | 1.23E-09 | 5.46E-08 |
| BEST3 | 4.198313 | 6.56E-06 | 7.43E-05 |
| BEX5 | -2.61866 | 9.12E-08 | 0.000002 |
| BFSP1 | -2.44713 | 6.41E-09 | 2.16E-07 |
| BHLHE22 | -3.61139 | 5.4E-11 | 4.15E-09 |
| BHMT | -2.95229 | 1.2E-08 | 3.6E-07 |
| BIRC5 | 4.889553 | 0.000476 | 0.002749 |
| BMF | 2.452142 | 0.000053 | 0.000436 |
| BMP2 | 3.280515 | 0.000357 | 0.002159 |
| BOK | -2.04361 | 2.68E-05 | 0.000248 |
| BPIL1 | 8.436647 | 0.001093 | 0.005478 |
| BRCA2 | 2.527317 | 0.001653 | 0.007696 |
| BRIP1 | 2.798153 | 0.007259 | 0.025482 |
| BRSK1 | -2.02426 | 2.79E-11 | 2.43E-09 |
| BSN | -2.87928 | 1.04E-11 | 1.18E-09 |
| BTBD16 | -2.32357 | 0.000129 | 0.000914 |
| BTBD17 | 3.127679 | 0.000712 | 0.003839 |
| BTN2A3 | 2.387706 | 0.000146 | 0.00101 |
| BTN3A2 | 2.316681 | 0.000034 | 0.0003 |
| BUB1 | 5.118163 | 2.73E-05 | 0.000251 |
| BUB1B | 4.630067 | 0.000284 | 0.001784 |
| C10orf35 | -2.23422 | 3.06E-07 | 5.48E-06 |
| C10orf55 | 2.730775 | 0.010316 | 0.033684 |
| C10orf81 | 6.179432 | 0.005737 | 0.021135 |
| C10orf82 | -2.85117 | 5.25E-07 | 8.59E-06 |
| C11orf70 | 2.805893 | 0.006617 | 0.023679 |
| C11orf82 | 2.764555 | 0.000474 | 0.002743 |
| C11orf87 | -3.26874 | 3.73E-08 | 9.4E-07 |
| C11orf9 | -2.40763 | 2.96E-06 | 3.79E-05 |
| C11orf90 | -2.33574 | 8.65E-05 | 0.000658 |
| C13orf36 | -3.79648 | 4.02E-11 | 3.31E-09 |
| C14orf162 | -2.60372 | 1.91E-06 | 0.000026 |
| C14orf64 | 2.438145 | 8.22E-07 | 1.27E-05 |
| C15orf27 | -2.542 | 3.97E-10 | 2.06E-08 |
| C15orf42 | 2.415259 | 0.000138 | 0.00097 |
| C16orf73 | -2.52533 | 2.48E-06 | 3.26E-05 |
| C17orf107 | -2.5888 | 3.06E-11 | 2.61E-09 |
| C17orf28 | -2.00877 | 1.44E-10 | 9.02E-09 |
| C17orf82 | 2.337979 | 0.001811 | 0.008292 |
| C17orf87 | 2.309275 | 0.004328 | 0.016864 |
| C17orf88 | -2.7907 | 1.38E-06 | 1.97E-05 |
| C19orf30 | -2.93501 | 2.79E-06 | 0.000036 |
| C19orf51 | 2.695664 | 0.01666 | 0.048877 |
| C1orf106 | 2.141152 | 0.00062 | 0.003435 |
| C1orf115 | -3.03083 | 1.73E-11 | 1.76E-09 |
| C1orf161 | -2.18088 | 0.000763 | 0.004056 |
| C1orf162 | 2.053214 | 0.002854 | 0.01203 |
| C1orf173 | -2.85287 | 3.61E-07 | 6.26E-06 |
| C1orf201 | -2.11965 | 4.57E-06 | 5.51E-05 |
| C1orf226 | 2.88398 | 5.86E-08 | 1.38E-06 |
| C1orf59 | -2.04096 | 8.14E-08 | 1.82E-06 |
| C1orf94 | 2.937157 | 2.8E-06 | 3.61E-05 |
| C1QL1 | 3.16027 | 0.00036 | 0.002176 |
| C1QL3 | -4.02769 | 2.68E-11 | 2.38E-09 |
| C1QTNF1 | 2.635433 | 0.00152 | 0.007156 |
| C1QTNF4 | -2.87854 | 4.7E-11 | 3.73E-09 |
| C20orf103 | -2.38805 | 2.47E-08 | 6.68E-07 |
| C21orf128 | -2.1382 | 0.003633 | 0.014614 |
| C21orf62 | 3.379756 | 0.001996 | 0.008971 |
| C21orf82 | 2.777509 | 0.001314 | 0.006367 |
| C21orf99 | -2.50756 | 0.000599 | 0.003337 |
| C22orf24 | -2.33087 | 2.48E-08 | 6.69E-07 |
| C2CD4B | -2.38323 | 2.94E-05 | 0.000267 |
| C2CD4C | -2.27683 | 8.67E-09 | 2.75E-07 |
| C2CD4D | -2.20602 | 2.5E-06 | 3.29E-05 |
| C2orf27A | 2.314329 | 0.001789 | 0.008211 |
| C2orf55 | -2.68181 | 1.51E-12 | 2.73E-10 |
| C2orf89 | -2.73364 | 1.17E-08 | 3.54E-07 |
| C3orf50 | 2.180647 | 0.006705 | 0.023952 |
| C4orf44 | -2.79753 | 3.2E-13 | 7.54E-11 |
| C4orf47 | 3.107056 | 0.001442 | 0.00686 |
| C4orf50 | -3.29315 | 6.25E-08 | 1.46E-06 |
| C5orf38 | 6.63443 | 0.000682 | 0.003708 |
| C5orf39 | 2.162052 | 0.001374 | 0.006598 |
| C5orf40 | -3.30615 | 1.72E-10 | 1.05E-08 |
| C6orf114 | -2.28292 | 1.81E-09 | 7.32E-08 |
| C6orf154 | -2.42657 | 1.18E-10 | 7.71E-09 |
| C6orf176 | -2.11836 | 0.006137 | 0.022317 |
| C6orf222 | -2.92887 | 3.17E-06 | 4.02E-05 |
| C6orf227 | -2.31957 | 0.000181 | 0.001216 |
| C6orf27 | -3.35272 | 4.19E-11 | 3.39E-09 |
| C6orf58 | 4.412225 | 0.001841 | 0.008394 |
| C8orf85 | -2.21944 | 0.000222 | 0.001442 |
| C9orf171 | 5.313957 | 0.000471 | 0.002726 |
| C9orf172 | -2.1925 | 1.6E-11 | 1.64E-09 |
| C9orf4 | -2.46842 | 1.59E-05 | 0.000158 |
| C9orf84 | 3.179807 | 0.001932 | 0.008728 |
| CA11 | -2.70241 | 1.22E-11 | 1.33E-09 |
| CA12 | 2.791148 | 0.008976 | 0.030116 |
| CA3 | 4.920625 | 0.015674 | 0.04659 |
| CA7 | -2.78794 | 1.58E-05 | 0.000158 |
| CABP1 | -2.53795 | 1.55E-05 | 0.000155 |
| CABYR | -2.06774 | 8.12E-08 | 1.81E-06 |
| CACNA1B | -3.24908 | 9.56E-09 | 2.98E-07 |
| CACNA1C | -2.16351 | 1.09E-09 | 4.88E-08 |
| CACNA1E | -2.41963 | 5.29E-07 | 8.64E-06 |
| CACNA1F | -3.07277 | 6.51E-12 | 8.37E-10 |
| CACNA1G | -2.02729 | 6.86E-05 | 0.000541 |
| CACNA1I | -2.82304 | 3.52E-07 | 6.13E-06 |
| CACNA1S | -2.07314 | 0.004707 | 0.018015 |
| CACNA2D3 | -2.42162 | 1.28E-05 | 0.000131 |
| CACNB1 | -2.5126 | 4.24E-14 | 1.47E-11 |
| CACNB2 | -2.00118 | 2.74E-07 | 5.01E-06 |
| CACNB3 | -2.22784 | 5.83E-11 | 4.32E-09 |
| CACNG3 | -3.2805 | 1.02E-06 | 1.51E-05 |
| CACNG6 | 2.322402 | 0.004005 | 0.015828 |
| CACNG8 | -3.10598 | 4.09E-09 | 1.48E-07 |
| CALB2 | -2.8928 | 7.67E-07 | 0.000012 |
| CALCRL | 3.140781 | 2.51E-05 | 0.000233 |
| CALHM1 | -3.4986 | 2.01E-08 | 5.58E-07 |
| CALM3 | -2.06183 | 2.68E-14 | 1.19E-11 |
| CALY | -3.1781 | 3.65E-07 | 6.31E-06 |
| CAMK1D | -2.14191 | 4.51E-12 | 6.3E-10 |
| CAMK1G | -2.96679 | 5.23E-06 | 6.15E-05 |
| CAMK2A | -3.1899 | 6.57E-07 | 1.05E-05 |
| CAMK2B | -2.76759 | 5.54E-10 | 2.7E-08 |
| CAMK2N1 | -2.23589 | 5.29E-11 | 4.08E-09 |
| CAMK4 | -2.06672 | 0.00028 | 0.00176 |
| CAMKK1 | -3.00895 | 5.44E-12 | 7.1E-10 |
| CAMKK2 | -2.03182 | 1.54E-10 | 9.54E-09 |
| CAMKV | -3.1989 | 3.94E-10 | 2.06E-08 |
| CAP2 | -2.43398 | 1.04E-09 | 4.7E-08 |
| CAPN13 | -2.70824 | 0.000066 | 0.000525 |
| CARNS1 | -2.78479 | 1.58E-06 | 2.21E-05 |
| CASC5 | 4.764948 | 0.000111 | 0.000807 |
| CASKIN1 | -2.04525 | 3.19E-06 | 4.04E-05 |
| CASP1 | 2.09871 | 0.003901 | 0.015485 |
| CASP4 | 2.15219 | 0.011281 | 0.036176 |
| CASZ1 | 2.522111 | 0.0004 | 0.002376 |
| CBFA2T3 | -2.01103 | 8.62E-08 | 1.91E-06 |
| CBLN4 | -3.08383 | 2.28E-06 | 3.02E-05 |
| CBX2 | 3.123072 | 4.27E-05 | 0.000365 |
| CCBE1 | -3.53323 | 5.24E-09 | 1.82E-07 |
| CCDC64 | -2.30773 | 3.97E-06 | 4.88E-05 |
| CCDC64B | -2.04535 | 0.000984 | 0.005032 |
| CCDC80 | 3.534057 | 5.34E-09 | 1.85E-07 |
| CCK | -3.14396 | 5.83E-07 | 9.44E-06 |
| CCKBR | -3.47107 | 6.63E-09 | 2.21E-07 |
| CCL14 | 3.654948 | 0.006304 | 0.022832 |
| CCL25 | 3.435994 | 0.006323 | 0.022887 |
| CCL28 | -2.11549 | 0.000221 | 0.00144 |
| CCNA1 | -2.48766 | 2.32E-05 | 0.000218 |
| CCNA2 | 3.100469 | 0.001067 | 0.005369 |
| CCNB2 | 6.194204 | 2.59E-05 | 0.000239 |
| CD164L2 | -2.71542 | 0.000549 | 0.003099 |
| CD177 | -2.14336 | 0.005077 | 0.019176 |
| CD180 | 2.083031 | 0.004892 | 0.018574 |
| CD1D | 2.630791 | 0.001058 | 0.00533 |
| CD207 | 4.902466 | 0.000106 | 0.000781 |
| CD22 | -2.85843 | 1.33E-07 | 2.72E-06 |
| CD244 | -2.50187 | 5.48E-08 | 1.31E-06 |
| CD38 | 2.120893 | 0.005103 | 0.019269 |
| CD44 | 2.304991 | 0.005736 | 0.021135 |
| CD99 | 2.272665 | 0.001898 | 0.0086 |
| CDC25A | 2.151295 | 0.000979 | 0.005018 |
| CDC25C | 4.635413 | 0.000373 | 0.00224 |
| CDC45 | 6.620961 | 1.47E-05 | 0.000148 |
| CDCA2 | 4.707655 | 0.000193 | 0.001285 |
| CDCA7 | 3.569228 | 4.65E-06 | 5.58E-05 |
| CDCA7L | 2.151826 | 0.002894 | 0.012158 |
| CDCA8 | 3.191354 | 0.002478 | 0.010723 |
| CDH12 | -2.983 | 7.57E-09 | 2.46E-07 |
| CDH15 | 4.994236 | 0.002417 | 0.010497 |
| CDH18 | -2.19513 | 0.00041 | 0.002422 |
| CDH22 | -2.24731 | 2.23E-05 | 0.000211 |
| CDH8 | -2.94039 | 2.71E-08 | 7.25E-07 |
| CDH9 | -3.39529 | 1.59E-08 | 4.56E-07 |
| CDHR2 | -2.50286 | 2.23E-07 | 4.19E-06 |
| CDK1 | 3.978298 | 0.000176 | 0.00119 |
| CDK2 | 2.711476 | 0.00002 | 0.000193 |
| CDK4 | 3.491563 | 0.002267 | 0.009957 |
| CDK5R2 | -2.30988 | 9.23E-06 | 9.96E-05 |
| CDKL2 | -2.75416 | 6.13E-08 | 1.43E-06 |
| CDKL5 | -2.80217 | 3.59E-07 | 6.25E-06 |
| CDKN2C | 2.242741 | 0.007228 | 0.025403 |
| CDKN2D | -2.12624 | 2.69E-12 | 4.23E-10 |
| CDS1 | -2.04479 | 5.02E-05 | 0.000417 |
| CELF4 | -2.86114 | 1.71E-07 | 3.37E-06 |
| CELF5 | -2.4182 | 2.1E-07 | 4.02E-06 |
| CELSR1 | 3.298656 | 0.008523 | 0.028952 |
| CENPA | 4.878114 | 0.000378 | 0.002263 |
| CENPE | 2.316647 | 0.016506 | 0.04852 |
| CENPF | 2.88939 | 0.003193 | 0.013173 |
| CENPH | 2.297238 | 0.000105 | 0.000773 |
| CENPI | 3.165499 | 0.000714 | 0.003847 |
| CENPK | 4.442052 | 0.00025 | 0.001594 |
| CENPM | 3.57098 | 0.000066 | 0.000525 |
| CEP55 | 4.774811 | 0.000113 | 0.000822 |
| CERCAM | -2.15195 | 3.43E-08 | 8.8E-07 |
| CERKL | 2.123322 | 0.01489 | 0.044856 |
| CES7 | -2.02286 | 0.000195 | 0.001292 |
| CES8 | -3.03997 | 4.81E-12 | 6.52E-10 |
| CETP | 3.072426 | 0.000727 | 0.003903 |
| CFC1B | -3.03577 | 0.000167 | 0.001132 |
| CFI | 2.352129 | 0.015742 | 0.046748 |
| CHD5 | -3.39025 | 7.82E-10 | 3.64E-08 |
| CHEK2 | 2.306723 | 0.000272 | 0.001719 |
| CHGA | -2.06677 | 0.00146 | 0.006922 |
| CHN1 | -2.58096 | 1.35E-09 | 5.84E-08 |
| CHP2 | -2.46123 | 0.00003 | 0.000271 |
| CHRM1 | -2.96169 | 1.94E-07 | 3.75E-06 |
| CHRM2 | -3.12895 | 2.64E-07 | 4.85E-06 |
| CHRM3 | -2.88167 | 8.43E-07 | 1.29E-05 |
| CHRNA2 | -2.70266 | 0.00011 | 0.000801 |
| CHRNB3 | -2.61357 | 0.000321 | 0.001975 |
| CHST9 | 4.15167 | 6.13E-05 | 0.000493 |
| CIDEA | -2.45768 | 0.001718 | 0.007953 |
| CILP | 2.740837 | 0.013261 | 0.040906 |
| CKAP2L | 3.44534 | 0.003022 | 0.012586 |
| CKMT1A | -2.88382 | 6.85E-08 | 1.56E-06 |
| CKMT1B | -2.47751 | 2.54E-06 | 3.32E-05 |
| CKS2 | 2.772859 | 0.000432 | 0.002535 |
| CLCN4 | -2.26829 | 3.65E-12 | 5.49E-10 |
| CLDN11 | -2.16091 | 2.84E-05 | 0.000259 |
| CLDN16 | -2.95789 | 3.32E-06 | 4.18E-05 |
| CLDN7 | 2.495314 | 0.001702 | 0.007893 |
| CLEC2L | -2.53018 | 9.32E-05 | 0.000702 |
| CLEC4E | 4.260663 | 0.000341 | 0.002077 |
| CLEC4F | 5.06078 | 0.00015 | 0.001035 |
| CLEC4G | -3.05495 | 2.89E-06 | 3.71E-05 |
| CLEC4GP1 | -2.33326 | 0.001437 | 0.00684 |
| CLMN | -2.15953 | 3.26E-10 | 1.77E-08 |
| CLSTN3 | -2.51548 | 4.54E-15 | 2.99E-12 |
| CLUL1 | -2.05981 | 0.001065 | 0.005359 |
| CMTM3 | 2.665601 | 7.47E-10 | 3.49E-08 |
| CNDP1 | -2.2502 | 0.000345 | 0.002098 |
| CNGA3 | 3.364647 | 0.006009 | 0.021961 |
| CNGB1 | -4.18429 | 1.8E-09 | 7.31E-08 |
| CNKSR2 | -2.71472 | 4.8E-11 | 3.78E-09 |
| CNNM1 | -2.53011 | 1.12E-05 | 0.000118 |
| CNTN2 | -2.56303 | 1.19E-06 | 1.74E-05 |
| CNTN4 | -2.49683 | 1.08E-06 | 1.59E-05 |
| CNTN5 | -3.15156 | 6.62E-08 | 1.52E-06 |
| CNTNAP1 | -2.03602 | 9.03E-12 | 1.06E-09 |
| CNTNAP2 | -2.33303 | 7.03E-06 | 7.86E-05 |
| COL19A1 | -2.3482 | 0.000115 | 0.000836 |
| COL24A1 | -2.73384 | 2.16E-07 | 4.08E-06 |
| COL28A1 | 3.746897 | 0.006149 | 0.022337 |
| COL4A1 | 3.63442 | 0.004315 | 0.016832 |
| COL4A2 | 2.668871 | 0.01167 | 0.037175 |
| COL4A6 | 2.906415 | 0.005171 | 0.019467 |
| COL6A6 | -2.05776 | 3.71E-07 | 6.4E-06 |
| CORO2A | -2.15943 | 1.88E-06 | 2.57E-05 |
| CORT | -2.44807 | 7.71E-09 | 2.49E-07 |
| CPLX1 | -2.23818 | 6.51E-06 | 7.39E-05 |
| CPLX2 | -2.66748 | 1.16E-05 | 0.000121 |
| CPLX3 | -2.93226 | 1.26E-06 | 1.82E-05 |
| CPNE6 | -3.45033 | 2.77E-09 | 1.05E-07 |
| CPNE7 | -3.45666 | 8.58E-11 | 5.93E-09 |
| CPNE9 | -3.32856 | 6.17E-09 | 2.09E-07 |
| CPVL | 2.284274 | 0.003439 | 0.013971 |
| CPXM1 | 3.864373 | 5.6E-06 | 0.000065 |
| CREG2 | -3.33595 | 7.6E-08 | 1.71E-06 |
| CRH | -3.11879 | 1.2E-06 | 1.74E-05 |
| CRHBP | -2.62561 | 1.6E-07 | 3.21E-06 |
| CRHR1 | -2.39911 | 1.9E-06 | 2.59E-05 |
| CRHR2 | -4.05257 | 7.63E-12 | 9.33E-10 |
| CRISPLD1 | 2.632189 | 0.000135 | 0.000951 |
| CRYGN | -4.29944 | 4.31E-25 | 3.82E-21 |
| CRYM | -3.3334 | 6.66E-08 | 1.53E-06 |
| CSRP2 | 2.770621 | 8.42E-05 | 0.000643 |
| CST6 | -2.1998 | 0.004057 | 0.015973 |
| CSTA | 3.724659 | 0.007829 | 0.027104 |
| CTRB2 | -2.394 | 0.000271 | 0.001712 |
| CXCL13 | 6.165451 | 0.016672 | 0.048901 |
| CXCR2 | 2.785852 | 0.004143 | 0.016246 |
| CXCR4 | 2.089656 | 0.002481 | 0.010727 |
| CXorf64 | 5.090513 | 0.000561 | 0.003156 |
| CYGB | -2.43344 | 9.35E-07 | 1.41E-05 |
| CYP19A1 | 3.572918 | 0.00187 | 0.008497 |
| CYP1A1 | -2.12961 | 6.89E-05 | 0.000543 |
| CYP26A1 | -2.19552 | 0.000209 | 0.001372 |
| CYP26B1 | -2.67607 | 2.13E-07 | 4.05E-06 |
| CYP26C1 | -2.35097 | 5.08E-07 | 8.35E-06 |
| CYP2C18 | -4.42145 | 1.61E-15 | 1.36E-12 |
| CYP46A1 | -2.34924 | 2.15E-10 | 1.27E-08 |
| CYP4A11 | -2.10905 | 0.00124 | 0.006077 |
| CYP4X1 | -2.95347 | 1.46E-09 | 6.22E-08 |
| CYP4Z1 | -3.24998 | 6.2E-07 | 9.97E-06 |
| CYTL1 | 2.320051 | 0.011547 | 0.036893 |
| DAPL1 | 3.853213 | 0.016464 | 0.048437 |
| DBH | -2.56892 | 3.45E-07 | 6.04E-06 |
| DCAF12L2 | -2.18946 | 0.001787 | 0.008206 |
| DCLK3 | -2.89867 | 1.22E-07 | 2.54E-06 |
| DCX | 2.392844 | 0.010515 | 0.034193 |
| DDN | -3.29851 | 3.5E-08 | 8.95E-07 |
| DDX12 | 2.677638 | 0.000232 | 0.001496 |
| DENND2D | 2.244842 | 0.008797 | 0.02964 |
| DEPDC1B | 2.604653 | 0.008746 | 0.029519 |
| DGKE | -2.69773 | 5.05E-11 | 3.95E-09 |
| DGKZ | -2.30441 | 3.23E-13 | 7.54E-11 |
| DHDH | -2.71865 | 1.26E-09 | 5.54E-08 |
| DIRAS2 | -2.17689 | 5.01E-06 | 5.92E-05 |
| DKFZP434L187 | 3.276276 | 0.002858 | 0.012045 |
| DLEU1 | 2.318461 | 1.63E-07 | 3.25E-06 |
| DLG2 | -2.45377 | 9.74E-10 | 4.45E-08 |
| DLG4 | -2.18748 | 2.31E-12 | 3.86E-10 |
| DLGAP2 | -3.19756 | 1.27E-08 | 3.78E-07 |
| DLGAP3 | -2.80799 | 5.18E-10 | 2.56E-08 |
| DLGAP5 | 6.949453 | 7.22E-05 | 0.000565 |
| DLL3 | 4.772631 | 5.48E-05 | 0.000449 |
| DMRT2 | -2.08848 | 0.000884 | 0.004607 |
| DNAJA4 | -2.65486 | 3.8E-12 | 5.57E-10 |
| DNAJC5G | -3.39144 | 2.56E-07 | 4.73E-06 |
| DNAJC6 | -2.50413 | 4.34E-13 | 9.52E-11 |
| DNM1 | -3.26961 | 1.93E-12 | 3.35E-10 |
| DOC2A | -3.33029 | 6.82E-11 | 4.86E-09 |
| DOC2B | -2.24412 | 0.000133 | 0.000941 |
| DOCK3 | -2.05171 | 2.08E-08 | 5.77E-07 |
| DPEP1 | 6.737008 | 0.00224 | 0.009867 |
| DPYSL3 | 2.183928 | 1.62E-09 | 6.74E-08 |
| DRAM1 | 2.084145 | 0.000086 | 0.000656 |
| DRD1 | -3.49724 | 1.46E-10 | 9.15E-09 |
| DRD5 | -3.52572 | 8.78E-09 | 2.77E-07 |
| DTL | 4.192181 | 7.91E-05 | 0.000611 |
| DUSP2 | -2.39947 | 1.91E-07 | 3.71E-06 |
| DUSP8 | -2.15937 | 2.12E-10 | 1.25E-08 |
| DYNC1I1 | -2.59344 | 8.18E-09 | 2.61E-07 |
| DYNLT1 | 2.265463 | 9.02E-08 | 1.99E-06 |
| DYSF | -2.3954 | 2.28E-11 | 2.13E-09 |
| E2F2 | 5.051165 | 2.53E-05 | 0.000235 |
| E2F7 | 4.12818 | 0.001425 | 0.006796 |
| E2F8 | 5.51563 | 4.11E-05 | 0.000353 |
| ECM2 | 2.393013 | 0.005505 | 0.020472 |
| EDA2R | 2.532806 | 0.011784 | 0.037478 |
| EDIL3 | -2.33349 | 8.46E-07 | 0.000013 |
| EEF1A2 | -2.70054 | 4.05E-10 | 2.09E-08 |
| EFEMP1 | 2.579815 | 0.002647 | 0.011318 |
| EFHA2 | -2.14 | 1.75E-11 | 1.76E-09 |
| EGFLAM | 2.271401 | 0.009063 | 0.030376 |
| EGFR | 4.533195 | 0.000423 | 0.002485 |
| EGR3 | -2.0193 | 0.00012 | 0.000862 |
| EGR4 | -2.94402 | 2.85E-05 | 0.00026 |
| EIF4E1B | -2.85116 | 5.49E-05 | 0.000449 |
| EIF4EBP1 | 2.967869 | 2.1E-07 | 4.03E-06 |
| ELAVL2 | -2.16761 | 2.03E-05 | 0.000195 |
| ELMOD1 | -2.05786 | 3.79E-07 | 6.48E-06 |
| ELN | 2.290006 | 0.000942 | 0.004852 |
| EME1 | 2.633058 | 0.000231 | 0.001488 |
| EMID2 | -2.29027 | 0.000048 | 0.000402 |
| EMX1 | -2.98246 | 5.04E-06 | 5.95E-05 |
| EN1 | 7.178919 | 0.004779 | 0.01823 |
| ENC1 | -3.32426 | 3.22E-14 | 1.27E-11 |
| ENPEP | 2.88583 | 0.002767 | 0.011732 |
| ENPP2 | -2.57588 | 1.01E-07 | 2.16E-06 |
| ENTPD3 | -2.612 | 0.000016 | 0.00016 |
| EPB41L3 | -2.01165 | 6.29E-08 | 1.46E-06 |
| EPB41L4B | -2.75258 | 7.35E-10 | 3.44E-08 |
| EPB49 | -2.84848 | 3.25E-10 | 1.77E-08 |
| EPCAM | -2.53806 | 2.99E-08 | 7.91E-07 |
| EPHA10 | -2.34937 | 5.57E-06 | 6.48E-05 |
| EPHA5 | -2.56205 | 5.23E-07 | 8.57E-06 |
| EPHA6 | -2.99052 | 1.87E-06 | 2.55E-05 |
| EPHB6 | -2.77066 | 1.28E-07 | 2.63E-06 |
| EPHX4 | -2.33685 | 7.77E-08 | 1.75E-06 |
| EPN3 | -2.14268 | 0.002278 | 0.009999 |
| EPR1 | 5.570848 | 0.000119 | 0.000859 |
| ERC2 | -2.87629 | 2.99E-15 | 2.04E-12 |
| ERCC6L | 2.954549 | 0.001445 | 0.006871 |
| ERMN | -2.31002 | 0.000102 | 0.000757 |
| ERP27 | 2.896683 | 0.01064 | 0.034494 |
| ESCO2 | 4.636474 | 0.00012 | 0.000862 |
| ESPL1 | 4.219988 | 0.000361 | 0.002178 |
| ESYT3 | -2.38724 | 1.04E-06 | 1.54E-05 |
| ETV1 | 2.973948 | 1.02E-05 | 0.000108 |
| ETV4 | 3.548884 | 0.013732 | 0.042023 |
| EXD1 | -3.45043 | 5.68E-11 | 4.27E-09 |
| EXO1 | 3.332551 | 0.00323 | 0.013297 |
| EXOC3L2 | 3.535908 | 0.016305 | 0.04806 |
| EXTL1 | -2.26253 | 4.81E-07 | 7.95E-06 |
| EYA1 | 2.838916 | 0.004292 | 0.016766 |
| EZH2 | 4.178713 | 1.15E-07 | 2.43E-06 |
| F2R | 4.297242 | 2.14E-11 | 2.03E-09 |
| F2RL3 | 3.267586 | 0.005297 | 0.01983 |
| FABP3 | -2.6784 | 2.63E-12 | 4.2E-10 |
| FADS6 | -3.20998 | 1.43E-05 | 0.000145 |
| FAIM2 | -2.02796 | 9.33E-08 | 2.03E-06 |
| FAIM3 | 2.151224 | 0.003467 | 0.014066 |
| FAM111B | 4.9025 | 6.37E-05 | 0.00051 |
| FAM119B | 2.955521 | 0.00451 | 0.017416 |
| FAM129A | 2.102069 | 0.016598 | 0.048724 |
| FAM153A | -3.90185 | 4.44E-12 | 6.25E-10 |
| FAM153B | -4.52474 | 6E-13 | 1.28E-10 |
| FAM153C | -4.17055 | 6.95E-08 | 1.58E-06 |
| FAM163B | -3.15415 | 5.13E-08 | 1.24E-06 |
| FAM176A | 2.773543 | 0.008525 | 0.028956 |
| FAM189A1 | -2.41548 | 8.97E-09 | 2.82E-07 |
| FAM19A1 | -3.06217 | 9.6E-08 | 2.08E-06 |
| FAM19A2 | -2.32007 | 1.77E-05 | 0.000174 |
| FAM19A4 | -2.4652 | 2.52E-06 | 3.31E-05 |
| FAM46A | 2.301432 | 0.001156 | 0.005719 |
| FAM50B | -2.01348 | 3.34E-07 | 5.89E-06 |
| FAM54A | 2.890956 | 0.000792 | 0.004186 |
| FAM60A | 3.697135 | 4.61E-10 | 2.34E-08 |
| FAM64A | 6.180569 | 1.73E-05 | 0.00017 |
| FAM65B | -2.01541 | 2.74E-07 | 5.01E-06 |
| FAM75A2 | -2.3702 | 0.003244 | 0.013329 |
| FAM75C1 | -2.13922 | 0.001754 | 0.008082 |
| FAM84B | 2.27592 | 0.000763 | 0.004057 |
| FANCD2 | 2.707839 | 0.000203 | 0.001338 |
| FANCI | 2.459709 | 0.000777 | 0.004109 |
| FBN2 | 3.8699 | 0.000749 | 0.003998 |
| FBXL16 | -2.52905 | 2.34E-08 | 6.42E-07 |
| FBXL2 | -2.00907 | 6.28E-08 | 1.46E-06 |
| FBXL21 | -2.56159 | 0.000117 | 0.000847 |
| FBXO40 | -3.04449 | 6.18E-07 | 9.94E-06 |
| FBXO41 | -2.22593 | 1.29E-08 | 3.84E-07 |
| FBXO43 | 2.503504 | 0.001043 | 0.005273 |
| FBXW10 | 2.314899 | 0.005435 | 0.020252 |
| FBXW7 | -2.27207 | 1.1E-12 | 2.08E-10 |
| FCER1G | 2.052553 | 0.006713 | 0.023969 |
| FCER2 | -3.90454 | 3.71E-08 | 9.36E-07 |
| FCHO1 | -2.38501 | 3.49E-13 | 8.05E-11 |
| FER1L6 | -3.18554 | 1.35E-07 | 2.76E-06 |
| FERMT1 | 3.989729 | 0.000238 | 0.001531 |
| FEZF2 | -2.5275 | 2.84E-06 | 3.65E-05 |
| FFAR1 | -2.95792 | 9.88E-06 | 0.000105 |
| FGF22 | -2.90209 | 1E-07 | 2.15E-06 |
| FGFBP2 | 4.35459 | 0.016792 | 0.049182 |
| FGFBP3 | 2.074142 | 0.004828 | 0.018383 |
| FHDC1 | 3.436827 | 8.91E-05 | 0.000674 |
| FKBP10 | 2.123341 | 0.000489 | 0.00281 |
| FKBP7 | 2.114824 | 2.96E-07 | 5.34E-06 |
| FLJ16779 | 3.010212 | 0.003118 | 0.012916 |
| FLJ32063 | -2.39844 | 1.11E-05 | 0.000117 |
| FLJ35390 | -2.39688 | 3.12E-10 | 1.71E-08 |
| FLJ37307 | -3.46729 | 1.19E-15 | 1.09E-12 |
| FLJ40330 | -2.83648 | 1.84E-08 | 5.2E-07 |
| FLJ40852 | 2.147329 | 1.66E-06 | 2.31E-05 |
| FLVCR2 | 2.546986 | 2.27E-05 | 0.000214 |
| FN1 | 2.498469 | 0.005893 | 0.021603 |
| FOLR1 | 3.022594 | 0.014142 | 0.04304 |
| FOXD1 | 3.007549 | 0.000443 | 0.002588 |
| FOXD3 | 5.864395 | 0.001651 | 0.007692 |
| FOXD4L6 | -2.46106 | 4.62E-10 | 2.34E-08 |
| FOXJ1 | 3.719047 | 0.002317 | 0.010138 |
| FOXM1 | 4.238256 | 9.45E-05 | 0.00071 |
| FOXN4 | 2.651035 | 0.015553 | 0.046361 |
| FPR3 | 3.008208 | 0.00642 | 0.023155 |
| FRMPD2L1 | -2.45274 | 0.011097 | 0.035681 |
| FRMPD4 | -3.25555 | 1.03E-07 | 2.2E-06 |
| FRRS1 | 3.620337 | 0.002634 | 0.011279 |
| FSTL4 | -2.85621 | 8.35E-07 | 1.28E-05 |
| FUT1 | -2.14293 | 7.22E-11 | 5.11E-09 |
| FXYD2 | 2.279472 | 0.004573 | 0.017636 |
| FXYD4 | -2.39461 | 0.00287 | 0.012082 |
| FXYD7 | -2.27628 | 0.000124 | 0.00089 |
| FZD7 | 2.088026 | 0.006446 | 0.023227 |
| GABARAPL1 | -2.00458 | 3.69E-13 | 8.41E-11 |
| GABRA1 | -3.17691 | 2.14E-06 | 2.86E-05 |
| GABRA2 | -3.04809 | 1.25E-07 | 2.59E-06 |
| GABRA4 | -3.08337 | 8.69E-07 | 1.33E-05 |
| GABRA5 | -3.79733 | 6.57E-11 | 4.72E-09 |
| GABRB2 | -2.92913 | 5.63E-06 | 6.53E-05 |
| GABRD | -2.2332 | 6.81E-05 | 0.000539 |
| GABRG2 | -2.65191 | 4.95E-05 | 0.000412 |
| GABRG3 | -3.22051 | 1.84E-06 | 2.52E-05 |
| GAD2 | -3.01764 | 2.25E-06 | 0.00003 |
| GADD45G | 2.353822 | 0.00395 | 0.015642 |
| GALNT6 | -2.3207 | 5.58E-08 | 1.32E-06 |
| GALNTL5 | -3.08009 | 1.26E-05 | 0.00013 |
| GALNTL6 | -3.4157 | 2.34E-09 | 9.05E-08 |
| GAPT | 2.843345 | 0.004897 | 0.018586 |
| GAS7 | -2.17924 | 2.95E-09 | 1.1E-07 |
| GBP1 | 2.602325 | 0.009215 | 0.030739 |
| GBP2 | 2.833377 | 0.003252 | 0.013354 |
| GBP3 | 2.492339 | 0.005987 | 0.021895 |
| GBP7 | 4.039292 | 0.014481 | 0.043898 |
| GBX2 | 5.235181 | 5.28E-05 | 0.000435 |
| GCNT4 | -2.17726 | 2.14E-07 | 4.05E-06 |
| GDA | -3.42773 | 7.63E-09 | 2.47E-07 |
| GFI1B | 3.594358 | 0.009658 | 0.031917 |
| GFOD1 | -2.43764 | 1.83E-10 | 1.11E-08 |
| GGNBP1 | -2.07378 | 0.005619 | 0.020797 |
| GINS2 | 2.417809 | 0.002006 | 0.009008 |
| GIPR | -2.17632 | 2.19E-05 | 0.000209 |
| GJB6 | -2.55542 | 0.000901 | 0.004682 |
| GJC2 | -2.26572 | 2.97E-06 | 0.000038 |
| GJD4 | -2.67728 | 5.5E-06 | 6.41E-05 |
| GLB1L3 | -2.42281 | 2.57E-06 | 3.35E-05 |
| GLDN | -2.01522 | 2.05E-06 | 2.76E-05 |
| GLP2R | -4.67094 | 7.33E-17 | 2.17E-13 |
| GLRA2 | -2.75753 | 1.27E-05 | 0.000131 |
| GLS | -2.31903 | 9.94E-16 | 1.04E-12 |
| GLS2 | -2.82685 | 1.18E-07 | 2.48E-06 |
| GLT1D1 | -3.15641 | 1.78E-09 | 7.24E-08 |
| GMNN | 2.113358 | 1.18E-05 | 0.000123 |
| GNASAS | 2.355825 | 0.003374 | 0.013762 |
| GNG13 | -2.86391 | 3.63E-05 | 0.000319 |
| GNG3 | -2.86263 | 5.42E-07 | 8.83E-06 |
| GOLGA6L1 | -3.39786 | 0.000126 | 0.000898 |
| GOLGA6L6 | -3.41294 | 1.69E-05 | 0.000167 |
| GOT1 | -2.28469 | 4.03E-12 | 5.86E-10 |
| GPC2 | 2.692951 | 7.07E-05 | 0.000554 |
| GPR12 | -2.30393 | 3.94E-05 | 0.000342 |
| GPR149 | -2.51271 | 0.00081 | 0.004264 |
| GPR150 | -3.3805 | 5.95E-10 | 2.88E-08 |
| GPR21 | -2.13303 | 0.000122 | 0.000876 |
| GPR22 | -3.15382 | 3.88E-07 | 6.6E-06 |
| GPR26 | -2.45828 | 0.002491 | 0.010759 |
| GPR37 | -2.20393 | 1.42E-06 | 2.01E-05 |
| GPR52 | -3.90155 | 9.05E-08 | 1.99E-06 |
| GPR6 | -3.63565 | 8.43E-08 | 1.88E-06 |
| GPR61 | -2.68158 | 1.14E-07 | 2.41E-06 |
| GPR62 | -2.51719 | 1.45E-06 | 2.06E-05 |
| GPR65 | 3.186151 | 0.002777 | 0.011766 |
| GPR68 | -3.15816 | 2.33E-13 | 5.75E-11 |
| GPR78 | -3.47734 | 1.07E-06 | 1.58E-05 |
| GPR82 | 5.011328 | 0.001218 | 0.005985 |
| GPR83 | -3.51312 | 1.07E-10 | 7.13E-09 |
| GPRASP1 | -2.64812 | 6.33E-13 | 1.32E-10 |
| GPRIN2 | -2.46174 | 3.37E-06 | 4.22E-05 |
| GPX3 | 3.500986 | 0.001334 | 0.006445 |
| GPX7 | 2.039446 | 0.000814 | 0.004282 |
| GPX8 | 3.407825 | 0.016931 | 0.049481 |
| GREM2 | -3.23464 | 2.8E-10 | 1.56E-08 |
| GRHL3 | 3.101792 | 0.001137 | 0.005648 |
| GRIN1 | -3.2523 | 1.91E-07 | 3.7E-06 |
| GRIN2A | -2.87598 | 3.79E-07 | 6.48E-06 |
| GRIN2B | -3.20237 | 1.03E-07 | 2.21E-06 |
| GRIP2 | -2.075 | 0.000513 | 0.002924 |
| GRM1 | -2.85968 | 2.27E-09 | 8.81E-08 |
| GRM2 | -3.0443 | 1.08E-09 | 4.88E-08 |
| GRM3 | -2.62421 | 1.49E-08 | 4.31E-07 |
| GRM4 | -2.82085 | 1.6E-06 | 2.24E-05 |
| GRM5 | -2.30147 | 7.99E-05 | 0.000616 |
| GRM7 | -3.08543 | 8.49E-08 | 1.88E-06 |
| GSC | 6.587514 | 3.5E-07 | 6.11E-06 |
| GSG2 | 3.122965 | 0.001452 | 0.006896 |
| GSX1 | 5.21838 | 0.000702 | 0.003795 |
| GTSE1 | 4.609907 | 3.95E-05 | 0.000342 |
| GUCA1A | -2.66251 | 6.85E-05 | 0.000541 |
| GUCY1B3 | -2.38313 | 1.51E-11 | 1.57E-09 |
| HAND2 | 7.200821 | 0.008143 | 0.027943 |
| HAPLN1 | 2.30628 | 0.015519 | 0.046308 |
| HAPLN2 | -2.01086 | 0.000364 | 0.002196 |
| HAS1 | -2.56474 | 2.78E-05 | 0.000255 |
| HAS2 | 3.203413 | 0.000464 | 0.0027 |
| HAS2AS | 2.63101 | 0.000028 | 0.000256 |
| HBG1 | -3.8365 | 9.28E-11 | 6.28E-09 |
| HBG2 | -3.04567 | 1.37E-05 | 0.00014 |
| HBM | -2.42344 | 0.003497 | 0.014163 |
| HBQ1 | -2.41965 | 0.00002 | 0.000193 |
| HCN1 | -3.09366 | 2.38E-06 | 3.14E-05 |
| HCRTR2 | -2.66376 | 8.32E-05 | 0.000638 |
| HECW1 | -2.26393 | 0.000035 | 0.000309 |
| HELLS | 3.271687 | 0.000105 | 0.000776 |
| HES6 | 3.204089 | 2.21E-05 | 0.00021 |
| HEY1 | 2.277483 | 3.79E-07 | 6.48E-06 |
| HHIP | -2.22781 | 7.65E-05 | 0.000594 |
| HIPK4 | -3.41197 | 4.48E-09 | 1.61E-07 |
| HIST1H4J | 2.778892 | 0.001501 | 0.007078 |
| HJURP | 5.308766 | 0.000046 | 0.000388 |
| HKDC1 | 2.588953 | 0.003668 | 0.014719 |
| HLA-DMB | 2.097539 | 0.008562 | 0.029048 |
| HLA-DQA2 | 3.992789 | 0.007749 | 0.026886 |
| HMCN1 | 2.733861 | 0.000557 | 0.00313 |
| HMMR | 3.812327 | 0.000387 | 0.002308 |
| HMX1 | 3.649217 | 0.005172 | 0.019467 |
| HNF4G | 2.688214 | 0.00011 | 0.000805 |
| HOOK1 | -3.1686 | 7.24E-09 | 2.36E-07 |
| HOXA1 | 5.561188 | 0.002398 | 0.010427 |
| HOXA10 | 8.042988 | 0.003808 | 0.015198 |
| HOXA11 | 9.070721 | 0.005558 | 0.020622 |
| HOXA13 | 6.356357 | 0.014948 | 0.044968 |
| HOXA4 | 7.566468 | 0.014436 | 0.04379 |
| HOXA5 | 9.47707 | 0.009098 | 0.030447 |
| HOXA7 | 9.80145 | 0.003766 | 0.015054 |
| HOXB2 | 4.940352 | 0.008608 | 0.029153 |
| HOXB3 | 6.202223 | 0.008249 | 0.028248 |
| HOXB4 | 7.337983 | 0.004994 | 0.018928 |
| HOXB6 | 5.632475 | 0.008479 | 0.028842 |
| HOXB7 | 6.556919 | 0.000321 | 0.001972 |
| HOXC4 | 3.948441 | 0.001403 | 0.006707 |
| HOXC5 | 7.537055 | 0.010165 | 0.03332 |
| HOXD1 | -2.04215 | 0.000381 | 0.002277 |
| HOXD10 | 9.793513 | 0.005493 | 0.020432 |
| HOXD4 | 6.474595 | 0.000825 | 0.004332 |
| HOXD8 | 7.563739 | 0.000305 | 0.00189 |
| HOXD9 | 8.615461 | 0.002186 | 0.009657 |
| HP | 4.301122 | 0.007367 | 0.025808 |
| HPCA | -2.96733 | 2.09E-07 | 4.01E-06 |
| HPCAL4 | -2.82339 | 2.4E-08 | 6.55E-07 |
| HPD | 6.51508 | 0.005695 | 0.021016 |
| HPN | -2.14172 | 0.000062 | 0.000498 |
| HPRT1 | -2.17601 | 5.85E-11 | 4.32E-09 |
| HPSE | 2.186124 | 0.004254 | 0.016628 |
| HRH2 | -2.59575 | 5.54E-08 | 1.32E-06 |
| HRH3 | -2.21444 | 0.000522 | 0.002969 |
| HRNBP3 | -3.01311 | 8.04E-07 | 1.25E-05 |
| HS3ST2 | -3.63178 | 2.11E-15 | 1.63E-12 |
| HS3ST4 | -2.68742 | 8.59E-07 | 1.32E-05 |
| HS3ST5 | -2.33824 | 6.68E-07 | 1.06E-05 |
| HS6ST3 | -3.09019 | 8.42E-08 | 1.88E-06 |
| HSPA12A | -2.38842 | 6.76E-10 | 3.2E-08 |
| HSPB3 | -2.65669 | 2.01E-05 | 0.000194 |
| HTR1A | -2.11302 | 0.006897 | 0.024513 |
| HTR1B | -2.04597 | 0.00047 | 0.002724 |
| HTR1E | -3.25806 | 1.77E-06 | 2.43E-05 |
| HTR2A | -2.99819 | 8.22E-09 | 2.62E-07 |
| HTR3A | -3.57689 | 4.46E-08 | 1.1E-06 |
| HTR3B | -3.54546 | 1.24E-05 | 0.000128 |
| HTR4 | -2.83416 | 4.32E-06 | 5.23E-05 |
| HTR5A | -3.06498 | 3.36E-06 | 4.22E-05 |
| HTR6 | -2.56817 | 6.49E-05 | 0.000518 |
| HTR7 | -2.29554 | 9.71E-08 | 2.1E-06 |
| HYMAI | -2.06759 | 0.000213 | 0.001392 |
| ICAM5 | -3.69875 | 1.05E-13 | 3.04E-11 |
| ID3 | 3.064874 | 1.41E-05 | 0.000143 |
| ID4 | 2.725617 | 6.1E-06 | 6.98E-05 |
| IDH1 | 2.00911 | 1.06E-07 | 2.25E-06 |
| IDS | -2.10382 | 1.77E-09 | 7.21E-08 |
| IFI30 | 2.60174 | 0.011466 | 0.036674 |
| IFNA21 | -2.20423 | 0.000093 | 0.0007 |
| IGF2BP3 | 4.177144 | 0.006955 | 0.024677 |
| IGFBP1 | 2.417403 | 0.011641 | 0.037116 |
| IGFBP2 | 4.243385 | 0.00786 | 0.027184 |
| IGFBP3 | 3.081625 | 0.012871 | 0.040045 |
| IGFBP7 | 2.16972 | 0.001361 | 0.006549 |
| IGSF10 | -2.04516 | 1.17E-06 | 1.72E-05 |
| IGSF22 | -2.13104 | 7.1E-16 | 8.4E-13 |
| IGSF5 | 2.475634 | 8.74E-06 | 9.51E-05 |
| IKBIP | 2.032239 | 0.000726 | 0.003899 |
| IL12RB2 | -3.84806 | 1.22E-15 | 1.09E-12 |
| IL17RD | 2.044955 | 1.65E-06 | 2.29E-05 |
| IL1R2 | 4.557211 | 0.011446 | 0.03663 |
| IL1RAPL2 | -3.33323 | 8.7E-07 | 1.33E-05 |
| IL1RL2 | -2.83002 | 2.27E-06 | 3.01E-05 |
| IL34 | -2.53636 | 2.33E-10 | 1.34E-08 |
| IMP5 | -2.45072 | 1.93E-07 | 3.74E-06 |
| INPP5F | -2.08692 | 4.79E-10 | 2.4E-08 |
| INPP5J | -2.09977 | 1.95E-08 | 5.45E-07 |
| IPCEF1 | -2.48632 | 2.21E-07 | 4.16E-06 |
| IQGAP2 | 4.272812 | 0.000045 | 0.00038 |
| IQGAP3 | 2.466184 | 0.01559 | 0.046428 |
| IQSEC3 | -2.52293 | 1.92E-05 | 0.000186 |
| IRX1 | 6.003032 | 0.000624 | 0.003452 |
| IRX2 | 5.165524 | 0.003325 | 0.013614 |
| IRX3 | 5.17662 | 6.57E-06 | 7.43E-05 |
| IRX5 | 4.768284 | 0.004337 | 0.016894 |
| ISLR2 | -3.70124 | 2.9E-15 | 2.04E-12 |
| ISM2 | -2.2737 | 7.64E-06 | 8.46E-05 |
| ITPKA | -2.7902 | 4.62E-11 | 3.69E-09 |
| ITPR1 | -2.35021 | 2.88E-07 | 5.23E-06 |
| ITPRIPL1 | 2.245193 | 0.005565 | 0.020641 |
| JAKMIP1 | -2.63924 | 5.23E-08 | 1.26E-06 |
| JPH1 | -2.48392 | 2.88E-07 | 5.23E-06 |
| JUB | 2.665121 | 0.000038 | 0.000332 |
| KALRN | -2.21754 | 2.25E-10 | 1.31E-08 |
| KCNA1 | -2.46522 | 3.04E-05 | 0.000274 |
| KCNA3 | -2.4085 | 8.28E-07 | 1.28E-05 |
| KCNA4 | -3.03927 | 2.21E-08 | 6.12E-07 |
| KCNAB1 | -2.04763 | 6.46E-06 | 7.34E-05 |
| KCNAB2 | -2.83821 | 2.25E-11 | 2.12E-09 |
| KCNB2 | -2.95581 | 6.19E-06 | 7.08E-05 |
| KCNC1 | -2.01509 | 2.35E-06 | 3.11E-05 |
| KCNC2 | -3.05562 | 1.97E-06 | 2.67E-05 |
| KCNC3 | -2.31211 | 4.92E-12 | 6.62E-10 |
| KCNC4 | -2.25458 | 1.29E-09 | 5.67E-08 |
| KCNG3 | -3.4156 | 4.59E-08 | 1.12E-06 |
| KCNH3 | -2.9374 | 2.5E-09 | 9.54E-08 |
| KCNH5 | -2.59825 | 0.000523 | 0.002969 |
| KCNIP4 | -2.38946 | 1.1E-07 | 2.33E-06 |
| KCNJ1 | -2.68628 | 1.47E-09 | 6.24E-08 |
| KCNJ12 | -2.505 | 1.15E-06 | 1.68E-05 |
| KCNJ3 | -2.80134 | 8.24E-07 | 1.28E-05 |
| KCNJ4 | -3.1999 | 6.37E-10 | 3.04E-08 |
| KCNJ6 | -2.21344 | 5.56E-05 | 0.000454 |
| KCNJ9 | -2.04053 | 3.76E-05 | 0.000329 |
| KCNK1 | -2.86429 | 3.4E-10 | 1.81E-08 |
| KCNK9 | -2.87512 | 5.85E-07 | 9.47E-06 |
| KCNMA1 | -2.07336 | 9.96E-10 | 4.53E-08 |
| KCNMB1 | 2.12996 | 0.004837 | 0.018409 |
| KCNN1 | -2.07103 | 1.95E-06 | 2.65E-05 |
| KCNQ3 | -2.53094 | 3.16E-09 | 1.18E-07 |
| KCNS1 | -2.48428 | 0.000703 | 0.0038 |
| KCNS2 | -3.04421 | 4.13E-06 | 5.04E-05 |
| KCNT1 | -2.96499 | 1.38E-06 | 1.97E-05 |
| KCNV1 | -3.33968 | 8.3E-06 | 0.000091 |
| KCTD16 | -2.49689 | 2.09E-08 | 5.79E-07 |
| KCTD4 | -2.33537 | 9.34E-07 | 1.41E-05 |
| KCTD8 | -2.15208 | 4.34E-07 | 7.27E-06 |
| KERA | -3.19504 | 0.00225 | 0.009901 |
| KIAA0040 | 2.169325 | 0.016198 | 0.047845 |
| KIAA0101 | 4.981316 | 0.000105 | 0.000774 |
| KIAA0284 | -2.55203 | 1.11E-13 | 3.19E-11 |
| KIAA0319 | -2.74416 | 1.69E-08 | 4.82E-07 |
| KIAA0513 | -2.91684 | 4.11E-16 | 6.64E-13 |
| KIAA0748 | -2.78462 | 0.000378 | 0.002263 |
| KIAA1045 | -3.22808 | 2.36E-09 | 9.09E-08 |
| KIAA1107 | -2.3345 | 2.72E-09 | 1.03E-07 |
| KIAA1239 | -3.53897 | 8.77E-08 | 1.94E-06 |
| KIAA1324 | -2.70041 | 4.5E-10 | 2.3E-08 |
| KIAA1486 | -2.22566 | 0.000962 | 0.004935 |
| KIAA1644 | -2.559 | 7.94E-06 | 8.76E-05 |
| KIAA1804 | -2.32549 | 2.77E-07 | 5.06E-06 |
| KIF11 | 2.628458 | 0.000178 | 0.001199 |
| KIF12 | -3.93684 | 2.25E-08 | 6.21E-07 |
| KIF14 | 4.973899 | 2.39E-05 | 0.000224 |
| KIF15 | 3.649728 | 1.38E-05 | 0.000141 |
| KIF17 | -2.26015 | 4.79E-09 | 1.69E-07 |
| KIF18A | 4.105509 | 0.000415 | 0.002446 |
| KIF18B | 3.615331 | 6.33E-05 | 0.000507 |
| KIF20A | 6.047909 | 6.11E-05 | 0.000492 |
| KIF23 | 3.653635 | 0.001935 | 0.008737 |
| KIF2C | 4.416681 | 0.000177 | 0.001194 |
| KIF4A | 4.836583 | 2.75E-05 | 0.000253 |
| KIF5A | -2.04671 | 0.000108 | 0.000793 |
| KIFC1 | 5.079907 | 7.02E-06 | 7.86E-05 |
| KIRREL3 | -2.79389 | 5.37E-10 | 2.64E-08 |
| KL | -2.06961 | 2.69E-05 | 0.000248 |
| KLHDC8A | 2.433432 | 0.006352 | 0.022958 |
| KLHL1 | -4.16049 | 1.83E-11 | 1.82E-09 |
| KLHL14 | -2.07085 | 0.001288 | 0.006259 |
| KLHL3 | -2.0066 | 9.05E-10 | 4.15E-08 |
| KLHL34 | -2.82486 | 6.7E-09 | 2.23E-07 |
| KLHL38 | -2.54481 | 1.71E-07 | 3.36E-06 |
| KLK10 | -2.55081 | 1.22E-06 | 1.77E-05 |
| KLK5 | -3.20183 | 4.49E-05 | 0.000379 |
| KLK7 | -4.03809 | 3.57E-09 | 1.3E-07 |
| KLRC2 | 3.759415 | 0.014084 | 0.04288 |
| KLRK1 | 3.109602 | 0.001886 | 0.008559 |
| KNCN | -2.63948 | 0.000679 | 0.003698 |
| KNDC1 | -2.19772 | 3.92E-07 | 6.65E-06 |
| KRT222 | -2.30591 | 6.09E-10 | 2.94E-08 |
| KRT31 | -3.00471 | 0.000114 | 0.00083 |
| KRT33B | -3.46214 | 1.58E-05 | 0.000157 |
| KRT81 | -2.40952 | 1.52E-05 | 0.000152 |
| KRTAP5-2 | -3.05201 | 3.27E-06 | 4.13E-05 |
| LAG3 | 2.601946 | 0.001991 | 0.008959 |
| LAMB4 | 2.669473 | 0.007772 | 0.026936 |
| LAMP3 | 2.362965 | 0.016971 | 0.049574 |
| LBX2 | 3.631098 | 0.001794 | 0.008228 |
| LCAT | 2.205051 | 0.000136 | 0.000958 |
| LCE1E | 5.637478 | 0.006524 | 0.023439 |
| LCN15 | -3.58197 | 1.34E-06 | 1.91E-05 |
| LCN8 | -2.00893 | 0.002785 | 0.011785 |
| LDB3 | -2.45753 | 1.06E-06 | 1.57E-05 |
| LDHAL6A | -2.25572 | 3.62E-07 | 6.28E-06 |
| LEFTY2 | 4.305023 | 0.007758 | 0.02691 |
| LFNG | 2.642116 | 0.00007 | 0.000551 |
| LGI3 | -2.51615 | 2.3E-07 | 4.29E-06 |
| LHFPL3 | 2.769578 | 0.001087 | 0.005454 |
| LHFPL5 | -3.2959 | 4.49E-07 | 7.46E-06 |
| LHX6 | -3.10554 | 2.32E-10 | 1.34E-08 |
| LHX9 | 5.73037 | 0.001626 | 0.00759 |
| LIMA1 | 2.587328 | 3.48E-08 | 8.89E-07 |
| LINGO2 | -2.92717 | 8.97E-06 | 9.71E-05 |
| LIPH | -2.89137 | 1.34E-07 | 2.74E-06 |
| LMNB1 | 2.889716 | 3.63E-05 | 0.000319 |
| LMO7 | -2.3275 | 3.1E-10 | 1.71E-08 |
| LONRF2 | -2.2517 | 1.62E-11 | 1.66E-09 |
| LOXHD1 | -3.98112 | 8E-11 | 5.59E-09 |
| LOXL2 | 2.910167 | 0.004665 | 0.017904 |
| LPA | -3.21174 | 1.22E-07 | 2.54E-06 |
| LPAR3 | -3.35637 | 7.15E-06 | 7.98E-05 |
| LPL | 4.201172 | 4.96E-06 | 5.89E-05 |
| LPO | -2.11267 | 1.69E-06 | 2.33E-05 |
| LPPR4 | -2.45535 | 2.11E-11 | 2.02E-09 |
| LRFN2 | -3.09857 | 6.33E-11 | 4.57E-09 |
| LRFN5 | -2.37367 | 2.61E-05 | 0.000241 |
| LRP2 | -2.5332 | 2.84E-05 | 0.00026 |
| LRRC10 | 4.32842 | 0.00663 | 0.023722 |
| LRRC17 | 2.469212 | 0.002788 | 0.011793 |
| LRRC46 | 2.243744 | 0.004588 | 0.01767 |
| LRRC67 | 2.080039 | 0.012819 | 0.039952 |
| LRRC7 | -3.1138 | 6.2E-11 | 4.49E-09 |
| LRRN1 | 2.14176 | 0.000184 | 0.001232 |
| LRTM1 | -2.02153 | 0.005465 | 0.020346 |
| LRTM2 | -2.75687 | 1.04E-05 | 0.00011 |
| LY6H | -2.39921 | 1.49E-06 | 0.000021 |
| LYPD6B | -2.58856 | 2.93E-05 | 0.000266 |
| LYZL4 | -2.88983 | 9.93E-05 | 0.00074 |
| MACROD2 | -2.10704 | 8.76E-07 | 1.33E-05 |
| MAG | -2.37166 | 0.000121 | 0.000872 |
| MAGEE1 | -2.32013 | 1.09E-09 | 4.91E-08 |
| MAL | -2.50948 | 7.52E-06 | 8.35E-05 |
| MAL2 | -3.43698 | 2.8E-08 | 7.48E-07 |
| MAML2 | 2.963689 | 9.57E-07 | 1.44E-05 |
| MAP3K10 | -2.0621 | 3.8E-12 | 5.57E-10 |
| MAP3K9 | -2.65099 | 7.95E-08 | 1.78E-06 |
| MAP7D2 | -3.20659 | 1.21E-07 | 2.51E-06 |
| MAPK15 | -2.32225 | 0.005567 | 0.020641 |
| MAPRE3 | -2.0293 | 1.79E-16 | 3.85E-13 |
| MARVELD3 | 4.55668 | 0.000209 | 0.001372 |
| MAS1 | -2.90877 | 0.000663 | 0.003629 |
| MASP1 | 3.20502 | 3.05E-06 | 3.89E-05 |
| MAST3 | -2.78985 | 2.21E-17 | 7.83E-14 |
| MATK | -2.56381 | 2.48E-07 | 4.58E-06 |
| MC4R | -2.31142 | 0.000295 | 0.001844 |
| MCHR2 | -3.58597 | 8.56E-06 | 9.35E-05 |
| MCM10 | 4.754436 | 0.000139 | 0.000971 |
| MCOLN2 | 2.940267 | 0.007333 | 0.025702 |
| MCTP1 | -2.57035 | 1.2E-08 | 3.6E-07 |
| MDFI | 4.302044 | 4.63E-07 | 7.66E-06 |
| MDK | 2.97712 | 0.001337 | 0.006457 |
| MEF2C | -2.24188 | 1.95E-11 | 1.9E-09 |
| MELK | 6.437947 | 2.54E-05 | 0.000235 |
| MEST | 2.461281 | 0.002164 | 0.009579 |
| MESTIT1 | 2.042954 | 0.001655 | 0.007698 |
| METTL7B | 4.226743 | 0.006543 | 0.02349 |
| MEX3A | 4.192672 | 1.34E-09 | 5.82E-08 |
| MFAP2 | 4.629638 | 0.009878 | 0.032509 |
| MFAP4 | 2.136671 | 0.014995 | 0.045087 |
| MFRP | 2.923972 | 1.55E-06 | 2.18E-05 |
| MFSD4 | -2.93353 | 2.38E-11 | 2.17E-09 |
| MGAT5B | -2.20906 | 1.24E-07 | 2.57E-06 |
| MGC12982 | 2.650474 | 0.003724 | 0.014909 |
| MIA | 4.206797 | 0.009878 | 0.032509 |
| MICAL2 | -2.98314 | 9.74E-11 | 6.53E-09 |
| MKI67 | 5.380206 | 6.22E-06 | 7.09E-05 |
| MKL2 | -2.07291 | 2.55E-12 | 4.15E-10 |
| MLF1IP | 3.966344 | 0.000113 | 0.000819 |
| MMP14 | 2.758255 | 0.001961 | 0.008836 |
| MMP2 | 3.002786 | 0.000119 | 0.00086 |
| MMP25 | 2.376734 | 0.001411 | 0.006743 |
| MMP3 | -2.43566 | 0.008331 | 0.028487 |
| MMP9 | 5.999936 | 0.014734 | 0.04449 |
| MNS1 | 2.133919 | 1.92E-05 | 0.000187 |
| MNX1 | 8.005857 | 0.000921 | 0.004763 |
| MPO | -2.1015 | 0.000196 | 0.001303 |
| MPP7 | -2.97014 | 1.16E-09 | 5.17E-08 |
| MPPED1 | -3.23377 | 2.92E-07 | 5.28E-06 |
| MRAP2 | -2.55022 | 1.79E-06 | 2.47E-05 |
| MS4A4A | 3.116455 | 0.000845 | 0.004422 |
| MS4A6A | 3.168086 | 0.001829 | 0.008352 |
| MS4A7 | 2.253479 | 0.001339 | 0.006461 |
| MS4A8B | -3.21774 | 3.18E-08 | 8.25E-07 |
| MSR1 | 2.666251 | 0.009202 | 0.030707 |
| MST1 | 2.016658 | 0.005852 | 0.021478 |
| MSTN | 4.012747 | 0.001493 | 0.007052 |
| MT1X | 2.010085 | 0.008874 | 0.029831 |
| MTHFD2 | 3.859347 | 2.95E-08 | 7.82E-07 |
| MTNR1A | -2.12925 | 0.00142 | 0.006775 |
| MTTP | 2.330052 | 0.014779 | 0.044608 |
| MTUS2 | -3.11081 | 4.95E-09 | 1.73E-07 |
| MUC1 | 2.043136 | 0.001271 | 0.006194 |
| MUM1L1 | -2.53941 | 9.05E-07 | 1.37E-05 |
| MYADML2 | -2.36334 | 1.02E-08 | 3.17E-07 |
| MYBL2 | 7.148768 | 2.43E-05 | 0.000227 |
| MYBPC2 | -2.29021 | 0.000297 | 0.00185 |
| MYC | 2.465639 | 0.000022 | 0.000209 |
| MYCL1 | 2.642589 | 0.000105 | 0.000774 |
| MYH7B | -2.09003 | 2.98E-05 | 0.00027 |
| MYO15A | -2.64217 | 1.05E-07 | 2.24E-06 |
| MYOM2 | -2.27011 | 1.14E-14 | 5.93E-12 |
| MYPN | -2.12981 | 0.000334 | 0.002041 |
| MYRIP | -2.11246 | 2.47E-08 | 6.68E-07 |
| MYT1 | 2.320028 | 0.016574 | 0.048679 |
| MYT1L | -2.8815 | 5.5E-06 | 6.41E-05 |
| NAP1L2 | -2.087 | 1.96E-06 | 2.66E-05 |
| NAPB | -2.94862 | 1.43E-11 | 1.51E-09 |
| NAT1 | 2.079619 | 0.001013 | 0.005155 |
| NCAPG | 5.750829 | 1.55E-05 | 0.000155 |
| NCAPH | 3.345899 | 0.000631 | 0.003484 |
| NCDN | -2.70789 | 1.32E-11 | 1.41E-09 |
| NCOA7 | -2.20155 | 2.94E-14 | 1.27E-11 |
| NCRNA00086 | -2.25217 | 1.97E-09 | 7.81E-08 |
| NCRNA00087 | -2.81752 | 5.07E-11 | 3.95E-09 |
| NCRNA00164 | -3.02256 | 5.32E-08 | 1.27E-06 |
| NCRNA00202 | -2.5359 | 1.33E-08 | 3.93E-07 |
| NCS1 | -2.4673 | 4.69E-12 | 6.41E-10 |
| NDC80 | 6.297813 | 6.52E-06 | 7.39E-05 |
| NDST3 | -2.15496 | 0.000904 | 0.004693 |
| NECAB1 | -2.58132 | 4.59E-09 | 1.64E-07 |
| NECAB2 | -2.74962 | 1.84E-10 | 1.11E-08 |
| NEDD4 | 2.300778 | 4.97E-06 | 5.89E-05 |
| NEFL | -3.07117 | 4.87E-06 | 5.82E-05 |
| NEFM | -2.88047 | 0.000042 | 0.00036 |
| NEGR1 | -2.25791 | 9.73E-07 | 1.46E-05 |
| NEIL3 | 3.185044 | 0.011062 | 0.035596 |
| NEK10 | -2.69756 | 1.04E-08 | 3.21E-07 |
| NELL1 | -3.10576 | 4.19E-07 | 7.07E-06 |
| NELL2 | -2.69811 | 1.44E-08 | 4.21E-07 |
| NES | 3.240769 | 1.2E-07 | 2.51E-06 |
| NEU4 | 2.733955 | 0.003007 | 0.012531 |
| NEURL | -2.93734 | 1.33E-08 | 3.92E-07 |
| NEUROD2 | -3.12985 | 3.18E-07 | 5.65E-06 |
| NEUROD6 | -3.48136 | 1.67E-06 | 2.31E-05 |
| NEXN | 2.260754 | 0.00053 | 0.003002 |
| NGB | -2.55207 | 0.00053 | 0.003002 |
| NGEF | -2.82012 | 3.6E-08 | 9.12E-07 |
| NID1 | 2.861463 | 0.000132 | 0.000932 |
| NID2 | 2.08098 | 0.01516 | 0.045491 |
| NIPAL2 | -2.0064 | 6.83E-05 | 0.00054 |
| NIPAL3 | -2.11676 | 2.8E-09 | 1.05E-07 |
| NIPAL4 | -2.08057 | 4.73E-05 | 0.000397 |
| NKAIN2 | -2.20723 | 4.13E-05 | 0.000354 |
| NKAIN4 | 2.506434 | 0.000792 | 0.004188 |
| NKD1 | 2.050913 | 0.002614 | 0.011206 |
| NKX2-5 | 8.115856 | 0.006966 | 0.024695 |
| NKX3-2 | 6.092281 | 0.000672 | 0.003665 |
| NKX6-2 | -2.28102 | 0.000131 | 0.000924 |
| NMB | 3.992628 | 0.000018 | 0.000177 |
| NOD2 | 2.103396 | 0.003158 | 0.013056 |
| NOS1AP | -2.32524 | 9.75E-11 | 6.53E-09 |
| NOX4 | 3.744852 | 0.003674 | 0.014742 |
| NPAS1 | -2.72882 | 4.51E-10 | 2.3E-08 |
| NPAS4 | -2.72132 | 0.001478 | 0.006994 |
| NPBWR1 | -2.84885 | 0.004701 | 0.018004 |
| NPFFR1 | -2.10322 | 0.00201 | 0.009022 |
| NPFFR2 | -2.0176 | 0.006981 | 0.024717 |
| NPHS1 | -3.11956 | 3.58E-07 | 6.23E-06 |
| NPM2 | -2.79873 | 5.14E-07 | 8.44E-06 |
| NPPC | -2.05063 | 0.002985 | 0.012465 |
| NPTX1 | -3.51108 | 7.14E-12 | 8.93E-10 |
| NPTX2 | -3.18129 | 2.74E-08 | 7.35E-07 |
| NPTXR | -2.77788 | 9.79E-15 | 5.56E-12 |
| NPY | -2.355 | 3.87E-05 | 0.000337 |
| NPY1R | -2.50448 | 3.43E-07 | 6.01E-06 |
| NPY5R | -2.61866 | 3.17E-05 | 0.000284 |
| NR5A2 | 3.822275 | 0.00033 | 0.002017 |
| NRAP | -3.42471 | 1.57E-09 | 6.61E-08 |
| NRG4 | -2.40869 | 3.59E-06 | 4.48E-05 |
| NRGN | -2.9479 | 1.25E-06 | 0.000018 |
| NRIP3 | -2.86657 | 7.08E-10 | 3.33E-08 |
| NRM | 2.561896 | 5.37E-06 | 6.29E-05 |
| NRXN3 | -2.93194 | 2.45E-10 | 1.4E-08 |
| NSF | -2.44395 | 1.82E-15 | 1.47E-12 |
| NT5DC2 | 2.117156 | 5.69E-06 | 6.58E-05 |
| NTN1 | 2.836261 | 1.82E-08 | 5.17E-07 |
| NTSR1 | -2.2033 | 0.001498 | 0.007071 |
| NUAK1 | -2.37222 | 1.45E-11 | 1.53E-09 |
| NUF2 | 3.870055 | 0.000156 | 0.001068 |
| NUPR1 | 2.175134 | 0.000258 | 0.001641 |
| NUSAP1 | 4.775893 | 1.85E-05 | 0.00018 |
| NXPH2 | -2.51107 | 8.27E-06 | 9.08E-05 |
| OCA2 | -2.54741 | 0.00189 | 0.008575 |
| ODF3B | 2.557551 | 0.006477 | 0.02332 |
| ODZ2 | -2.83795 | 2.36E-07 | 4.38E-06 |
| ODZ3 | -2.80186 | 4.15E-07 | 7.02E-06 |
| OGDHL | -2.47846 | 5.5E-06 | 6.41E-05 |
| OIP5 | 3.846425 | 0.000139 | 0.000975 |
| OLFM1 | -2.36338 | 1.94E-08 | 5.45E-07 |
| OLFM3 | -3.15652 | 1.65E-07 | 3.28E-06 |
| OPALIN | -3.20085 | 4.97E-06 | 5.89E-05 |
| OPCML | -2.01355 | 2.34E-05 | 0.00022 |
| OPN4 | -3.18864 | 8.29E-07 | 1.28E-05 |
| OPRD1 | -2.95133 | 8.12E-06 | 8.94E-05 |
| OPRK1 | -2.99114 | 1.53E-06 | 2.15E-05 |
| OPRM1 | -3.2219 | 5.09E-05 | 0.000422 |
| OR14I1 | -3.35828 | 2.28E-05 | 0.000215 |
| OR2W3 | -2.66341 | 2.27E-07 | 4.24E-06 |
| OR4N2 | 8.808856 | 0.001045 | 0.005283 |
| ORC1L | 3.673412 | 0.000615 | 0.003414 |
| OTOF | -3.14602 | 3.33E-09 | 1.23E-07 |
| OVOL2 | -2.49275 | 0.002254 | 0.009912 |
| OXGR1 | -2.28619 | 6.41E-05 | 0.000513 |
| P2RX2 | -2.3431 | 0.005538 | 0.020565 |
| P2RY8 | 2.199068 | 0.015473 | 0.046202 |
| PABPC1L2A | -2.7369 | 2.4E-06 | 3.16E-05 |
| PABPC1L2B | -2.95799 | 1.46E-07 | 2.94E-06 |
| PACSIN1 | -3.17087 | 7.05E-07 | 1.11E-05 |
| PAK1 | -2.92509 | 9.98E-15 | 5.56E-12 |
| PAK3 | -2.26172 | 5.72E-05 | 0.000466 |
| PAK6 | -3.12083 | 6.69E-12 | 8.48E-10 |
| PALM2 | -2.83972 | 4.2E-11 | 3.39E-09 |
| PAPL | -3.5276 | 9.59E-09 | 2.98E-07 |
| PAR4 | -2.69366 | 0.00007 | 0.000551 |
| PARM1 | -2.24958 | 1.02E-06 | 1.51E-05 |
| PART1 | -2.14356 | 4.45E-07 | 7.42E-06 |
| PAX1 | 6.374679 | 0.002335 | 0.010206 |
| PBK | 6.691535 | 3.05E-06 | 3.89E-05 |
| PCDH11Y | -2.50731 | 0.000183 | 0.001231 |
| PCDHAC2 | -2.236 | 5E-09 | 1.74E-07 |
| PCDHB9 | 2.300083 | 2.26E-05 | 0.000213 |
| PCDHGC5 | -2.61283 | 1.25E-11 | 1.34E-09 |
| PCLO | -3.23731 | 1.32E-10 | 8.4E-09 |
| PCP4L1 | -2.4272 | 3.94E-05 | 0.000342 |
| PCSK1 | -2.08382 | 0.000479 | 0.002758 |
| PDCD1LG2 | 3.025883 | 0.00439 | 0.017057 |
| PDE1A | -2.88768 | 2.69E-11 | 2.38E-09 |
| PDE1B | -2.74859 | 5.6E-11 | 4.23E-09 |
| PDE2A | -2.48375 | 3.13E-08 | 8.19E-07 |
| PDE4C | -2.99412 | 2.41E-11 | 2.19E-09 |
| PDE6B | -2.25979 | 7.17E-09 | 2.34E-07 |
| PDE6G | 2.277044 | 0.003343 | 0.013673 |
| PDGFRA | 2.427374 | 0.011052 | 0.035576 |
| PDIA2 | -2.25985 | 6.15E-06 | 7.04E-05 |
| PDP1 | -2.01196 | 3.09E-10 | 1.71E-08 |
| PDYN | -3.2794 | 1.81E-06 | 2.49E-05 |
| PDZD7 | -2.53019 | 7.71E-09 | 2.49E-07 |
| PEG3 | -2.0766 | 1.65E-06 | 0.000023 |
| PEX5L | -2.23322 | 3.25E-05 | 0.00029 |
| PGF | 3.180547 | 0.000554 | 0.003119 |
| PGM2L1 | -2.39086 | 8.81E-11 | 6.06E-09 |
| PHEX | 3.246767 | 0.001232 | 0.006044 |
| PHKG1 | 2.21608 | 0.003941 | 0.01561 |
| PHLDA1 | 2.589022 | 6.76E-06 | 7.61E-05 |
| PHYHIP | -2.94116 | 3.68E-08 | 9.3E-07 |
| PI4KA | -2.25212 | 9.46E-16 | 1.04E-12 |
| PIF1 | 2.648023 | 0.001363 | 0.006555 |
| PIP5K1B | -2.09017 | 8.09E-07 | 1.25E-05 |
| PITPNM3 | -2.72428 | 6.86E-09 | 2.26E-07 |
| PITX1 | 7.283202 | 0.000779 | 0.00412 |
| PKD2L1 | -3.24782 | 4.13E-11 | 3.38E-09 |
| PKMYT1 | 4.637811 | 3.82E-06 | 4.74E-05 |
| PKP1 | -2.03988 | 0.001058 | 0.005331 |
| PLA2G4D | -2.53479 | 9.66E-05 | 0.000724 |
| PLAC1 | 2.972848 | 0.009394 | 0.031205 |
| PLAC2 | -2.56481 | 2.68E-12 | 4.23E-10 |
| PLCH2 | -2.16539 | 1.49E-05 | 0.00015 |
| PLCXD3 | -2.09133 | 0.000189 | 0.001262 |
| PLEKHA1 | -2.00908 | 1E-13 | 2.97E-11 |
| PLEKHA4 | 2.593814 | 0.003525 | 0.014263 |
| PLEKHG2 | 2.367496 | 1.49E-06 | 0.000021 |
| PLEKHG5 | -2.03925 | 3.63E-10 | 1.92E-08 |
| PLK1 | 2.801364 | 0.000872 | 0.004547 |
| PLK2 | -2.35072 | 5.78E-10 | 2.81E-08 |
| PLLP | -2.06397 | 9.08E-07 | 1.37E-05 |
| PLP2 | 2.720318 | 0.008506 | 0.028919 |
| PNCK | -2.78559 | 6.93E-11 | 4.92E-09 |
| PNLDC1 | -2.32265 | 0.000714 | 0.003847 |
| PNMA3 | -2.27286 | 3.44E-06 | 4.31E-05 |
| PNMA5 | -3.6988 | 1.95E-12 | 3.35E-10 |
| PNMAL2 | -2.51721 | 2.9E-10 | 1.62E-08 |
| PNPLA5 | -3.04858 | 1.63E-05 | 0.000162 |
| POLE2 | 3.157545 | 0.002125 | 0.009443 |
| POLQ | 3.329257 | 0.00075 | 0.003998 |
| POM121L2 | -2.57015 | 2.08E-05 | 0.000199 |
| POM121L9P | 2.439948 | 0.010409 | 0.033942 |
| POPDC3 | -2.21413 | 1.83E-05 | 0.000179 |
| POU3F1 | -2.43787 | 1.27E-07 | 2.62E-06 |
| POU3F4 | 2.652859 | 0.002324 | 0.010162 |
| POU5F1B | 2.607924 | 0.000292 | 0.00183 |
| POU6F2 | -2.49576 | 0.000193 | 0.001282 |
| PP14571 | -2.37227 | 2.1E-10 | 1.24E-08 |
| PPEF1 | -2.66604 | 1.03E-06 | 1.53E-05 |
| PPFIA2 | -2.4686 | 1.46E-07 | 2.94E-06 |
| PPFIA3 | -2.37918 | 4.65E-12 | 6.39E-10 |
| PPFIA4 | -2.36959 | 8.27E-10 | 3.83E-08 |
| PPL | -2.55431 | 2.43E-05 | 0.000227 |
| PPM1H | -2.24556 | 2.05E-11 | 1.98E-09 |
| PPP1R14B | 2.328007 | 1.57E-07 | 3.17E-06 |
| PPP1R16B | -2.43729 | 5.73E-08 | 1.35E-06 |
| PPP2R2C | -2.64944 | 3.37E-07 | 5.93E-06 |
| PPP4R4 | -2.97027 | 3.51E-08 | 8.95E-07 |
| PRAP1 | -2.54428 | 5.36E-06 | 6.29E-05 |
| PRB2 | -2.8703 | 0.000103 | 0.000766 |
| PRDM8 | -2.19315 | 3.1E-06 | 3.94E-05 |
| PRKAR1B | -2.54269 | 7.28E-13 | 1.47E-10 |
| PRKCB | -2.72629 | 6.87E-09 | 2.26E-07 |
| PRKCE | -2.16776 | 1.87E-09 | 7.49E-08 |
| PRKCG | -3.72103 | 3.5E-10 | 1.86E-08 |
| PRKCZ | -2.40861 | 4.62E-10 | 2.34E-08 |
| PRKX | 2.194596 | 6.07E-05 | 0.00049 |
| PRMT8 | -3.06041 | 4.55E-08 | 1.12E-06 |
| PROKR2 | -3.28546 | 6.29E-06 | 7.15E-05 |
| PROS1 | 2.390179 | 0.000291 | 0.001825 |
| PRPH | 2.670815 | 0.001629 | 0.007602 |
| PRPH2 | -3.0484 | 1.7E-09 | 7E-08 |
| PRR11 | 2.339612 | 0.00203 | 0.009094 |
| PRR18 | -2.40734 | 6.74E-08 | 1.54E-06 |
| PRRG3 | -3.52382 | 8.59E-09 | 2.73E-07 |
| PRRT1 | -2.42068 | 5.49E-11 | 4.2E-09 |
| PRRT2 | -2.28841 | 1.73E-08 | 4.91E-07 |
| PRRX1 | 2.801763 | 2.86E-09 | 1.07E-07 |
| PRSS3 | -3.00187 | 1.43E-09 | 6.15E-08 |
| PRTG | 2.192553 | 0.000101 | 0.000749 |
| PSD | -2.17352 | 9.8E-06 | 0.000105 |
| PSD3 | -2.27544 | 9.45E-13 | 1.82E-10 |
| PSMB8 | 2.082522 | 0.000206 | 0.001351 |
| PSMB9 | 2.010294 | 0.001871 | 0.008497 |
| PSTPIP1 | 2.786184 | 0.000391 | 0.002329 |
| PTER | -3.47953 | 8.71E-13 | 1.72E-10 |
| PTGER3 | -3.29899 | 3.48E-09 | 1.27E-07 |
| PTH2R | -2.31791 | 0.000079 | 0.00061 |
| PTK2B | -2.10324 | 1.33E-09 | 5.8E-08 |
| PTK7 | 2.125365 | 6.76E-07 | 1.07E-05 |
| PTPN20B | -2.21743 | 0.000178 | 0.001199 |
| PTPN3 | -3.08123 | 4.19E-08 | 1.04E-06 |
| PTPN5 | -3.10231 | 3.57E-08 | 9.05E-07 |
| PTPRN | -2.66385 | 4.6E-07 | 7.62E-06 |
| PTPRR | -3.4234 | 5.24E-11 | 4.06E-09 |
| PTPRZ1 | 2.540053 | 1.58E-06 | 2.21E-05 |
| PTTG1 | 2.466598 | 0.011984 | 0.037941 |
| PXDN | 2.283095 | 0.000616 | 0.003414 |
| PYDC1 | -3.11702 | 1.41E-05 | 0.000143 |
| PYGL | 2.210437 | 0.002479 | 0.010724 |
| RAB11FIP4 | -2.19399 | 6.71E-08 | 1.53E-06 |
| RAB13 | 2.223052 | 9.75E-08 | 2.1E-06 |
| RAB15 | -2.37019 | 7.68E-12 | 9.33E-10 |
| RAB26 | -2.28517 | 2.14E-09 | 8.39E-08 |
| RAB3A | -2.93892 | 1.77E-10 | 1.08E-08 |
| RAB3B | -2.67582 | 9.4E-07 | 1.41E-05 |
| RAB3C | -2.04056 | 0.00035 | 0.002121 |
| RAB40B | -2.09117 | 1.75E-11 | 1.76E-09 |
| RAB42 | 3.451228 | 0.002068 | 0.009228 |
| RAD51 | 3.542157 | 0.000301 | 0.001871 |
| RAD51AP1 | 2.932438 | 0.000375 | 0.00225 |
| RAD51AP2 | -2.6178 | 4.14E-06 | 5.04E-05 |
| RALYL | -2.59331 | 2.22E-07 | 4.16E-06 |
| RAP1GAP | -2.34502 | 2.14E-11 | 2.03E-09 |
| RAP1GAP2 | -2.68045 | 1.17E-10 | 7.67E-09 |
| RAPGEF4 | -2.16846 | 8.38E-07 | 1.29E-05 |
| RAPGEF5 | -2.25555 | 1.33E-07 | 2.71E-06 |
| RAPGEFL1 | -2.11199 | 1.77E-09 | 7.21E-08 |
| RASAL1 | -4.04629 | 1.15E-13 | 3.23E-11 |
| RASD2 | -2.20384 | 1.08E-06 | 1.59E-05 |
| RASGEF1A | -2.5015 | 9.45E-08 | 2.05E-06 |
| RASGRF1 | -2.23971 | 8.61E-05 | 0.000656 |
| RASGRF2 | -2.66104 | 1.45E-08 | 4.21E-07 |
| RBM11 | -2.34267 | 5.03E-06 | 5.95E-05 |
| RBM24 | -2.34423 | 1.54E-05 | 0.000154 |
| RBM47 | 2.200518 | 0.003876 | 0.015403 |
| RBP4 | -3.14292 | 1.49E-08 | 4.31E-07 |
| RCAN2 | -2.53022 | 1.88E-08 | 5.31E-07 |
| RCC1 | 3.348428 | 4.13E-05 | 0.000354 |
| RCOR2 | 2.079957 | 0.006473 | 0.023311 |
| RELN | -2.4272 | 6.78E-05 | 0.000537 |
| RESP18 | -2.45272 | 0.002073 | 0.009245 |
| RFPL1 | -2.54605 | 0.000109 | 0.000798 |
| RFPL1S | -2.69132 | 5.85E-07 | 9.47E-06 |
| RFPL2 | -2.52667 | 7.65E-06 | 8.47E-05 |
| RFPL4B | 2.881885 | 0.013283 | 0.040944 |
| RGNEF | -2.61428 | 9.07E-10 | 4.15E-08 |
| RGS14 | -2.64632 | 6.35E-09 | 2.14E-07 |
| RGS4 | -3.07691 | 2.22E-07 | 4.17E-06 |
| RGS7 | -2.38966 | 1.01E-06 | 0.000015 |
| RGS7BP | -2.57651 | 2.21E-07 | 4.16E-06 |
| RGS8 | -2.00081 | 0.004017 | 0.015867 |
| RHCG | -2.35043 | 5.89E-05 | 0.000478 |
| RHO | -3.15621 | 6.61E-09 | 2.2E-07 |
| RHOF | -2.34038 | 5.89E-09 | 2.01E-07 |
| RHOV | -2.36269 | 5.54E-06 | 6.45E-05 |
| RIBC1 | 2.369084 | 0.003534 | 0.01429 |
| RICH2 | -2.72974 | 7.92E-09 | 2.54E-07 |
| RIMBP2 | -3.25016 | 1.13E-10 | 7.47E-09 |
| RIMS1 | -2.43376 | 6.34E-06 | 0.000072 |
| RIMS3 | -2.46414 | 1.23E-07 | 2.56E-06 |
| RLTPR | -2.65102 | 4.27E-07 | 7.18E-06 |
| RNASE10 | 4.188182 | 0.007021 | 0.024818 |
| RNASE13 | 2.162523 | 0.011278 | 0.036176 |
| RND2 | 2.74045 | 1.64E-06 | 2.28E-05 |
| RNF128 | -2.36477 | 0.000289 | 0.001814 |
| RNF175 | -2.08118 | 7.56E-06 | 8.38E-05 |
| RNF212 | -2.6793 | 8.92E-06 | 9.67E-05 |
| RP2 | 2.012656 | 1.18E-08 | 3.57E-07 |
| RPE65 | 4.316344 | 0.00013 | 0.000919 |
| RPH3A | -2.04757 | 0.000687 | 0.003727 |
| RPL39 | 2.391613 | 2.57E-05 | 0.000238 |
| RPLP0 | 2.331189 | 2.46E-08 | 6.67E-07 |
| RPLP0P2 | -3.74283 | 8.82E-27 | 1.57E-22 |
| RPRML | -2.96415 | 6.02E-08 | 1.41E-06 |
| RPS27 | 4.441257 | 7.75E-05 | 0.000601 |
| RRM2 | 6.367587 | 0.000016 | 0.00016 |
| RS1 | -2.83013 | 1.76E-08 | 4.99E-07 |
| RSPO2 | -2.41977 | 3.27E-05 | 0.000291 |
| RSPO3 | -2.23577 | 0.000468 | 0.002711 |
| RTBDN | -3.19521 | 1.94E-10 | 1.17E-08 |
| RTL1 | -3.65858 | 1.48E-16 | 3.74E-13 |
| RTN4R | -2.38891 | 2.46E-08 | 6.67E-07 |
| RTN4RL1 | -3.10577 | 1.07E-08 | 3.27E-07 |
| RTN4RL2 | -2.44588 | 2E-09 | 7.92E-08 |
| RXFP1 | -3.21525 | 8.85E-07 | 1.35E-05 |
| RXFP3 | -2.57806 | 0.000138 | 0.00097 |
| RYR2 | -3.49396 | 1.22E-08 | 3.65E-07 |
| S100A10 | 2.424663 | 0.006092 | 0.022195 |
| S100A2 | 2.594774 | 0.003709 | 0.014852 |
| S1PR3 | 2.280909 | 0.003075 | 0.012778 |
| S1PR5 | -2.6337 | 9.74E-08 | 2.1E-06 |
| SAMD12 | -2.77496 | 1.96E-11 | 1.9E-09 |
| SCN2A | -2.70508 | 3.85E-10 | 2.02E-08 |
| SCN2B | -2.55448 | 9.12E-08 | 0.000002 |
| SCN3B | -2.56741 | 4.19E-07 | 7.07E-06 |
| SCN5A | -2.97069 | 1.82E-09 | 7.33E-08 |
| SCN8A | -2.49247 | 1.6E-07 | 3.2E-06 |
| SCUBE2 | 2.725322 | 0.001558 | 0.007321 |
| SDR16C5 | -2.96579 | 1.55E-05 | 0.000155 |
| SEC14L5 | -2.47286 | 9.32E-06 | 0.0001 |
| SELE | -3.09259 | 9.09E-05 | 0.000686 |
| SELL | 4.416279 | 0.001735 | 0.008018 |
| SEMA4A | -2.45804 | 1.03E-11 | 1.17E-09 |
| SEMA4D | -2.09259 | 3.49E-09 | 1.27E-07 |
| SERINC2 | 2.682531 | 0.006413 | 0.02314 |
| SERPIND1 | -3.55962 | 5.91E-08 | 1.39E-06 |
| SERPINH1 | 2.530089 | 0.004105 | 0.016123 |
| SERPINI1 | -2.67607 | 1.07E-08 | 3.27E-07 |
| SFRP4 | 3.038837 | 0.007471 | 0.026094 |
| SGIP1 | -2.10255 | 6.14E-10 | 2.95E-08 |
| SGK2 | -2.10248 | 8.25E-06 | 9.05E-05 |
| SGOL1 | 4.870075 | 0.000201 | 0.001325 |
| SH2D1B | -2.69879 | 3.55E-08 | 9.04E-07 |
| SH2D5 | -3.30404 | 5.49E-10 | 2.68E-08 |
| SH3GL3 | -2.5676 | 1.2E-07 | 2.5E-06 |
| SH3RF2 | -2.84883 | 6.89E-07 | 1.09E-05 |
| SH3TC2 | -2.08065 | 1.66E-05 | 0.000164 |
| SHANK1 | -3.03042 | 1.11E-09 | 4.96E-08 |
| SHCBP1 | 2.203342 | 0.007987 | 0.027538 |
| SHD | 3.979277 | 0.000277 | 0.001746 |
| SHOX2 | 7.118911 | 0.007913 | 0.027342 |
| SIAH3 | -2.61633 | 1.17E-05 | 0.000122 |
| SIGLEC7 | 3.36741 | 0.000555 | 0.003122 |
| SIX1 | 4.386819 | 3.26E-05 | 0.000291 |
| SKA1 | 4.03641 | 0.00055 | 0.003099 |
| SKA3 | 3.484959 | 0.000699 | 0.003784 |
| SLC12A5 | -3.31231 | 4.05E-08 | 1.01E-06 |
| SLC13A5 | -2.04289 | 0.005595 | 0.020728 |
| SLC14A2 | 3.985863 | 0.014417 | 0.043757 |
| SLC16A4 | 2.487761 | 0.000513 | 0.002927 |
| SLC16A8 | -2.04478 | 5.37E-06 | 6.29E-05 |
| SLC17A6 | -3.17682 | 9.29E-07 | 0.000014 |
| SLC17A7 | -3.51007 | 1.88E-08 | 5.31E-07 |
| SLC22A10 | -3.46173 | 1.11E-08 | 3.4E-07 |
| SLC22A15 | -2.39955 | 4.75E-08 | 1.16E-06 |
| SLC22A2 | -2.75167 | 0.000362 | 0.002182 |
| SLC22A25 | -2.06474 | 0.007993 | 0.027547 |
| SLC22A8 | -4.40168 | 1.54E-08 | 4.42E-07 |
| SLC22A9 | -3.74673 | 1.71E-07 | 3.36E-06 |
| SLC24A2 | -2.14134 | 0.000155 | 0.001063 |
| SLC25A2 | 2.70016 | 0.014827 | 0.044718 |
| SLC25A22 | -2.15771 | 1.91E-13 | 4.78E-11 |
| SLC26A4 | -2.37042 | 2.08E-07 | 0.000004 |
| SLC26A8 | -3.33764 | 9.42E-12 | 1.09E-09 |
| SLC27A2 | -3.1275 | 3.1E-07 | 5.53E-06 |
| SLC28A1 | 3.13956 | 0.010816 | 0.034953 |
| SLC2A14 | -2.52998 | 3.4E-10 | 1.81E-08 |
| SLC2A3 | -2.1477 | 9.96E-08 | 2.14E-06 |
| SLC30A3 | -3.65257 | 1.99E-09 | 7.89E-08 |
| SLC30A8 | -2.14933 | 0.001851 | 0.008422 |
| SLC32A1 | -3.16988 | 2.66E-07 | 4.89E-06 |
| SLC35F3 | -2.85652 | 5.22E-08 | 1.26E-06 |
| SLC37A2 | 2.320514 | 0.000596 | 0.003326 |
| SLC38A8 | -2.65545 | 5.96E-05 | 0.000482 |
| SLC40A1 | 2.097162 | 0.000221 | 0.00144 |
| SLC46A2 | -2.21505 | 3E-08 | 7.93E-07 |
| SLC4A10 | -2.93213 | 1.49E-08 | 4.3E-07 |
| SLC5A11 | -2.53595 | 3.98E-05 | 0.000344 |
| SLC5A5 | -3.42002 | 1.3E-09 | 5.71E-08 |
| SLC5A7 | -2.3156 | 0.011798 | 0.037503 |
| SLC6A13 | -2.10424 | 9.54E-06 | 0.000102 |
| SLC6A15 | -2.9247 | 3.36E-07 | 5.93E-06 |
| SLC6A17 | -3.42053 | 1.3E-09 | 5.69E-08 |
| SLC6A20 | -2.74521 | 5.83E-06 | 6.73E-05 |
| SLC6A5 | -3.4036 | 0.000911 | 0.004724 |
| SLC6A7 | -3.53792 | 2.97E-07 | 5.34E-06 |
| SLC7A4 | -3.19949 | 2.15E-09 | 8.44E-08 |
| SLC8A2 | -3.26974 | 1.95E-10 | 1.17E-08 |
| SLCO4C1 | -2.72178 | 3.6E-07 | 6.25E-06 |
| SLCO5A1 | 2.759631 | 0.002116 | 0.009415 |
| SLFN13 | 2.074227 | 0.012641 | 0.039549 |
| SLIT3 | -2.90279 | 1.36E-10 | 8.63E-09 |
| SLITRK4 | -2.43671 | 2.22E-05 | 0.00021 |
| SMCP | 7.555085 | 0.007949 | 0.027434 |
| SMO | 2.421199 | 2.45E-06 | 3.23E-05 |
| SMOC1 | 2.98224 | 0.007296 | 0.025585 |
| SMPX | -3.37524 | 5.64E-06 | 6.54E-05 |
| SMYD1 | -2.53616 | 3.26E-05 | 0.000291 |
| SNAP25 | -2.9237 | 1.66E-07 | 3.3E-06 |
| SNAP91 | -2.24444 | 2.16E-05 | 0.000206 |
| SNCA | -3.08177 | 1.16E-12 | 2.15E-10 |
| SNCAIP | 2.936557 | 6.78E-07 | 1.08E-05 |
| SNCB | -2.81127 | 7.01E-07 | 1.11E-05 |
| SNCG | -2.6785 | 3.89E-07 | 6.62E-06 |
| SNORD115-13 | -3.0287 | 9.43E-06 | 0.000101 |
| SNORD115-26 | -2.56647 | 5.93E-05 | 0.00048 |
| SNPH | -2.02053 | 2.18E-09 | 8.47E-08 |
| SNURF | -2.06658 | 5.58E-11 | 4.23E-09 |
| SNX20 | 2.426946 | 0.000106 | 0.000779 |
| SOCS1 | 2.23078 | 0.015614 | 0.046484 |
| SOHLH1 | -2.50106 | 0.000913 | 0.004731 |
| SORBS2 | -2.32221 | 8.28E-11 | 5.74E-09 |
| SOSTDC1 | -2.89743 | 6.34E-08 | 1.47E-06 |
| SOX11 | 3.793245 | 1.8E-06 | 2.48E-05 |
| SOX2 | 2.433633 | 1.34E-09 | 5.82E-08 |
| SOX3 | 2.98146 | 0.001379 | 0.00662 |
| SOX4 | 3.722689 | 4.79E-07 | 7.92E-06 |
| SOX6 | 2.383258 | 0.000121 | 0.000871 |
| SP9 | -2.09656 | 0.002891 | 0.01215 |
| SPARC | 3.480212 | 3.41E-08 | 8.77E-07 |
| SPATA12 | 2.488099 | 0.001958 | 0.008827 |
| SPC24 | 3.327029 | 0.011652 | 0.037136 |
| SPC25 | 4.035055 | 3.14E-05 | 0.000282 |
| SPIN2A | -2.71377 | 3.43E-05 | 0.000303 |
| SPINK8 | 4.613312 | 0.002686 | 0.011446 |
| SPINT2 | -2.29238 | 2.8E-12 | 4.35E-10 |
| SPOCD1 | 3.940846 | 0.014877 | 0.044835 |
| SPOCK3 | -2.19726 | 1.77E-05 | 0.000174 |
| SPRR2G | -2.3237 | 0.000929 | 0.004794 |
| SPSB4 | 2.790258 | 9.44E-06 | 0.000101 |
| SPTB | -2.93082 | 9.2E-11 | 6.28E-09 |
| SPTBN2 | -2.04074 | 2.15E-06 | 2.87E-05 |
| SPTBN4 | -2.10886 | 8.62E-10 | 3.97E-08 |
| SRCIN1 | -2.41325 | 2.25E-08 | 6.21E-07 |
| SRPX | 2.817247 | 0.000623 | 0.003446 |
| SRPX2 | 4.066517 | 0.010452 | 0.034049 |
| SRRM4 | -2.48716 | 3.35E-05 | 0.000297 |
| SST | -3.72372 | 1.73E-12 | 3.06E-10 |
| SSTR3 | -3.4223 | 1.41E-07 | 2.85E-06 |
| SSTR4 | -3.46846 | 2.36E-07 | 4.39E-06 |
| ST14 | 3.161984 | 0.001305 | 0.006327 |
| ST18 | -2.05827 | 0.000577 | 0.003232 |
| ST6GALNAC5 | -2.88633 | 3.78E-07 | 6.48E-06 |
| ST8SIA3 | -2.22658 | 0.000138 | 0.000969 |
| STAC2 | -2.1695 | 0.000198 | 0.001312 |
| STAT4 | -2.58967 | 4.29E-05 | 0.000366 |
| STC2 | 2.926938 | 0.001353 | 0.00652 |
| STEAP3 | 2.67019 | 0.014068 | 0.042859 |
| STK17B | 2.573347 | 7.92E-07 | 1.23E-05 |
| STK32A | 2.777737 | 0.000104 | 0.000771 |
| STK32C | -2.05471 | 1.51E-10 | 9.38E-09 |
| STK33 | 2.280262 | 3.38E-07 | 5.94E-06 |
| STMN2 | -2.31459 | 0.000183 | 0.00123 |
| STON1 | 3.946452 | 4.28E-08 | 1.06E-06 |
| STRC | -2.01026 | 6.36E-07 | 1.02E-05 |
| STX1A | -3.07159 | 1.32E-11 | 1.41E-09 |
| STX1B | -2.51803 | 1.32E-09 | 5.78E-08 |
| STXBP1 | -2.63987 | 3.7E-12 | 5.51E-10 |
| STXBP5 | -2.27974 | 3.39E-10 | 1.81E-08 |
| STXBP5L | -2.29833 | 1.02E-05 | 0.000109 |
| STXBP6 | -2.21013 | 5.31E-07 | 8.67E-06 |
| STYK1 | -3.34749 | 2.2E-07 | 4.15E-06 |
| SULF2 | 2.087971 | 0.000215 | 0.001405 |
| SULT1C4 | 2.250106 | 5.11E-05 | 0.000424 |
| SULT4A1 | -3.26224 | 7.17E-08 | 1.62E-06 |
| SV2B | -3.5592 | 2.6E-09 | 9.88E-08 |
| SV2C | -3.91566 | 3.2E-11 | 2.71E-09 |
| SVOP | -2.66684 | 3.35E-05 | 0.000297 |
| SYCE2 | 2.605413 | 0.00055 | 0.003099 |
| SYN1 | -3.00424 | 3.72E-10 | 1.96E-08 |
| SYN2 | -3.64239 | 4.68E-11 | 3.73E-09 |
| SYNGR1 | -2.3691 | 1.84E-11 | 1.82E-09 |
| SYNGR3 | -3.11309 | 2.75E-10 | 1.54E-08 |
| SYNGR4 | -2.42097 | 8.03E-05 | 0.000618 |
| SYNJ2 | -2.5542 | 2.17E-09 | 8.47E-08 |
| SYNPO | -2.20184 | 3.46E-06 | 4.32E-05 |
| SYNPR | -3.29674 | 4.25E-08 | 1.05E-06 |
| SYP | -2.78406 | 5.05E-12 | 6.73E-10 |
| SYT1 | -3.2949 | 9.27E-09 | 2.9E-07 |
| SYT10 | -2.46071 | 0.003869 | 0.015389 |
| SYT12 | -2.22174 | 1.19E-08 | 3.57E-07 |
| SYT13 | -3.00321 | 3.72E-07 | 6.4E-06 |
| SYT16 | -2.11107 | 0.0001 | 0.000744 |
| SYT4 | -2.60739 | 2.48E-05 | 0.000231 |
| SYT5 | -3.14737 | 2.41E-10 | 1.38E-08 |
| SYT7 | -3.1862 | 4.82E-11 | 3.78E-09 |
| SYTL1 | -2.16002 | 7.77E-06 | 8.59E-05 |
| SYTL4 | 3.260474 | 5.79E-06 | 0.000067 |
| SYTL5 | -2.25362 | 2.92E-05 | 0.000265 |
| TAC3 | -2.76738 | 3.68E-05 | 0.000323 |
| TAC4 | 3.761299 | 0.012023 | 0.03804 |
| TACC3 | 3.047211 | 0.002807 | 0.011858 |
| TACR2 | -2.41199 | 2.52E-12 | 4.14E-10 |
| TACR3 | -2.2102 | 0.001476 | 0.00699 |
| TAGLN3 | -2.50468 | 4.94E-09 | 1.73E-07 |
| TBC1D26 | -2.24381 | 3.23E-06 | 4.08E-05 |
| TBR1 | -3.14609 | 1.09E-06 | 0.000016 |
| TBX15 | 3.006296 | 0.000201 | 0.001329 |
| TBX2 | 2.033691 | 0.000284 | 0.001783 |
| TBX4 | 3.440963 | 0.013283 | 0.040944 |
| TBX5 | 7.984432 | 0.002218 | 0.009775 |
| TCEAL6 | -2.35807 | 3.54E-08 | 9.03E-07 |
| TCERG1L | -2.81655 | 1.49E-05 | 0.00015 |
| TCF12 | 2.732767 | 6.12E-07 | 9.88E-06 |
| TCF19 | 2.401546 | 0.000129 | 0.000914 |
| TCF7L1 | 2.442877 | 1.69E-07 | 3.34E-06 |
| TDRD5 | -2.19115 | 0.000117 | 0.000847 |
| TEAD2 | 2.176546 | 0.006298 | 0.022812 |
| TEKT1 | 4.510405 | 0.014024 | 0.042762 |
| TEX19 | 3.42556 | 0.010455 | 0.034049 |
| TEX9 | 2.325884 | 8.15E-05 | 0.000626 |
| TFAP2A | 4.29065 | 0.000211 | 0.001382 |
| TFPI | 3.539914 | 0.000891 | 0.004634 |
| TGFB1I1 | 2.807302 | 9.15E-06 | 9.89E-05 |
| TGFBI | 3.635235 | 0.004157 | 0.016291 |
| TGM5 | 4.8583 | 0.006696 | 0.023923 |
| TH | -2.06065 | 0.006313 | 0.022857 |
| THEM5 | -2.94599 | 6.9E-10 | 3.25E-08 |
| THEMIS | -2.89133 | 3.26E-08 | 8.43E-07 |
| THRB | -2.18477 | 1.62E-09 | 6.74E-08 |
| TIMP4 | 4.497866 | 6.97E-07 | 0.000011 |
| TJP3 | -2.55553 | 0.000225 | 0.001456 |
| TKTL1 | 3.441236 | 0.009736 | 0.032143 |
| TLE6 | 2.757205 | 0.001051 | 0.005303 |
| TLX1 | 8.379047 | 0.000373 | 0.00224 |
| TLX1NB | 8.30316 | 0.004661 | 0.017894 |
| TM7SF4 | -2.00586 | 0.005139 | 0.019374 |
| TMC5 | -2.35682 | 8.33E-07 | 1.28E-05 |
| TMEM100 | 3.886832 | 6.02E-05 | 0.000487 |
| TMEM125 | -2.14084 | 0.000718 | 0.003862 |
| TMEM130 | -3.2568 | 6.2E-09 | 2.1E-07 |
| TMEM132D | -2.94664 | 1.12E-05 | 0.000118 |
| TMEM150B | 3.587133 | 0.008472 | 0.028823 |
| TMEM151A | -2.4833 | 7.86E-08 | 1.77E-06 |
| TMEM155 | -3.11447 | 1.67E-07 | 3.31E-06 |
| TMEM171 | -2.36165 | 0.000363 | 0.002189 |
| TMEM189-UBE2V1 | -2.43436 | 0.003452 | 0.014022 |
| TMEM191A | -2.67456 | 3.11E-07 | 5.55E-06 |
| TMEM195 | 3.271828 | 0.001967 | 0.00886 |
| TMEM215 | -3.0253 | 5.38E-06 | 0.000063 |
| TMEM233 | -3.29162 | 1.47E-09 | 6.22E-08 |
| TMEM45A | 2.812763 | 0.00018 | 0.001209 |
| TMEM52 | -2.40237 | 8.81E-06 | 9.57E-05 |
| TMEM88B | -3.19008 | 4.58E-07 | 7.59E-06 |
| TMOD4 | 3.702403 | 8.89E-05 | 0.000673 |
| TMSB15A | 4.946638 | 0.000315 | 0.001943 |
| TNC | 3.034536 | 0.000666 | 0.003641 |
| TNFRSF10C | 2.470834 | 0.001374 | 0.006599 |
| TNFRSF19 | 3.165167 | 2.93E-05 | 0.000266 |
| TNFSF13B | 2.900044 | 0.004672 | 0.017927 |
| TNIP3 | -3.00401 | 4.16E-06 | 5.06E-05 |
| TNNT2 | -2.27701 | 0.010464 | 0.034071 |
| TOP2A | 6.732825 | 0.000004 | 0.000049 |
| TP53 | 2.19303 | 4.45E-07 | 7.42E-06 |
| TP53I11 | -2.02635 | 5.25E-12 | 6.9E-10 |
| TP53I3 | 2.245296 | 4.63E-05 | 0.000389 |
| TP53TG5 | -3.35064 | 1.59E-07 | 3.18E-06 |
| TPH2 | -3.25574 | 3.77E-06 | 4.68E-05 |
| TPPP | -2.49587 | 6.06E-08 | 1.42E-06 |
| TPX2 | 2.994153 | 0.001348 | 0.0065 |
| TRHDE | -3.25231 | 4.38E-07 | 7.34E-06 |
| TRIM5 | 2.400701 | 2.81E-05 | 0.000257 |
| TRIM54 | -2.67651 | 5.48E-06 | 0.000064 |
| TRIM59 | -2.31015 | 2.98E-07 | 5.36E-06 |
| TRIM72 | -3.29741 | 1.05E-09 | 4.72E-08 |
| TRIML2 | -3.48611 | 2.44E-09 | 9.38E-08 |
| TROAP | 5.899427 | 4.02E-05 | 0.000347 |
| TRPC5 | -2.86256 | 2.27E-06 | 3.02E-05 |
| TRPM6 | -2.15437 | 7.05E-07 | 1.11E-05 |
| TRPV4 | 2.914356 | 0.007939 | 0.027409 |
| TRPV5 | -2.00344 | 0.002013 | 0.009029 |
| TRPV6 | -2.5724 | 8.98E-07 | 1.36E-05 |
| TSHR | 5.644307 | 0.00112 | 0.005584 |
| TSPAN11 | 2.731717 | 3.09E-06 | 3.93E-05 |
| TSPAN12 | 2.788236 | 7.02E-06 | 7.86E-05 |
| TSPAN19 | -2.07888 | 0.005799 | 0.021327 |
| TSPAN6 | 2.623131 | 2.25E-10 | 1.31E-08 |
| TSPYL5 | -2.24693 | 4.67E-08 | 1.14E-06 |
| TTC22 | -2.53553 | 0.000182 | 0.001225 |
| TTC9B | -2.38803 | 1.24E-06 | 0.000018 |
| TTK | 4.667622 | 0.000399 | 0.00237 |
| TUBA1C | 2.750397 | 0.010818 | 0.034953 |
| TUBA4A | -2.49401 | 5.29E-08 | 1.27E-06 |
| TUBA8 | -2.5841 | 7.03E-09 | 2.3E-07 |
| TUBB4 | -2.13422 | 1.37E-06 | 1.95E-05 |
| TUBBP5 | -3.24925 | 2.17E-10 | 1.28E-08 |
| TXNIP | 2.051268 | 3.23E-07 | 5.73E-06 |
| TYRP1 | -2.68042 | 0.000498 | 0.002855 |
| UBD | 4.676117 | 0.000687 | 0.003729 |
| UBE2C | 6.752625 | 0.000011 | 0.000116 |
| UBE2QL1 | -2.27144 | 4.45E-07 | 7.42E-06 |
| UCHL1 | -2.10479 | 4.59E-09 | 1.64E-07 |
| UHRF1 | 3.956049 | 3.35E-09 | 1.23E-07 |
| UNC13A | -2.17399 | 2.65E-06 | 3.44E-05 |
| UNC13C | -2.42713 | 0.000579 | 0.003241 |
| UNC5A | -2.10684 | 1.61E-05 | 0.000161 |
| UNC5C | -2.23309 | 1.36E-05 | 0.000138 |
| UNC5D | -2.48245 | 1.63E-05 | 0.000162 |
| UPK1B | -2.2027 | 0.014878 | 0.044835 |
| UST | 2.564715 | 4.1E-06 | 0.00005 |
| UTS2R | -2.59882 | 6.12E-05 | 0.000493 |
| VANGL2 | 2.481698 | 3.95E-10 | 2.06E-08 |
| VAX1 | -2.01246 | 0.002818 | 0.011893 |
| VAX2 | 3.843747 | 2.48E-06 | 3.27E-05 |
| VCAM1 | 2.691554 | 0.00445 | 0.017249 |
| VCAN | 2.55807 | 4.07E-06 | 4.98E-05 |
| VEPH1 | 6.311099 | 0.000222 | 0.00144 |
| VIM | 2.491688 | 0.004866 | 0.01849 |
| VIP | -2.94252 | 2.09E-06 | 0.000028 |
| VIPR1 | -2.8326 | 9.31E-11 | 6.28E-09 |
| VIPR2 | 3.21291 | 0.011409 | 0.03654 |
| VNN1 | 3.147094 | 0.001565 | 0.007349 |
| VSIG4 | 2.573097 | 0.003595 | 0.014493 |
| VSNL1 | -3.15764 | 8.95E-07 | 1.36E-05 |
| VSTM2B | -2.31606 | 1.45E-06 | 2.05E-05 |
| VSTM2L | -2.55538 | 4.82E-07 | 7.95E-06 |
| VTCN1 | 2.844269 | 0.001081 | 0.005433 |
| VWA5B2 | -2.27468 | 1.25E-05 | 0.000129 |
| WBSCR17 | -3.09454 | 1.29E-08 | 3.83E-07 |
| WDR38 | 5.488745 | 0.014962 | 0.045004 |
| WDR49 | 2.396945 | 0.003309 | 0.013561 |
| WDR63 | 3.950869 | 0.016755 | 0.049092 |
| WDR76 | 2.484404 | 4.76E-05 | 0.000398 |
| WEE1 | 3.180983 | 0.000109 | 0.000798 |
| WIF1 | -2.90192 | 0.000306 | 0.001898 |
| WNT1 | -3.33201 | 3.16E-10 | 1.73E-08 |
| WNT10B | -3.45561 | 3.89E-10 | 2.04E-08 |
| WNT8A | -3.24161 | 8.12E-11 | 5.65E-09 |
| WSCD2 | -2.79784 | 3.81E-06 | 4.72E-05 |
| XIRP2 | 3.472732 | 0.003898 | 0.015474 |
| XK | -2.62852 | 3.65E-07 | 6.31E-06 |
| XKR5 | 3.638745 | 0.00111 | 0.005545 |
| XPNPEP2 | 2.919184 | 0.003962 | 0.015689 |
| YWHAG | -2.10081 | 1.95E-16 | 3.85E-13 |
| YWHAH | -2.4728 | 6.89E-16 | 8.4E-13 |
| ZBTB20 | 2.348351 | 0.00126 | 0.00615 |
| ZCCHC12 | -3.32794 | 1.6E-11 | 1.64E-09 |
| ZCCHC16 | -2.04789 | 0.002263 | 0.009943 |
| ZDHHC23 | -2.0002 | 0.00025 | 0.001596 |
| ZDHHC8P1 | -2.25931 | 0.000142 | 0.000988 |
| ZFP57 | -2.18458 | 0.000226 | 0.001464 |
| ZIC1 | 2.180022 | 0.000955 | 0.004906 |
| ZIM2 | -2.12657 | 0.00012 | 0.000862 |
| ZMAT4 | -2.17328 | 0.00016 | 0.001092 |
| ZNF204P | -2.33066 | 4.12E-08 | 1.02E-06 |
| ZNF215 | -2.1525 | 2.65E-06 | 3.44E-05 |
| ZNF300 | 2.302058 | 4.26E-06 | 5.17E-05 |
| ZNF365 | -2.3113 | 8.41E-10 | 3.89E-08 |
| ZNF385B | -2.27494 | 7.54E-05 | 0.000586 |
| ZNF536 | -2.67528 | 2.93E-08 | 7.79E-07 |
| ZNF560 | 5.536747 | 0.005126 | 0.019344 |
| ZNF702P | -2.14587 | 1.67E-07 | 3.31E-06 |
| ZNF831 | -3.30716 | 6.5E-10 | 3.09E-08 |
| ZNF90 | 2.620272 | 0.000939 | 0.004836 |
| ZNRF4 | -2.0191 | 0.001089 | 0.005464 |

**Table S3.** Gene ontology enrichment analysis of upregulated and downregulated genes.

| Category | Term | Count | % | P-Value | FDR |
| --- | --- | --- | --- | --- | --- |
| Upregulated |  |  |  |  |  |
| GOTERM_BP_DIRECT | cell division | 48 | 8 | 7.10E-17 | 2.00E-13 |
| GOTERM_BP_DIRECT | mitotic nuclear division | 38 | 6.3 | 6.50E-15 | 1.10E-11 |
| GOTERM_BP_DIRECT | sister chromatid cohesion | 25 | 4.2 | 1.70E-14 | 3.00E-11 |
| GOTERM_BP_DIRECT | embryonic skeletal system morphogenesis | 16 | 2.7 | 4.50E-13 | 7.90E-10 |
| GOTERM_BP_DIRECT | anterior/posterior pattern specification | 17 | 2.8 | 4.60E-09 | 8.10E-06 |
| GOTERM_CC_DIRECT | condensed chromosome kinetochore | 17 | 2.8 | 9.30E-09 | 1.30E-05 |
| GOTERM_CC_DIRECT | extracellular matrix | 30 | 5 | 5.10E-08 | 7.00E-05 |
| GOTERM_CC_DIRECT | basement membrane | 15 | 2.5 | 1.30E-07 | 1.80E-04 |
| GOTERM_CC_DIRECT | chromosome, centromeric region | 13 | 2.2 | 1.40E-07 | 1.90E-04 |
| GOTERM_CC_DIRECT | proteinaceous extracellular matrix | 26 | 4.3 | 1.10E-06 | 1.50E-03 |
| GOTERM_MF_DIRECT | sequence-specific DNA binding | 50 | 8.3 | 5.90E-12 | 8.80E-09 |
| GOTERM_MF_DIRECT | transcription factor activity, sequence-specific DNA binding | 64 | 10.6 | 2.40E-08 | 3.60E-05 |
| GOTERM_MF_DIRECT | transcriptional activator activity, RNA polymerase II core promoter proximal region sequence-specific binding | 24 | 4 | 1.70E-06 | 2.50E-03 |
| GOTERM_MF_DIRECT | collagen binding | 11 | 1.8 | 1.60E-05 | 2.40E-02 |
| GOTERM_MF_DIRECT | chromatin binding | 29 | 4.8 | 4.80E-05 | 7.20E-02 |
| Downregulated |  |  |  |  |  |
| GOTERM_BP_DIRECT | chemical synaptic transmission | 86 | 8.4 | 1.30E-47 | 2.30E-44 |
| GOTERM_BP_DIRECT | neurotransmitter secretion | 24 | 2.4 | 1.70E-16 | 2.00E-13 |
| GOTERM_BP_DIRECT | G-protein coupled receptor signaling pathway, coupled to cyclic nucleotide second messenger | 22 | 2.2 | 2.80E-15 | 4.90E-12 |
| GOTERM_BP_DIRECT | regulation of ion transmembrane transport | 31 | 3 | 6.80E-14 | 1.20E-10 |
| GOTERM_BP_DIRECT | potassium ion transport | 26 | 2.6 | 4.20E-13 | 7.40E-10 |
| GOTERM_CC_DIRECT | plasma membrane | 396 | 38.9 | 1.80E-40 | 2.50E-37 |
| GOTERM_CC_DIRECT | cell junction | 102 | 10 | 3.90E-36 | 5.50E-33 |
| GOTERM_CC_DIRECT | integral component of plasma membrane | 169 | 16.6 | 8.10E-25 | 1.20E-21 |
| GOTERM_CC_DIRECT | postsynaptic membrane | 52 | 5.1 | 1.20E-20 | 1.70E-17 |
| GOTERM_CC_DIRECT | voltage-gated potassium channel complex | 33 | 3.2 | 3.60E-19 | 5.10E-16 |
| GOTERM_MF_DIRECT | calcium-dependent phospholipid binding | 21 | 2.1 | 4.30E-12 | 6.80E-09 |
| GOTERM_MF_DIRECT | calmodulin binding | 36 | 3.5 | 3.80E-11 | 5.90E-08 |
| GOTERM_MF_DIRECT | voltage-gated potassium channel activity | 20 | 2 | 4.20E-11 | 6.50E-08 |
| GOTERM_MF_DIRECT | calcium ion binding | 79 | 7.8 | 3.10E-10 | 4.90E-07 |
| GOTERM_MF_DIRECT | clathrin binding | 18 | 1.8 | 4.60E-10 | 7.20E-07 |

**Table S4.** KEGG pathway enrichment analysis of upregulated and downregulated genes.

| GOID | GOTerm | p value | Nr. Genes | Associated Genes Found |
| --- | --- | --- | --- | --- |
| Upregulated |  |  |  |  |
| KEGG:04110 | Cell cycle | 1.34893E-12 | 23 | BUB1, BUB1B, CCNA2, CCNB2, CDC25A, CDC25C, CDC45, CDK1, CDK2, CDK4, CDKN2C, CHEK2, E2F2, ESPL1, GADD45G, MYC, ORC1, PKMYT1, PLK1, PTTG1, TP53, TTK, WEE1 |
| KEGG:05202 | Transcriptional misregulation in cancer | 0.000457086 | 15 | CDKN2C, ETV1, ETV4, EYA1, GADD45G, HOXA10, HOXA11, IGFBP3, IL1R2, MMP9, MYC, NUPR1, SIX1, TLX1, TP53 |
| KEGG:05219 | Bladder cancer | 0.000190111 | 7 | CDK4, E2F2, EGFR, MMP2, MMP9, MYC, TP53 |
| KEGG:05222 | Small cell lung cancer | 1.96659E-05 | 12 | CDK2, CDK4, CKS2, COL4A1, COL4A2, COL4A6, E2F2, FN1, GADD45G, LAMB4, MYC, TP53 |
| KEGG:04115 | p53 signaling pathway | 6.67304E-07 | 12 | CCNB2, CDK1, CDK2, CDK4, CHEK2, GADD45G, GTSE1, IGFBP3, RRM2, STEAP3, TP53, TP53I3 |
| KEGG:04218 | Cellular senescence | 2.26004E-05 | 16 | CCNA2, CCNB2, CDC25A, CDK1, CDK2, CDK4, CHEK2, E2F2, EIF4EBP1, FOXM1, GADD45G, IGFBP3, MYBL2, MYC, TP53, TRPV4 |
| KEGG:04114 | Oocyte meiosis | 8.26946E-05 | 13 | AR, AURKA, BUB1, CCNB2, CDC25C, CDK1, CDK2, ESPL1, FBXO43, PKMYT1, PLK1, PTTG1, SGO1 |
| KEGG:04914 | Progesterone-mediated oocyte maturation | 0.000739893 | 10 | AURKA, BUB1, CCNA2, CCNB2, CDC25A, CDC25C, CDK1, CDK2, PKMYT1, PLK1 |
| Downregulated |  |  |  |  |
| KEGG:04020 | Calcium signaling pathway | 3.43532E-22 | 48 | ADCY1, ADRA1B, ADRB1, ADRB3, ATP2B1, ATP2B2, ATP2B3, CACNA1B, CACNA1C, CACNA1E, CACNA1F, CACNA1G, CACNA1I, CACNA1S, CALM3, CAMK2A, CAMK2B, CAMK4, CCKBR, CHRM1, CHRM2, CHRM3, DRD1, DRD5, GRIN1, GRIN2A, GRM1, GRM5, HRH2, HTR2A, HTR4, HTR5A, HTR6, HTR7, ITPKA, ITPR1, NTSR1, P2RX2, PDE1A, PDE1B, PRKCB, PRKCG, PTGER3, PTK2B, RYR2, SLC8A2, TACR2, TACR3 |
| KEGG:04726 | Serotonergic synapse | 8.34704E-12 | 27 | CACNA1B, CACNA1C, CACNA1F, CACNA1S, CYP2C18, CYP4X1, GABRB2, GNG13, GNG3, HTR1A, HTR1B, HTR1E, HTR2A, HTR3A, HTR3B, HTR4, HTR5A, HTR6, HTR7, ITPR1, KCNJ3, KCNJ6, KCNJ9, PLA2G4D, PRKCB, PRKCG, TPH2 |
| KEGG:04742 | Taste transduction | 5.96066E-10 | 21 | ASIC2, CACNA1C, CALHM1, CHRM3, GABRA1, GABRA2, GABRA4, GABRA5, GNG13, GRM1, GRM4, HTR1A, HTR1B, HTR1E, HTR3A, HTR3B, P2RX2, PDE1A, PDE1B, PKD2L1, SCN2A |
| KEGG:04080 | Neuroactive ligand-receptor interaction | 1.67539E-34 | 74 | ADRA1B, ADRA2C, ADRB1, ADRB3, CCKBR, CHRM1, CHRM2, CHRM3, CHRNA2, CHRNB3, CRHR1, CRHR2, DRD1, DRD5, GABRA1, GABRA2, GABRA4, GABRA5, GABRB2, GABRD, GABRG2, GABRG3, GIPR, GLP2R, GLRA2, GPR83, GRIN1, GRIN2A, GRIN2B, GRM1, GRM2, GRM3, GRM4, GRM5, GRM7, HCRTR2, HRH2, HRH3, HTR1A, HTR1B, HTR1E, HTR2A, HTR4, HTR5A, HTR6, HTR7, LPAR3, MAS1, MC4R, MCHR2, MTNR1A, NPBWR1, NPFFR1, NPFFR2, NPY1R, NPY5R, NTSR1, OPRD1, OPRK1, OPRM1, P2RX2, PRSS3, PTGER3, PTH2R, RXFP1, RXFP3, S1PR5, SSTR3, SSTR4, TACR2, TACR3, THRB, UTS2R, VIPR1 |
| KEGG:04721 | Synaptic vesicle cycle | 1.21709E-09 | 18 | CACNA1B, CPLX1, CPLX2, CPLX3, DNM1, NSF, RAB3A, RIMS1, SLC17A6, SLC17A7, SLC32A1, SNAP25, STX1A, STX1B, STXBP1, SYT1, UNC13A, UNC13C |
| KEGG:04723 | Retrograde endocannabinoid signaling | 5.45389E-09 | 27 | ADCY1, CACNA1B, CACNA1C, CACNA1F, CACNA1S, GABRA1, GABRA2, GABRA4, GABRA5, GABRB2, GABRD, GABRG2, GABRG3, GNG13, GNG3, GRM1, GRM5, ITPR1, KCNJ3, KCNJ6, KCNJ9, PRKCB, PRKCG, RIMS1, SLC17A6, SLC17A7, SLC32A1 |
| KEGG:04725 | Cholinergic synapse | 3.69284E-08 | 22 | ADCY1, CACNA1B, CACNA1C, CACNA1F, CACNA1S, CAMK2A, CAMK2B, CAMK4, CHRM1, CHRM2, CHRM3, GNG13, GNG3, ITPR1, KCNJ12, KCNJ3, KCNJ4, KCNJ6, KCNQ3, PRKCB, PRKCG, SLC5A7 |
| KEGG:04727 | GABAergic synapse | 9.35162E-14 | 26 | ADCY1, CACNA1B, CACNA1C, CACNA1F, CACNA1S, GABARAPL1, GABRA1, GABRA2, GABRA4, GABRA5, GABRB2, GABRD, GABRG2, GABRG3, GAD2, GLS, GLS2, GNG13, GNG3, KCNJ6, NSF, PRKCB, PRKCG, SLC12A5, SLC32A1, SLC6A13 |

**Table S5.** Cross-talk genes involved in different pathways.

| Cross-talk genes | Involved pathways |
| --- | --- |
| TP53 | 6 |
| CDK2 | 5 |
| GADD45G | 5 |
| MYC | 5 |
| CDK4 | 5 |
| CDK1 | 4 |
| CCNB2 | 4 |
| E2F2 | 4 |
| IGFBP3 | 3 |
| CHEK2 | 3 |
| CDC25A | 3 |
| CCNA2 | 3 |
| PLK1 | 3 |
| BUB1 | 3 |
| PKMYT1 | 3 |
| CDC25C | 3 |
| ESPL1 | 2 |
| AURKA | 2 |
| PTTG1 | 2 |
| CDKN2C | 2 |
| MMP9 | 2 |

**Table S6.** Hub genes in the PPI network with degree more than 100.

| Gene | Degree | Eccentricity | Betweenness | Stress | Bridging |
| --- | --- | --- | --- | --- | --- |
| TOP2A | 175 | 0.143 | 161612.079 | 1685288 | 48.377 |
| TP53 | 164 | 0.143 | 222651.923 | 2444056 | 55.923 |
| CDK1 | 142 | 0.143 | 31776.505 | 531102 | 50.810 |
| PLK1 | 125 | 0.143 | 19537.441 | 359950 | 38.983 |
| KIF11 | 117 | 0.125 | 8299.370 | 173348 | 27.295 |
| CCNA2 | 116 | 0.143 | 4524.299 | 122158 | 16.485 |
| AURKB | 116 | 0.143 | 8694.535 | 197356 | 25.932 |
| BUB1B | 112 | 0.125 | 6388.126 | 123154 | 20.503 |
| GNG13 | 112 | 0.143 | 36831.355 | 590958 | 51.117 |
| BUB1 | 110 | 0.143 | 2338.703 | 93606 | 11.126 |
| CCNB2 | 109 | 0.125 | 3930.065 | 116262 | 16.328 |
| AURKA | 109 | 0.143 | 8619.795 | 177360 | 26.321 |
| KIF2C | 109 | 0.125 | 10083.665 | 166406 | 28.427 |
| KIF23 | 109 | 0.125 | 15231.129 | 239798 | 48.161 |
| GNG3 | 108 | 0.143 | 26476.991 | 446532 | 68.567 |
| NDC80 | 106 | 0.143 | 13609.784 | 182614 | 63.567 |
| RAD51 | 105 | 0.143 | 29385.247 | 395420 | 61.100 |
| CENPA | 104 | 0.125 | 4419.546 | 124346 | 24.071 |
| PBK | 102 | 0.143 | 15630.531 | 279746 | 56.981 |
| BIRC5 | 101 | 0.143 | 20044.927 | 338840 | 92.026 |
| KIF20A | 101 | 0.143 | 3094.536 | 95522 | 18.307 |
| UBE2C | 101 | 0.125 | 11766.081 | 147264 | 51.551 |
| MELK | 101 | 0.125 | 6181.536 | 175118 | 38.081 |
| CENPE | 100 | 0.125 | 3020.517 | 101626 | 17.364 |
| TTK | 100 | 0.125 | 8346.376 | 121474 | 27.819 |

**Table S7.** Differentially expressed miRNAs between normal brain and glioma samples.

| MiRNA | logFC | PValue | FDR |
| --- | --- | --- | --- |
| hsa-mir-139 | -2.22903 | 2.94E-09 | 8.51E-07 |
| hsa-mir-497 | 2.435766 | 3.36E-09 | 8.51E-07 |
| hsa-mir-137 | -2.84561 | 5.49E-09 | 9.28E-07 |
| hsa-mir-380 | -2.26837 | 9.62E-09 | 1.22E-06 |
| hsa-mir-485 | -2.41673 | 1.39E-08 | 1.41E-06 |
| hsa-mir-432 | -2.21889 | 1.82E-08 | 1.51E-06 |
| hsa-mir-889 | -2.29733 | 2.09E-08 | 1.51E-06 |
| hsa-mir-132 | -1.76551 | 2.57E-08 | 1.63E-06 |
| hsa-mir-329-2 | -2.39112 | 3.21E-08 | 1.81E-06 |
| hsa-mir-769 | -1.56213 | 4.29E-08 | 2.17E-06 |
| hsa-mir-495 | -2.15833 | 6.98E-08 | 3.22E-06 |
| hsa-mir-329-1 | -2.29903 | 9.02E-08 | 3.81E-06 |
| hsa-mir-629 | 2.260557 | 1.36E-07 | 5.19E-06 |
| hsa-mir-433 | -2.46723 | 1.44E-07 | 5.19E-06 |
| hsa-mir-7-3 | -2.60004 | 1.56E-07 | 5.19E-06 |
| hsa-mir-181a-2 | 2.582707 | 1.64E-07 | 5.19E-06 |
| hsa-mir-218-2 | -2.45658 | 2.86E-07 | 8.54E-06 |
| hsa-mir-93 | 2.053285 | 4.15E-07 | 1.17E-05 |
| hsa-mir-3074 | 3.32245 | 4.58E-07 | 1.22E-05 |
| hsa-mir-935 | -1.69703 | 4.91E-07 | 1.24E-05 |
| hsa-mir-3200 | -1.54183 | 5.24E-07 | 1.27E-05 |
| hsa-mir-487b | -2.0876 | 8.08E-07 | 1.79E-05 |
| hsa-mir-543 | -1.94812 | 8.13E-07 | 1.79E-05 |
| hsa-mir-138-2 | -2.00057 | 1.09E-06 | 2.17E-05 |
| hsa-mir-9-1 | 2.383284 | 1.09E-06 | 2.17E-05 |
| hsa-mir-9-2 | 2.38303 | 1.11E-06 | 2.17E-05 |
| hsa-mir-195 | 2.148532 | 1.49E-06 | 2.79E-05 |
| hsa-mir-1224 | -2.26859 | 1.62E-06 | 2.93E-05 |
| hsa-mir-129-2 | -2.19336 | 1.78E-06 | 3.11E-05 |
| hsa-mir-656 | -2.2031 | 1.87E-06 | 3.16E-05 |
| hsa-mir-129-1 | -2.17683 | 2.4E-06 | 3.93E-05 |
| hsa-mir-381 | -1.88124 | 2.54E-06 | 4.02E-05 |
| hsa-mir-369 | -1.86311 | 3.29E-06 | 5.06E-05 |
| hsa-mir-7-2 | -2.17197 | 3.53E-06 | 5.27E-05 |
| hsa-mir-370 | -1.85058 | 3.89E-06 | 5.61E-05 |
| hsa-mir-873 | -2.65886 | 4.02E-06 | 5.61E-05 |
| hsa-mir-128-2 | -1.70465 | 4.1E-06 | 5.61E-05 |
| hsa-mir-744 | -1.16442 | 5.29E-06 | 7.06E-05 |
| hsa-mir-1250 | -1.79727 | 6.98E-06 | 9.07E-05 |
| hsa-mir-1249 | -1.5896 | 7.98E-06 | 0.000101 |
| hsa-mir-758 | -1.83695 | 8.67E-06 | 0.000107 |
| hsa-mir-496 | -1.94527 | 9.2E-06 | 0.000111 |
| hsa-mir-541 | -2.04606 | 0.00001 | 0.000118 |
| hsa-mir-154 | -1.71381 | 1.07E-05 | 0.000121 |
| hsa-mir-876 | -2.65834 | 1.08E-05 | 0.000121 |
| hsa-mir-323 | -1.96968 | 1.35E-05 | 0.000147 |
| hsa-mir-874 | -1.42343 | 1.36E-05 | 0.000147 |
| hsa-mir-136 | -1.72887 | 1.43E-05 | 0.000151 |
| hsa-mir-135a-1 | 2.465378 | 1.47E-05 | 0.000152 |
| hsa-mir-668 | -2.05981 | 0.000015 | 0.000152 |
| hsa-mir-410 | -2.01096 | 1.54E-05 | 0.000154 |
| hsa-mir-10b | 8.431847 | 2.29E-05 | 0.000223 |
| hsa-mir-28 | 1.457535 | 2.78E-05 | 0.000265 |
| hsa-mir-766 | -1.55257 | 2.82E-05 | 0.000265 |
| hsa-mir-92b | 2.095623 | 0.000029 | 0.000267 |
| hsa-mir-532 | 1.688169 | 3.14E-05 | 0.000285 |
| hsa-mir-128-1 | -1.49581 | 3.39E-05 | 0.000301 |
| hsa-mir-660 | 1.867316 | 3.94E-05 | 0.000345 |
| hsa-mir-770 | -2.21176 | 4.25E-05 | 0.000365 |
| hsa-mir-584 | -1.9043 | 4.37E-05 | 0.00037 |
| hsa-mir-411 | -1.61519 | 4.68E-05 | 0.000384 |
| hsa-mir-130b | 2.057509 | 0.000047 | 0.000384 |
| hsa-mir-412 | -2.21666 | 4.95E-05 | 0.000398 |
| hsa-mir-138-1 | -1.73115 | 5.38E-05 | 0.000426 |
| hsa-mir-379 | -1.72011 | 5.83E-05 | 0.000455 |
| hsa-mir-19b-1 | 1.921238 | 6.27E-05 | 0.000482 |
| hsa-mir-27a | 1.97306 | 7.85E-05 | 0.000588 |
| hsa-mir-127 | -1.55766 | 7.89E-05 | 0.000588 |
| hsa-mir-30a | 1.49919 | 8.07E-05 | 0.000593 |
| hsa-mir-1185-1 | -1.69686 | 8.76E-05 | 0.000635 |
| hsa-mir-3943 | -1.73138 | 0.000105 | 0.000746 |
| hsa-mir-374a | 1.70054 | 0.000106 | 0.000747 |
| hsa-mir-891a | 5.038088 | 0.000112 | 0.000768 |
| hsa-mir-23a | 1.818401 | 0.000112 | 0.000768 |
| hsa-mir-490 | -1.98791 | 0.000128 | 0.000866 |
| hsa-mir-221 | -1.54039 | 0.000134 | 0.000885 |
| hsa-mir-3647 | 3.173503 | 0.000135 | 0.000885 |
| hsa-mir-17 | 1.383377 | 0.000136 | 0.000885 |
| hsa-mir-382 | -1.47235 | 0.000146 | 0.000936 |
| hsa-mir-20a | 1.861367 | 0.000159 | 0.001007 |
| hsa-mir-338 | -1.57118 | 0.000163 | 0.001013 |
| hsa-mir-377 | -1.58035 | 0.000164 | 0.001013 |
| hsa-mir-25 | 1.583582 | 0.00018 | 0.001095 |
| hsa-mir-183 | 4.701216 | 0.000181 | 0.001095 |
| hsa-mir-7-1 | -1.29196 | 0.000189 | 0.001129 |
| hsa-mir-3607 | 2.505089 | 0.000192 | 0.001132 |
| hsa-mir-106b | 1.345704 | 0.000195 | 0.001135 |
| hsa-mir-374b | 1.320345 | 0.000201 | 0.00116 |
| hsa-mir-892a | 5.066042 | 0.000205 | 0.001166 |
| hsa-mir-299 | -1.53312 | 0.000217 | 0.001223 |
| hsa-mir-24-1 | -1.06807 | 0.00023 | 0.001268 |
| hsa-mir-431 | -1.80939 | 0.00023 | 0.001268 |
| hsa-mir-212 | -1.46019 | 0.000238 | 0.001299 |
| hsa-mir-3653 | 2.579436 | 0.000242 | 0.001306 |
| hsa-mir-424 | 2.039618 | 0.000261 | 0.001389 |
| hsa-mir-502 | 1.391044 | 0.000266 | 0.001391 |
| hsa-mir-625 | -1.21991 | 0.000302 | 0.001565 |
| hsa-mir-891b | 5.175557 | 0.000327 | 0.001673 |
| hsa-mir-888 | 5.412501 | 0.000339 | 0.00172 |
| hsa-mir-130a | 1.310361 | 0.000358 | 0.001799 |
| hsa-mir-92a-2 | 1.342606 | 0.000383 | 0.001902 |
| hsa-mir-551b | 2.705537 | 0.000405 | 0.001991 |
| hsa-mir-491 | -1.36267 | 0.000408 | 0.001991 |
| hsa-mir-505 | 1.416015 | 0.000421 | 0.002034 |
| hsa-mir-642a | -1.71225 | 0.000436 | 0.002085 |
| hsa-mir-182 | 4.175044 | 0.000444 | 0.002104 |
| hsa-mir-222 | -1.44628 | 0.00045 | 0.002112 |
| hsa-mir-188 | 1.996596 | 0.000458 | 0.002131 |
| hsa-let-7i | 1.115669 | 0.000503 | 0.002319 |
| hsa-mir-9-3 | 1.991559 | 0.000524 | 0.002395 |
| hsa-mir-3913-1 | 2.092664 | 0.000586 | 0.002651 |
| hsa-mir-450a-2 | 2.277661 | 0.000647 | 0.002901 |
| hsa-mir-500a | 1.524816 | 0.000667 | 0.002967 |
| hsa-mir-219-1 | 2.344154 | 0.000723 | 0.003186 |
| hsa-mir-628 | -1.1549 | 0.000735 | 0.003212 |
| hsa-mir-330 | -1.37115 | 0.000783 | 0.003394 |
| hsa-mir-487a | -1.53537 | 0.000827 | 0.003553 |
| hsa-mir-99a | 1.556061 | 0.000897 | 0.003822 |
| hsa-mir-892b | 4.904581 | 0.000937 | 0.003926 |
| hsa-mir-320b-2 | 1.75694 | 0.000962 | 0.003996 |
| hsa-mir-1185-2 | -1.62054 | 0.001065 | 0.004391 |
| hsa-mir-1258 | -1.87498 | 0.001129 | 0.004618 |
| hsa-mir-550a-1 | 1.598266 | 0.001169 | 0.004742 |
| hsa-mir-19b-2 | 1.461892 | 0.001182 | 0.004755 |
| hsa-mir-320c-1 | 2.495379 | 0.001197 | 0.00478 |
| hsa-mir-1197 | -1.75767 | 0.001321 | 0.005233 |
| hsa-mir-500b | 1.296083 | 0.001369 | 0.00538 |
| hsa-mir-296 | 1.790638 | 0.001444 | 0.00563 |
| hsa-mir-655 | -1.37277 | 0.001472 | 0.005696 |
| hsa-mir-320a | 1.53792 | 0.001486 | 0.005707 |
| hsa-mir-1229 | -1.34807 | 0.001505 | 0.005738 |
| hsa-mir-96 | 3.971525 | 0.001877 | 0.006999 |
| hsa-mir-550a-2 | 1.728813 | 0.001903 | 0.007043 |
| hsa-mir-454 | 1.494443 | 0.001958 | 0.007194 |
| hsa-mir-450b | 2.02746 | 0.002033 | 0.007415 |
| hsa-mir-450a-1 | 1.93161 | 0.002084 | 0.007549 |
| hsa-mir-542 | 1.71763 | 0.002249 | 0.008029 |
| hsa-let-7b | 1.174442 | 0.002466 | 0.008622 |
| hsa-mir-3065 | 2.203735 | 0.002553 | 0.008867 |
| hsa-mir-3193 | 2.431366 | 0.002673 | 0.009157 |
| hsa-mir-409 | -1.24529 | 0.003784 | 0.01262 |
| hsa-mir-376a-1 | -1.30542 | 0.003884 | 0.012869 |
| hsa-mir-539 | -1.4398 | 0.004415 | 0.014441 |
| hsa-mir-708 | 1.282444 | 0.004478 | 0.014555 |
| hsa-mir-301a | 1.414744 | 0.004551 | 0.014697 |
| hsa-mir-92a-1 | 1.290398 | 0.005079 | 0.016298 |
| hsa-mir-21 | 2.624298 | 0.00547 | 0.017442 |
| hsa-mir-570 | 2.197521 | 0.005894 | 0.018611 |
| hsa-mir-124-1 | -1.52202 | 0.00592 | 0.018611 |
| hsa-mir-124-2 | -1.51452 | 0.006177 | 0.019097 |
| hsa-mir-124-3 | -1.50853 | 0.006452 | 0.019824 |
| hsa-mir-149 | -1.04324 | 0.0066 | 0.020114 |
| hsa-mir-181d | 1.126262 | 0.006625 | 0.020114 |
| hsa-mir-26a-1 | 1.882162 | 0.006849 | 0.020669 |
| hsa-mir-320c-2 | 2.613029 | 0.007098 | 0.021294 |
| hsa-mir-548v | -1.12747 | 0.007171 | 0.021386 |
| hsa-mir-323b | -1.41762 | 0.007586 | 0.022491 |
| hsa-mir-552 | -1.16328 | 0.00821 | 0.024199 |
| hsa-mir-2114 | 4.072561 | 0.008856 | 0.025954 |
| hsa-mir-3677 | 1.671437 | 0.009235 | 0.026826 |
| hsa-mir-1-2 | -1.26755 | 0.00926 | 0.026826 |
| hsa-mir-3622a | 1.872564 | 0.009486 | 0.027328 |
| hsa-mir-639 | 2.826731 | 0.009771 | 0.027988 |
| hsa-mir-548f-1 | 3.563392 | 0.009904 | 0.028059 |
| hsa-mir-1243 | -1.05428 | 0.009907 | 0.028059 |
| hsa-mir-501 | 1.215573 | 0.010735 | 0.030236 |
| hsa-mir-146a | 1.375676 | 0.012414 | 0.034773 |
| hsa-mir-203 | -1.28842 | 0.01263 | 0.035184 |
| hsa-mir-3651 | 2.841899 | 0.013052 | 0.036156 |
| hsa-mir-134 | -1.05312 | 0.01468 | 0.040232 |
| hsa-mir-3170 | 2.012298 | 0.014948 | 0.040746 |
| hsa-mir-224 | 2.677256 | 0.016271 | 0.044115 |
| hsa-mir-362 | 1.32949 | 0.017439 | 0.046782 |
